# A verified genomic reference sample for assessing performance of cancer panels detecting small variants of low allele frequency

**DOI:** 10.1186/s13059-021-02316-z

**Published:** 2021-04-16

**Authors:** Wendell Jones, Binsheng Gong, Natalia Novoradovskaya, Dan Li, Rebecca Kusko, Todd A. Richmond, Donald J. Johann, Halil Bisgin, Sayed Mohammad Ebrahim Sahraeian, Pierre R. Bushel, Mehdi Pirooznia, Katherine Wilkins, Marco Chierici, Wenjun Bao, Lee Scott Basehore, Anne Bergstrom Lucas, Daniel Burgess, Daniel J. Butler, Simon Cawley, Chia-Jung Chang, Guangchun Chen, Tao Chen, Yun-Ching Chen, Daniel J. Craig, Angela del Pozo, Jonathan Foox, Margherita Francescatto, Yutao Fu, Cesare Furlanello, Kristina Giorda, Kira P. Grist, Meijian Guan, Yingyi Hao, Scott Happe, Gunjan Hariani, Nathan Haseley, Jeff Jasper, Giuseppe Jurman, David Philip Kreil, Paweł Łabaj, Kevin Lai, Jianying Li, Quan-Zhen Li, Yulong Li, Zhiguang Li, Zhichao Liu, Mario Solís López, Kelci Miclaus, Raymond Miller, Vinay K. Mittal, Marghoob Mohiyuddin, Carlos Pabón-Peña, Barbara L. Parsons, Fujun Qiu, Andreas Scherer, Tieliu Shi, Suzy Stiegelmeyer, Chen Suo, Nikola Tom, Dong Wang, Zhining Wen, Leihong Wu, Wenzhong Xiao, Chang Xu, Ying Yu, Jiyang Zhang, Yifan Zhang, Zhihong Zhang, Yuanting Zheng, Christopher E. Mason, James C. Willey, Weida Tong, Leming Shi, Joshua Xu

**Affiliations:** 1 Q2 Solutions - EA Genomics, 5927 S Miami Blvd., Morrisville, NC 27560 USA; 2 Division of Bioinformatics and Biostatistics, National Center for Toxicological Research, US Food and Drug Administration, Jefferson, AR 72079 USA; 3 Agilent Technologies, 11011 N Torrey Pines Rd., La Jolla, CA 92037 USA; 4 Immuneering Corporation, One Broadway, 14th Floor, Cambridge, MA 02142 USA; 5 Market & Application Development Bioinformatics, Roche Sequencing Solutions Inc., 4300 Hacienda Dr., Pleasanton, CA 94588 USA; 6 Winthrop P Rockefeller Cancer Institute, University of Arkansas for Medical Sciences, 4301 W Markham St., Little Rock, AR 72205 USA; 7 Department of Computer Science, Engineering and Physics, University of Michigan-Flint, Flint, MI 48502 USA; 8 Bioinformatics Research & Early Development, Roche Sequencing Solutions Inc., 1301 Shoreway Rd., Suite 7 #300, Belmont, CA 94002 USA; 9 National Institute of Environmental Health Sciences, Research Triangle Park, Durham, NC 27709 USA; 10 Bioinformatics and Computational Biology Laboratory, National Heart Lung and Blood Institute, National Institutes of Health, Bethesda, MD 20892 USA; 11 Agilent Technologies, 5301 Stevens Creek Blvd., Santa Clara, CA 95051 USA; 12 Fondazione Bruno Kessler, 38123 Trento, Italy; 13 JMP Life Sciences, SAS Institute Inc., Cary, NC 27519 USA; 14 (formerly) Research and Development, Roche Sequencing Solutions Inc., 500 South Rosa Rd., Madison, WI 53719 USA; 15 Department of Physiology and Biophysics, Weill Cornell Medicine, Cornell University, New York, NY 10065 USA; 16 (formerly) Clinical Sequencing Division, Thermo Fisher Scientific, 180 Oyster Point Blvd., South San Francisco, CA 94080 USA; 17 Stanford Genome Technology Center, Stanford University, Palo Alto, CA 94304 USA; 18 Department of Immunology, Genomics and Microarray Core Facility, University of Texas Southwestern Medical Center, 5323 Harry Hine Blvd., Dallas, TX 75390 USA; 19 University of Texas Southwestern Medical Center, 2330 Inwood Rd., Dallas, TX 75390 USA; 20 Department of Medicine, College of Medicine and Life Sciences, The University of Toledo, Toledo, OH 43614 USA; 21 Institute of Medical and Molecular Genetics (INGEMM), Hospital Universitario La Paz, CIBERER Instituto de Salud Carlos III, 28046 Madrid, Spain; 22 Thermo Fisher Scientific, 110 Miller Ave., Ann Arbor, MI 48104 USA; 23 Marketing, Integrated DNA Technologies, Inc., 1710 Commercial Park, Coralville, IA 52241 USA; 24 College of Chemistry, Sichuan University, Chengdu, 610064 Sichuan China; 25 Agilent Technologies, 1834 State Hwy 71 West, Cedar Creek, TX 78612 USA; 26 Illumina Inc., 5200 Illumina Way, San Diego, CA 92122 USA; 27 Bioinformatics Research, Institute of Molecular Biotechnology, Boku University Vienna, Vienna, Austria; 28 Małopolska Centre of Biotechnology, Jagiellonian University, Krakow, Poland; 29 Department of Biotechnology, Boku University, Vienna, Austria; 30 Bioinformatics, Integrated DNA Technologies, Inc., 1710 Commercial Park, Coralville, IA 52241 USA; 31 Kelly Government Solutions, Inc., Research Triangle Park, NC 27709 USA; 32 Center of Genome and Personalized Medicine, Institute of Cancer Stem Cell, Dalian Medical University, Dalian, Liaoning China; 33 EATRIS ERIC- European Infrastructure for Translational Medicine, De Boelelaan 1118, 1081 HZ Amsterdam, The Netherlands; 34 Division of Genetic and Molecular Toxicology, National Center for Toxicological Research, US Food and Drug Administration, Jefferson, AR 72079 USA; 35 Research and Development, Burning Rock Biotech, Shanghai, 201114 China; 36 Institute for Molecular Medicine Finland (FIMM), Nordic EMBL Partnership for Molecular Medicine, HiLIFE Unit, Biomedicum Helsinki 2U (D302b), FI-00014 University of Helsinki, P.O. Box 20 (Tukholmankatu 8), Helsinki, Finland; 37 Center for Bioinformatics and Computational Biology, and the Institute of Biomedical Sciences, School of Life Sciences, East China Normal University, 500 Dongchuan Rd, Shanghai, 200241 China; 38 University of North Carolina Health, 101 Manning Drive, Chapel Hill, NC 27514 USA; 39 Department of Epidemiology, School of Public Health, Fudan University, Shanghai, China; 40 Center of Molecular Medicine, Central European Institute of Technology, Masaryk University, Kamenice 5, 625 00 Brno, Czech Republic; 41 Massachusetts General Hospital, Harvard Medical School, Boston, MA 02114 USA; 42 Research and Development, QIAGEN Sciences Inc., Frederick, MD 21703 USA; 43 State Key Laboratory of Genetic Engineering, School of Life Sciences and Shanghai Cancer Hospital/Cancer Institute, Fudan University, Shanghai, 200438 China; 44 University of Arkansas at Little Rock, Little Rock, AR 72204 USA; 45 Departments of Medicine, Pathology, and Cancer Biology, College of Medicine and Life Sciences, University of Toledo Health Sciences Campus, 3000 Arlington Ave, Toledo, OH 43614 USA; 46 Human Phenome Institute, Fudan University, Shanghai, 201203 China; 47 Fudan-Gospel Joint Research Center for Precision Medicine, Fudan University, Shanghai, 200438 China

## Abstract

**Background:**

Oncopanel genomic testing, which identifies important somatic variants, is increasingly common in medical practice and especially in clinical trials. Currently, there is a paucity of reliable genomic reference samples having a suitably large number of pre-identified variants for properly assessing oncopanel assay analytical quality and performance. The FDA-led Sequencing and Quality Control Phase 2 (SEQC2) consortium analyze ten diverse cancer cell lines individually and their pool, termed Sample A, to develop a reference sample with suitably large numbers of coding positions with known (variant) positives and negatives for properly evaluating oncopanel analytical performance.

**Results:**

In reference Sample A, we identify more than 40,000 variants down to 1% allele frequency with more than 25,000 variants having less than 20% allele frequency with 1653 variants in COSMIC-related genes. This is 5–100× more than existing commercially available samples. We also identify an unprecedented number of negative positions in coding regions, allowing statistical rigor in assessing limit-of-detection, sensitivity, and precision. Over 300 loci are randomly selected and independently verified via droplet digital PCR with 100% concordance. Agilent normal reference Sample B can be admixed with Sample A to create new samples with a similar number of known variants at much lower allele frequency than what exists in Sample A natively, including known variants having allele frequency of 0.02%, a range suitable for assessing liquid biopsy panels.

**Conclusion:**

These new reference samples and their admixtures provide superior capability for performing oncopanel quality control, analytical accuracy, and validation for small to large oncopanels and liquid biopsy assays.

**Supplementary Information:**

The online version contains supplementary material available at 10.1186/s13059-021-02316-z.

## Introduction

Recent Sequencing and Quality Control Phase 2 (SEQC2) [1] consortium efforts have engaged in determining samples and methods for DNA-based NGS testing for a variety of translational and precision medicine applications. Reported here from SEQC2 consortium members are methods and archetypes suitable for establishing a reliable, robust, continuous, and generally available genomics reference samples that can be used for assessing analytical performance of next-generation sequencing (NGS) assays across a wide range of testing scenarios, especially in cancer and including those involving regulatory science and precision medicine.

Genomic testing of tumors to determine important somatic variants in cancer is becoming more commonplace in medical practice and especially in clinical trials. Currently, there is a paucity of reliable genomic reference samples that can be used as a standard across a wide range of genomic testing methods for assessing the potential accuracy and the overall analytical performance of a given assay. The National Institute for Standards and Technology (NIST) has developed several cell lines of reference material for testing population genetics, which translates into a very high percentage of variants inherently at 50% and 100% allele frequency in germline cells [2]. However, these samples are not appropriate in their current form for comprehensively evaluating the analytical performance of cancer panels as somatic mutations often at lower than 20% variant allele frequency (VAF). Other samples including reference standards developed for somatic mutation typically involve at most one cancer cell line and a matching normal, greatly limiting the number of relevant variants at low VAF available for evaluation [3–6]. While these reference standards are valuable and provide utility in several NGS contexts, proper comprehensive assessment of cancer panels typically requires more than 100 appropriate qualitative analytes (such as a variant detected/not detected) in each of several distinct VAF ranges, which is more than what many general and commercially available reference samples have in total. Finally, the overall variant detection performance is inversely related to the VAF of the analytes targeted. To determine panel performance at different VAF magnitudes, reference samples should have large numbers of variants at various VAF magnitudes.

Commonly available commercial reference samples typically have less than 100 variants in a relatively small number of genes with allele frequencies suitable for pan-cancer panel validation. More recently, Horizon Diagnostics released the OncoSpan reference standard with 386 variants across 152 (114 COSMIC tier 1) genes [7]. Acrometrix released the Oncology Hotspot Control which has 555 variants across 53 (52 COSMIC tier 1) genes [8]. As useful as each of these reference samples are, even if both are used in panel testing validation, only 127 out of the 576 COSMIC Tier1 genes will have at least one variant in either panel and only 53 genes would have any variant from a COSMIC Tier 1 gene with a VAF less than 20%. Large cancer panels, such as the Illumina TruSight Oncology 500 (TSO500) panel, would only have a minority of genes (121 genes total or less than 25% of TSO500) that could be tested within the panel. With regard to the greater human exome, current reference samples including the NIST Genome-in-a-Bottle (GIAB) references provide too few variants per gene (typically ~ 1 variant per gene in coding regions) and only at high VAF (~ 50% and ~ 100%). Using NIST or similar reference standards for oncopanel testing would require potentially complex admixtures of several distinct samples to create a wide range of variants at various VAF ranges, a complicated process for even experienced laboratories.

Current needs in genomics testing are not limited to additional appropriate reference samples but also include describing appropriate methods and guidelines for developing and verifying such a sample. Given the complexity and the magnitude of the number of variants required for a reference sample, it is straightforward to conclude that the most efficient development method is to make use of more easily identified germline variants that are diluted in some fashion to resemble somatic changes. This is reasonable as germline variants originated as a founder mutation and assessing analytical performance of an assay can be independent of the biological impact of the variant.

The SEQC2 consortium, led by the Food and Drug Administration (FDA), is a continuation of successful prior efforts in examining methods and reproducibility in genomics and transcriptomics [9–13]. The Oncopanel Sequencing Working Group within SEQC2 was challenged with examining the reproducibility, sensitivity, and accuracy of current (or in development) commercially available pan-cancer tumor panels for both solid tumors and for liquid biopsies. We ascertained the need to (1) identify “ground truth” of an unprecedented number of low VAF variants and invariant positions in a DNA reference sample and (2) utilize the crowdsource effort of the SEQC2 consortium to undertake massive data generation, management, analysis, and compilation of results. To that end, genomic DNA samples from ten cancer cell lines historically used to create RNA for the Agilent Universal Human Reference (UHR) RNA sample [14] were examined individually as well as in pooled form (termed “Sample A”) to develop a reference set (positives and negatives) for use with tumor cancer panels (Table 1). Furthermore, a cell line derived from a normal male individual (Agilent OneSeq Human Reference DNA, PN 5190–8848) (termed “Sample B”) was similarly characterized serving primarily as a negative control for somatic variants but also providing a genomic background for mixtures between the two reference samples. In particular, we used Sample B to dilute mutually disjoint variants in Sample A to a much lower VAF. Thus, the pooled Sample A used in tandem with Sample B are suitable for panel development, panel validation, and for quality control (high number of known positives and negatives) in parallel with an operational assay, allowing testing of large numbers of variants across a wide range of allele frequencies.

**Table 1:** List and description of 10 cancer cell lines and a normal reference cell line with % estimated copy number alterations (CNA) and an intra-tumor heterogeneity (ITH) value to indicate potential polyclonality of the cell line

Cell line	Name	Description	Comments	CNA % (est.)^a^	ITH (Shannon’s index)
B	Male reference	Normal		~ 0	0
BLY	B lymphocyte	Myeloma	Mixed with TLY within study^b^	~ 25%	43.2
BRA	Brain	Glioblastoma	Polyclonal	90%	21.0
BRE	Breast	Adenocarcinoma	Polyclonal	60%	100.0
CRV	Cervix	Adenocarcinoma	Polyclonal	70%	10.9
LIP	Soft tissue	Liposarcoma		90%	3.5
LIV	Liver	Hepatoblastoma		27%	2.5
MAC	Macrophage	Lymphoma	Polyclonal	80%	11.8
SKN	Skin	Melanoma		24%	0
TES	Testes	Carcinoma		72%	4.8
TLY	T lymphoblast	Leukemia	Inherently tetraploid^c^ with variations	22%	1.1

^a^ CNA were estimated using Agilent GenetiSure Cancer Research CGH + SNP Microarray (2 × 400K), G5975A and WES

^b^ Information of the mixture of the original BLY with TLY is provided in Supplementary Information

^c^ Establishment of the tetraploid nature of the TLY is provided in Supplementary Information

## Results

### Identification of positives and negatives in the reference sample

**Fig. 1:**
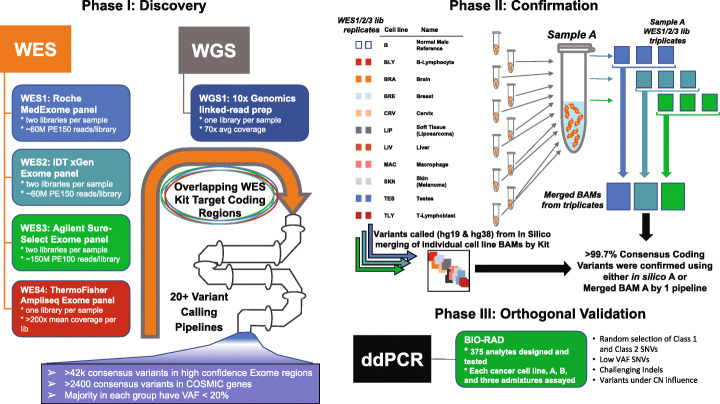
Overall flow diagram of process/method. Discovery of Class 1 variants came from consensus analysis of WES1/2/3/4 runs on overlapping WES kit target regions having high confidence. Additional Class 2 variants were discovered after analyzing WGS1 with WES results. Variants were confirmed by analyzing in silico A results where we combined individual BAMs from each cell line replicate and by analyzing merged-BAM Sample A from pooled Sample A individual replicate BAMs. Finally, a subset of these variants was orthogonally validated with ddPCR

This study utilized four different whole exome sequencing (WES) enrichment kits and one whole genome sequencing (WGS) method: (i) Roche MedExome [15] (WES1), (ii) Integrated DNA Technologies (IDT) xGen [16] (WES2), (iii) Agilent SureSelect [17] (WES3), (iv) Thermo Fisher AmpliSeq Exome [18] (WES4), and the 10X Genomics linked-read WGS sequencing [19] (WGS1). Details of the experimental design are shown in Fig. 1. Replicate libraries were created by independent labs for each individual cell line and sequenced to high depth for WES1–3 methods (WES deduplicated libraries had a typical depth of 235× with an average depth range from 151× to 402× in their targeted regions). WES4 and WGS1 were sequenced at lower depth without library replicates (the typical depth was 148× for WES4 with a range across cell lines of 116–185× while the average depth for WGS1 ranged from 64.5× to 74.9× across cell lines). As these 10 cell lines were to be pooled together, we sequenced each cell line to sufficient depth to ensure high sensitivity to detect variants above 10% VAF by cell line, which would generally result in a collection of identified variants down to 1% VAF in the pooled Sample A. Details related to the sequencing of the individual cell line libraries and their read characteristics are shown in Additional file 1: Table S1.

FASTQ files from these libraries were provided to the working group participants for variant calling by an assortment of bioinformatic pipelines (each site ran only pipelines for WES or WGS data for which they were proficient). We created a reference bed file that was the intersection of the design bed files of each enrichment kit (termed the “Interval4” bed file) so that evaluation of the putative variants would potentially have representative data from all four exome enrichment kits as well as WGS. The design size of the regions-of-interest (ROI) of each kit relative to the known exome and their overlap are provided in Additional file 1: Table S2. Variant calling methods, which included GATK Haplotyper [20], FreeBayes [21], Mutect1 and Mutect2 [22], Platypus [23], Samtools [24], Sentieon TNscope [25], VarDict [26], VarScan [27], and SomaticSeq [28], were combined with different alignment strategies (bwa-mem [29], bwa [30], bowtie2 [31], etc.) to create a comprehensive set of putative variants. An overview of each bioinformatic pipeline and WES kit combination is provided in Additional file 1: Table S3.

**Fig. 2:**
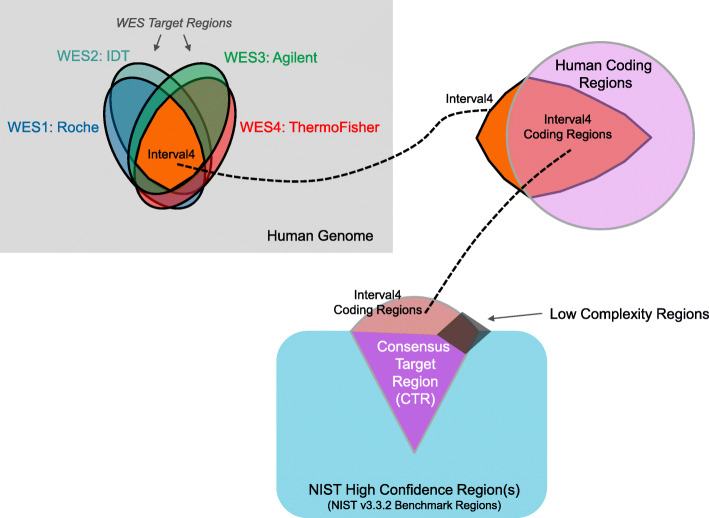
Defining the consensus target region (CTR). The regions shown are not to scale. Most of these regions and their sizes are provided in Additional file 1: Table S2. The low complexity regions are excluded from the CTR. Importantly, the size of the CTR is ~ 22.7 Mb for hg19

To protect against a common bias towards false positive calls, the variant calls were filtered based on NIST high-confidence or benchmark regions [2] [“Methods” section: NIST high-confidence regions (v3.3.2 benchmark regions)] as well as identified low genomic complexity regions [32] that resulted in a consensus target region (CTR) as shown in Fig. 2. Within the CTR, a region that is roughly two thirds the size of the human exome coding regions, a set of known positive variants (termed Class 1 variants) were identified as being called in the majority of the consortium pipeline-library combinations for at least one cell line in each replicate of WES1, WES2, and WES3 (the replicated kits). More details are available in “Rules for determining positive variants” in the “Methods” section.

To further enrich the set of identified variants in important genomic regions, we also examined each cell line using the WGS1 linked-read method in COSMIC genes [33, 34]. We added 359 variants (termed Class 2 variants) to our set of Class 1 variants that were outside our CTR region but were in high-confidence coding regions of COSMIC genes. The Class 2 variants were included only if they were detected by WGS1 in at least one cell line by two different variant calling methods and detected by at least two WES kits. From Table 2, we see that over 10% of the identified variants having VAF <  20% in COSMIC genes are Class 2 even though they represent < 1% of positive variants overall. Using hg19, we identified 42,570 Class 1 and Class 2 variants in Sample A (28,064 having VAF less than 20%) with additional characteristics of these variants provided in Additional file 1: Table S4. Notably, 1653 variants are in COSMIC genes with < 20% VAF.

**Table 2:** Variant characteristics of Sample A compared to other reference material (Sample A results are based on hg19), both Class1 and Class2 variants combined

Reference material	Total variants identified	Total coding variants identified	Coding variantsin COSMIC genes	Total coding variants < 20% VAF	Total coding variants < 20% VAF in COSMIC genes	#genes with 1 or more variants	#COSMIC genes with 1 or more variants (Tier1/Tier2)
Sample A	42,570	42,570	2432	28,064	1653	12,238	422/102
SNV	42,021	42,021	2398	27,683	1624		
Indel	549	549	34	381	29		
Acrometrix	555	555	555	341^a^	341^a^	53	52/1
SNV	504	504	504	317	317		
MNV/Indel	2/49	2/49	2/49	0/24	0/24		
Oncospan	386	386	319	52	46	152	114/2
SNV	357	357	297	43	38		
Indel	30	30	22	9	8		
HCC1395 (somatic)	41,556	487	193	144	14	466	188
SNV	39,536	460	186	132	13		
Indel	2020	27	7	12	1		
HCC1395BL (germline)	3,577,254	21,755	NA	NA	NA	9566	NA
SNV	3,225,512	21,381					
Indel	351,742	374					

^a^Most of the Acrometrix variants are synthetic controls. Thus, it is possible to construct a version of the material where 524 of the 555 variants have a VAF < 20%

Pile-ups of each cell line were also examined to identify 10,229,649 negative variant loci (hg19) that were lacking any variant in the pooled Sample A (more details provided in “Rules for determining known negative variant positions”). Negative loci for pooled Sample A imply that if there is any somatic variant at those loci, it must have an observed AF at less than 0.25% in the pooled Sample A for each replicated kit.

To confirm these individual cell line results, we constructed independent triplicate libraries of the pooled Sample A using three of the original four WES kits (Roche MedExome, IDT xGen, Agilent SureSelect) and sequenced each library very deeply (deduplicated average depth of 180–580× depending on library) as shown on the right side of Fig. 1. The detailed sequencing QC statistics of pooled Sample A are shown in Additional file 1: Table S5 with a summary in Additional file 2: Fig. S1. Using SomaticSeq, we confirmed greater than 99.5% of the Class 1 and 2 variants identified from the individual cell line analysis were at or near the expected VAF in Sample A using either an in silico version of Sample A or the merged-BAM Sample A. SomaticSeq also confirmed 99.96% of all variants in some fashion from either individual cell lines, the merged-BAM Sample A, or the in silico version of Sample A (see Additional file 1: Table S6a. A merged-BAM of Sample A for a particular kit is a merging of the three independent replicate library deduplicated Sample A BAM files for each of WES1–3. The in silico version of Sample A is a merging of the deduplicated cell line libraries for each of WES1–3. We should note other pipelines besides SomaticSeq were also highly sensitive to the identified variants and exemplar results for certain combinations of pipelines and kits are shown in Additional file 1: Table S6b. In addition, although the WES4 Thermo Fisher runs had only one library per cell line and were not as deeply sequenced as WES1–3, over 97.8% of the CTR positives with Sample A VAF greater than 5% were detected in at least one cell line. Additional information on WES4-related sensitivity and precision of individual cell lines are provided in Additional file 2: Fig. S2.

Finally, we used droplet digital PCR (ddPCR) technology to orthogonally verify 284 positives and 39 negatives randomly chosen with stratification using different classes of variants in individual cell lines, reference Sample A, and normal Sample B. We also orthogonally tested those same variants using admixture reference samples C, D, and E where Sample C is a 1:1 dilution of A:B, Sample D is a 1:4 dilution, and Sample E is a 1:24 dilution. The verified variants included more difficult-to-detect variants such as 50 smaller insertions-deletions (indels), 35 of which had VAF < 10% and 95 other low-frequency variants (VAF < 5% in pooled Sample A) including 12 low VAF Class 2 SNVs. We also verified 20 variants that were under copy number (CN) influence (i.e., loci having detected CN changes in at least one of the 10 cell lines). While we identified a small number of variants near homopolymer regions (less than 0.1% of the total), we did not verify those variants independently using ddPCR but did ensure that all variants near homopolymer regions and having VAF above the limit of detection of linked-read WGS were detected by the linked-read WGS. Additional file 1: Table S7 provides a detailed breakdown of the classes and subclasses and the number of variants by class orthogonally validated by ddPCR.

**Fig. 3:**
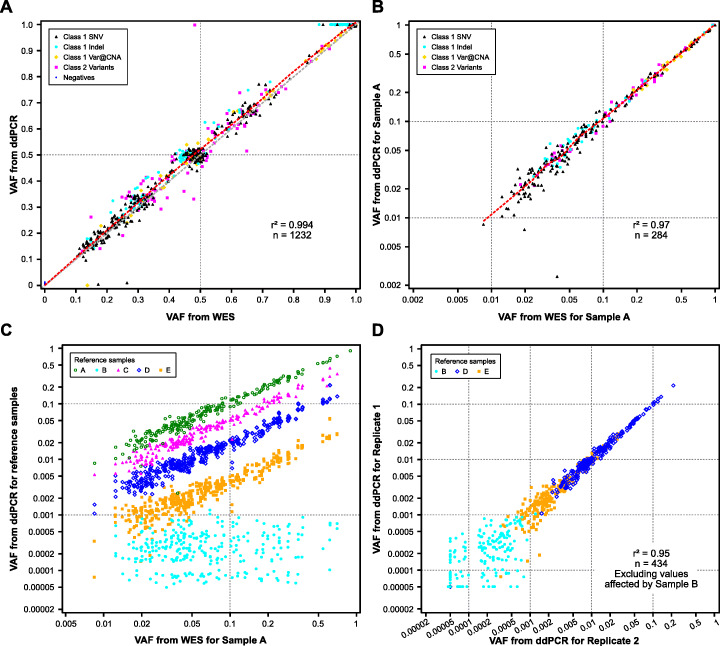
ddPCR and WES concordance: **a** VAF concordance of individual cell line WES consensus results with ddPCR assays of that cell line. **b** Concordance (log_10_ scale) of Sample A VAF between ddPCR and WES for positives only. **c** Various dilutions (C, D, E) of Sample A into B achieve the expected reduction in VAF as seen in the ddPCR results. It also shows the potential noise for measuring ddPCR variants below 0.1% (10^− 3^) in the distribution of Sample B variants. **d** Concordance of replicate ddPCR assays (on log_10_ scale) is very high (*r*^2^ = .95) in diluted target Samples D and E. Putative VAF values from Sample B are also shown for comparison

For the putative positives tested by ddPCR, 100% of the variants from the different classes were verified as positives with 99.65% having a concordant VAF value (i.e., within stochastic sampling noise) when comparing ddPCR and WES consensus VAF estimates for the individual cell lines and for Sample A (Fig. 3a, b; *r*^2^ = .994 on linear and *r*^2^ = .97 on log scale respectively). In addition, variants with VAF diluted through admixture changed in expected and predictable ways in concordance with the dilution level (Fig. 3c). For Samples D and E (the samples having the identified positive variants but at lower frequency), we also examined the concordance of replicate ddPCR wells along with concordance of the same variants in replicated Sample B assays (where no detection is expected). Figure 3d shows *r*^2^ = .95 for log (VAF) in replicate runs of Samples D and E and also shows uncorrelated VAF of those same variants in replicates of Sample B where they should not be present. In general, about half of the ddPCR assays showed noticeable variation between 0.1 and 0.01% VAF in Sample B for loci expected to be variant-free in Sample B, implying that the observed VAF is mostly background measurement error in ddPCR. This implies the positive variants are verified down to 0.1% in all reference samples but not necessarily below 0.1%. Negatives have the opposite characteristic: we are confident that the true VAF for each negative locus tested by ddPCR, if greater than 0, is below 0.1% VAF.

From this process, we identified 42,570 (hg19) and 38,957 (hg38) variants and more than 10,000,000 negative loci in the autosomal coding regions of the human genome for Sample A. We also identified 13901/12623 (hg19/hg38) positives for Sample B. Hg38 positives are noticeably less in number due to the requirement that positives be in NIST high-confidence (benchmark) regions for that reference version combined with over 2000 Sample A positives identified in high-confidence regions of hg19 not being in high-confidence regions of hg38 (even though the hg19 variants are easily mapped to hg38). The majority (~ 70%) of Sample A variants have a VAF below 20%. Of these 42,570 hg19 variants, 1809 were identified in COSMIC Tier 1 genes with 1255 having a VAF below 20%. The overwhelming majority of positives (98.7%) consist of single nucleotide variants (SNVs) with indels as a minority (1.3%) across the exome. Some details and characteristics of the variants identified from Sample A are provided in Table 2 relative to other reference samples.

**Fig. 4:**
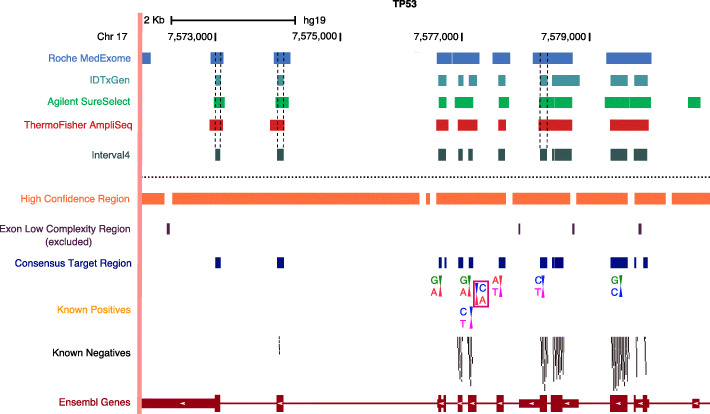
Illustration of considerations for determining positives and negatives within the reference material. Each WES kit coverage is shown relative to their intersection with coding regions (Interval4), the high confidence region, and the low complexity region. Also shown are known positive variant positions in Sample A (mostly Class 1 variants SNVs) including one identified by a violet box that is outside the Interval4 and CTR regions (Class 2 variant). Other positions shown include known negative positions

An illustrative example of the various design considerations, constraints, and information available (including positive and negative variant positions, WES kit interrogation regions and their overlaps, high-confidence regions, CTR) from this study is provided with a representative gene (*TP53*) in Fig. 4.

To maximize the number of variants with a wide range of VAF, we selected several diverse cell lines derived from distant individuals. These cell lines represented a variety of cancer tissues having been previously selected to compose the Agilent UHR RNA [14]. Further, we used a very well-characterized cell line from a normal individual (Sample B) to dilute the VAF for the majority of variants in reference Sample A to create reference samples C(1:1), D(1:4), and E(1:24). However, one could create a different reference sample using other cell lines. For more detailed recommendations and information related to creating a reference sample with low allele frequency variants, including the impact of the number of cell lines to admix, see Additional file 3: Supplementary Information section “Recommended process for reference sample creation.”

### Characteristics of reference Sample A and the cell lines than comprise it

The cell lines examined and pooled for reference Sample A are diverse in several ways. In addition to being reflective of different tissues of origin, they also are reflective of some degree of genetic diversity as they contain germline variants from 10 distinct individuals. In fact, most variants identified are germline variants as cancer cells typically have less than 10 mutations per Mb of coding sequence while typical germline variants may be on the order of hundreds per Mb of coding sequence. The number of variants positively identified for each cell line (including Sample B) is provided in Additional file 1: Table S8.

Population ancestry and the number of somatic variants are the primary cause of variation in magnitudes of SNVs and Indels across cell lines. The TLY cell line itself has roughly 19k variants (including by far the most identified somatic alterations, possibly numbering over 6000). In addition to TLY having many somatic variants, TLY is also essentially tetraploid with some variations (Additional file 2: Fig. S3). Other characteristics of the cancer cell lines include a high degree of structural variation. Some cell lines have more than 50% of their genome impacted by somatic copy number alterations (sCNA) while all are estimated to have at least 22% of their genome impacted by sCNA (Table 1). Two cell lines, BRA and LIP, have apparently greater than 90% of their genome impacted by sCNA. In addition to structural variation, there are also polyclonal aspects to some cell lines. For example, BRA, BRE, and LIP cell lines are considerably polyclonal while LIV, MAC, and TES are much less so. Values of intra-tumoral heterogeneity [35] that support this assessment are also provided in Table 1.

Finally, we cross-referenced the 42,570 Sample A positives with ClinVar and Hotspots databases to see how many variants were potentially pathogenic and thus of great consequence. From Additional file 1: Table S9, we see that Sample A contains at least 58 pathogenic variants (50 from ClinVar and 13 from Hotspots). Some of these variants are well known such as the BRAF V600E variant and the NRAS G12C variant.

### Variant allele frequencies of the individual Universal Human Reference cell lines and the resulting pooled Sample A

**Fig. 5:**
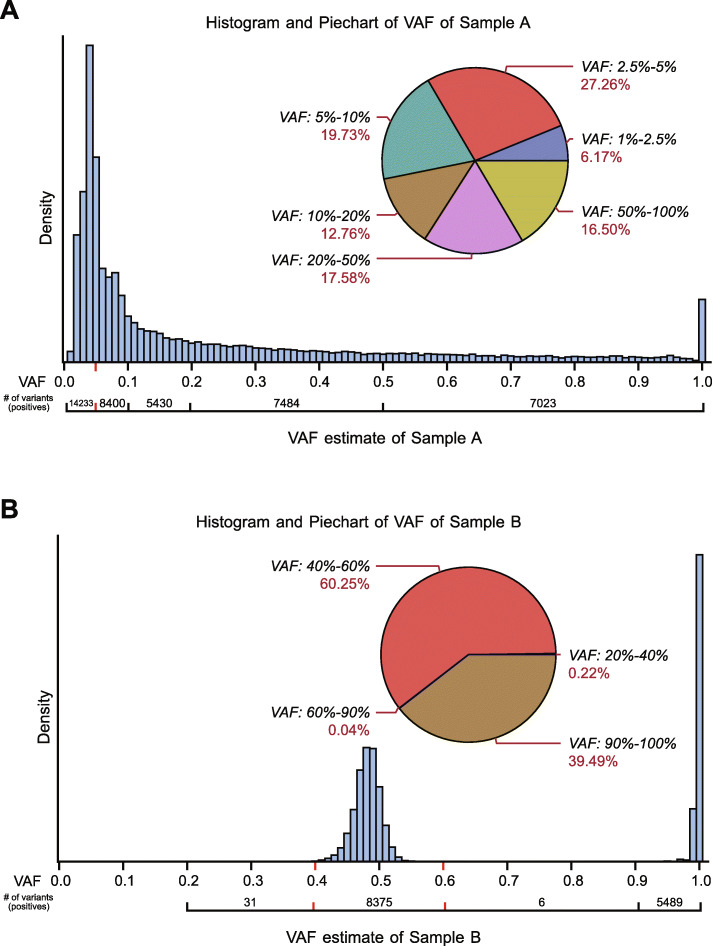
**a** VAF histogram of Sample A variants (Class 1 and Class2) with the obvious large numbers of variants in the low VAF range from 0.01 to 0.10. **b** VAF histogram of normal Sample B which can be used to dilute variants from Sample A

VAF values of the identified variants within each cell line are an important characteristic and play a primary role alongside of variant commonality between cell lines in determining the resulting VAF in the pooled Sample A. Of tertiary importance is whether a particular variant in a cell line is in a region having large copy number deviations from the normal diploid state. In theory, unshared variants in individual cell lines that are heterozygous should result in a VAF of approximately 5% in pooled Sample A while homozygous unshared (between cell lines) variants should result in a VAF of approximately 10% in A. From Fig. 5a, the observed VAF from pooled Sample A is in the range of 0 to 20% where ~ 70% of the Class 1 and Class 2 variants are observed.

From the histogram, there is a notable overall mode around 5% but no local mode around 10%. The likely reason for the lack of a local mode at 10% is that any germline variant that is homozygous in one cell line is most likely a high frequency population variant and therefore would likely be present in other members of the same population, causing the resulting VAF in a pooled sample like Sample A to be greater than 10% depending on the variant’s population frequency. The VAF distribution of normal Sample B (Fig. 5b) reflects the hetero-homozygous dynamic that we expect as well as a nearly total deficiency of variants that have VAF up to 40% or are between 60 and 95% due to the (as expected) near absence of somatic point mutations and copy number variants. While Fig. 5a provides the VAF distribution of all variants (Class 1 and Class 2), they are dominated by the presence of Class 1 variants (> 99%). Additional file 2: Fig. S4 shows the distribution of the Class 2 variants in Sample A. Although the distributions are similar, the Class 2 variants show a slightly smaller percentage of their variants having VAF <  20% in Sample A (58% of all Class 2 variants vs. 66% overall). The histograms of VAF of each individual cell line that constitutes Sample A are provided in Additional file 2: Fig. S5a-j. One can see evidence of CNA in the VAF histograms for most cell lines. Similarly, histograms of VAF of each individual cell line for Class 2 variants in Sample A are provided in Additional file 2: Fig. S5k-t.

We investigated the potential amount of bias in individual VAF estimates that may be associated with each kit and may be a unique characteristic with certain variants. Bias in VAF can arise from several factors: bait-hybridization bias, mapping errors and bias in mapping towards the reference genome [36], and confusion in calling a multiple nucleotide polymorphism or a complex multi-allelic polymorphism. To separate mapping and calling issues from bait biases, we ran several individual pipelines including a compendium method (SomaticSeq) for each kit of pooled Sample A replicates as well as in silico Sample A and merged-BAM Sample A (see “Methods” for details). In silico Sample A for each enrichment kit was based on equal overall read depth by cell line of BAM files accumulated across Sample A’s components and then calling variants based on the 20× larger BAM (as each cell line had two replicate library results). Merged-BAM Sample A was a merging of the three independent library sequencing BAMs (by kit) and then calling variants on the roughly 3× larger BAM relative to an individual library BAM. In each case, either result helped overcome sampling variation and other variation (such as variation in CN state across cell lines) to provide a less variable VAF estimate for pooled Sample A. Additional file 2: Fig. S6 is a plot matrix that illustrates the high level of concordance that the raw Sample A VAF average, in silico Sample A and merged-BAM Sample A VAF estimates have with each other as well as across WES kits. The correlation between and among kits for Sample A is equal to or exceeds 0.996 in all cases with the variation around the expected 45-degree line in the range of 0.05–0.10. We also observed a similar consistency across kits with correlation exceeding 0.987 between any two distinct kits whether cell line averages, in silico or merged-BAM results. For final VAF values for binning purposes to evaluate panel performance, we used merged-BAM estimates averaged across the kits (see “Methods” for more details). From Fig. S6, little differential bias appeared in VAF estimates between WES kits. However, there are most likely common biases that exist for most capture-bait enrichment techniques overall (see more details in the section Additional file 3: Bias in reported WES allele frequencies).

In addition to concordance in VAF estimates across kits, we also observed general concordance between kits in straightforward detection of the variants of Sample A using only one bioinformatic pipeline: SomaticSeq. Additional file 2: Fig. S7 illustrates the agreement among kits among variants in the CTR whether one examines the Merged-BAM Sample A construct or the in silico Sample A from Fig. 1. The vast majority (96%) of positives were detected in all three WES1-WES3 kits in both merged-BAM and in silico Sample A using the single pipeline. When we examined the potential reasons for variants identified in only one or two kits, we observed that undetected variants were typically from lower-depth libraries or lower-depth ROIs from the library. Therefore, our conjecture is that one can identify the great majority (> 90%) and estimate the total number of potential positives for a particular candidate reference sample using one kit with three independent library replicates of the pooled sample with sufficient depth combined with an advanced variant caller such as SomaticSeq.

We examined the variant and variant allele distributions by chromosome, by type (Indel vs. SNV) and by VAF range. Additional file 1: Table S10 provides a general view of the frequencies of identified indels vs. SNVs by chromosome and of how many variants by chromosome are in different VAF ranges: 1–2.5%, 2.5–5%, 5–10%, 10–20%, 20–50%, 50–100%. Some unusual characteristics of the cell lines that we perceived from the table include the large number of low-frequency variants seen for chr11 and chr16 compared to peers and the inordinately high number of variants in the 2.5–5% range for chr19 in the TLY cell line.

Examination of data from individual cell lines allowed for identification of potential issues such as cell line contamination, pooling errors, or pooling imbalances. In fact, in the initial creation of DNA for cell line BLY, TLY was inadvertently admixed with BLY in a 2:1 M ratio prior to the creation of pooled sample A and the admixtures C, D, and E. The consortium recognized this admixture early on when analyzing the individual cell line WES results. For example, we noticed the following relationships between the TLY and BLY DNA samples received:

- The major copy number alterations (CNA) of TLY were also observed in the BLY mixture (Additional file 2: Fig. S8)
- 95% of the detected variants including more than 1000 somatic variants in TLY were also detected in the BLY mixture. We did not observe 100% detection as TLY had many low allele frequency variants that, when diluted into BLY DNA, enabled them to elude detection at study-related sequencing depths of individual cell lines.

This admixing implied that regions from the DNA initially labeled as BLY and having variants unique to the original cell line of BLY would be underrepresented in the pooled Sample A. Similarly, variant regions for variants unique to TLY would be overrepresented, implying that variants unique to BLY and TLY would not necessarily have a VAF in the pool equal to roughly 1/10 their frequency in the individual cell line. However, due to sometimes large CN alterations in other cell lines, this property was not unique to BLY and TLY. In the end, we determined that the pooled reference (Sample A) itself provided the best estimate of the pooled VAF through direct interrogation as the pool naturally subsumes positional-dependent depth variation across cell lines. As the final estimates of VAF for Sample A are based on the direct testing of Sample A itself, the inadvertent admixing of BLY and TLY when creating DNA for the BLY cell line had minimal final impact.

## Discussion

One primary challenge in oncopanel validation is to have appropriate reference samples to gauge the true performance characteristics of a test. This evaluation must often cover various aspects of performance that are beyond simple summaries of binary data (detected/not detected). Panels themselves can range from testing a few thousand loci to tens of millions. This variety creates complexity related to characterizing assay performance as the LOD for a given analyte may be variable based on the locus and variant type. For example, it is well known that SNVs are easier to detect (higher recall/sensitivity) than small indels, which in turn are easier to detect than larger indels regardless of the VAF. Moreover, for each variant type, generally those variants having higher VAF are easier to detect than those with smaller VAF. To address these requirements, organizations have often historically used bespoke mixtures of their own in-house or artificially constructed specimens combined with limited publicly available reference samples to develop, evaluate, and validate their DNA assays. However, the custom nature of these samples outside of typical GMP-level manufacturing leads to questions regarding reliability, bias, and stringency of the sample and the testing. We believe that a proper reference sample from a vendor who has proficiency with building these samples will improve the quality and trustworthiness of oncopanel testing in general.

We recognized early in the process that the reproducibility of identified variants between pipelines for the same initial FASTQ data sets were greatly improved if we focused on high-confidence regions of the genomes. These high-confidence regions, also known as benchmark regions, were determined by NIST efforts in sequencing the Genome-In-A-Bottle (GIAB) germline reference specimens, which were some of the first reference samples for validating methods when detecting germline or population variants. Additional file 2: Fig. S9 illustrates the unexpected VAF of a large percentage of putative variants in Sample B that replicate in a distinct run when using positions outside of high-confidence regions. As Sample B is from normal tissue, there should be minimal loci influenced by CN or somatic changes creating loci with VAF in ranges outside of VAF = .5 and VAF = 1. When restricted to high-confidence regions like the CTR, aberrant variants can constitute less than 1% of the total (whereas unrestricted they may constitute more than 20% of the total). Therefore, we see that high-confidence regions have a greatly reduced likelihood of false positive variant calls. Lower confidence regions typically have one of the following characteristics: short repeat motifs, general low diversity or base complexity, or sequence similarity with other regions of the genome. Calling variants with high accuracy and specificity in these more challenging regions may require more than short-read technology. The technologies utilized in our study were based on short reads as a primary backbone to achieve the required depth for low allele frequency with reasonable cost. More importantly, short-read technology is inherently compatible with liquid biopsy specimens which is characteristically composed of small DNA fragments (~ 150b). Therefore, we restricted our domain for the initial positive and negative variant set for Sample A to be within the high-confidence human exome that was not of low complexity.

While the consortium’s efforts have identified a reference sample with an unprecedented number of known positives in coding regions as well millions of negative loci in one reference sample, the positive and negative set is not comprehensive and can be enlarged in a variety of ways. For example, we did not examine introns and UTR regions of genes even though a high percentage of the ROIs for various enrichment kits have baits targeting these regions. There are well-known examples of important intron and UTR variants, especially those variants impacting RNA editing or splicing [37]. One can also extend the identified variants in Sample A in COSMIC genes (and in other genes) by possibly 50% by mining the lower confidence coding regions (high-confidence regions are 63% of the bases of the human exome). However, one may need to utilize longer-read technology at high depth with mostly intact DNA. We also initially omitted examination of chromosome X and Y as mutations in these regions are frequently more associated with developmental disorders and other non-cancerous diseases even though some chrX genes are associated with cancer [38].

Some classes of variants are most likely underrepresented. In particular, the proportion of identified indels to variants is only 1.3%, although this proportion is higher in COSMIC genes, especially at low VAF. The SEQC2 Somatic Mutation Working Group observed a 3× larger rate for indels [39] (4%) in their detailed analysis of a one breast cancer cell line (HCC1395) and the GIAB consortium initially observed a 2.3% rate for indels in NA12878 (a normal cell line) in the high-confidence regions of NA12878 (also known as HG001). Still others have observed a much higher indel rate in whole exome and whole genome analysis for other specimens (e.g., 12% [40], 13% [41]). However, it is unclear how these ratios may differ considering high-confidence regions only. Although we have identified more than 500 indels in Sample A, we recommend the Sample A reference be further investigated specifically for relevant indels, such as those in COSMIC genes. Although relatively small in magnitude, indels are excellent phenomena for challenging various mapping and variant calling methods. Therefore, expanding the known indels in Sample A will provide a potentially less optimistically biased assessment of the capability of panel assays in detecting indels in general.

Using hg19, the consortium identified 42,570 unique variants in pooled Sample A in coding regions and 2432 (or 5% of the total) in COSMIC [42] Tier 1 and Tier 2 genes. There are roughly 723 COSMIC Tier1 and Tier2 genes which is 3.4% of the total number of coding genes. The enrichment of identified variants in COSMIC genes is partly due to our focus in finding Class 2 variants in COSMIC genes outside of the Interval4 region. In that effort, we identified 359 additional variants in COSMIC genes relative to our initial Class 1 positive set. The Class 1 positives include 2073 variants from COSMIC genes which are 4.9% of the total of 42,211 variants in the Class 1 set. However, the probable primary reason for COSMIC genes being enriched for variants is due to selection bias in our reference sample (ten distinct cancer cell lines versus normal cells).

The Sample A variant content compares favorably in magnitude and is complementary to other reference samples. As described previously, Table 2 provides a comparison of the identified contents of Sample A versus Acrometrix and OncoSpan reference samples by VAF range and by gene category (for COSMIC genes). For example, Sample A contains at least one variant in 422 COSMIC Tier 1 genes, with 375 COSMIC Tier 1 genes having a variant at low frequency. By comparison, OncoSpan’s comparable values are 114 and 29 COSMIC Tier 1 genes respectively and Acrometrix’ comparable values are 52 and 37 COSMIC Tier 1 genes respectfully (Note: Acrometrix can manipulate their synthetic variants to be at lower VAF, if needed). Acrometrix concentrates their variants into specific genes. For example, 145 of the 555 identified variants in Acrometrix are in four genes: TP53, PTEN, EGFR, and APC. However, only 42 of those 145 variants have a VAF less than 20% and all 42 variants with low VAF are in TP53 only. That is, no variant in the standard Acrometrix controls for PTEN, EGFR, and APC has a VAF less than 20% even though these three genes contain almost 20% of all identified variants. In comparison, for the same genes, Sample A has less variants overall in these four genes (31 vs. 145) than Acrometrix but over half of these Sample A variants have a VAF less than 20%. Also, in Sample A, each of TP53, PTEN, EGFR, and APC have at least one variant with a VAF less than 20%. OncoSpan’s identified variants are more uniformly distributed over the OncoSpan gene set. However, OncoSpan’s relative concentration of variants is much lower than Sample A (0.2 variants per COSMIC Tier 1 gene for OncoSpan vs. 3.1 variants for Sample A). In short, all of these reference samples are useful when developing and validating panels. Acrometrix in particular with its high proportion of variants concentrated in relatively few genes would be highly suited for small fragment-based panels that overlap with its concentrated list (amplicon-based panels may have challenges with the high concentration creating interference with primers). OncoSpan and Sample A test variants in many more important COSMIC genes. Sample A also tests variants throughout the human exome, averaging two variants for every gene and having 12,238 genes with at least one identified variant. Acrometrix and OncoSpan contents are focused on currently known actionable variants. Sample A content, while containing many important and actionable variants, is more designed for assessing analytical performance across a flexible target region for a wide variety of potential genomic assays including clinical assays.

During the initial phase of discovering variants in Sample A, we examined data from four different whole exome enrichment kits. When reviewing the sequenced data results, we realized that the data from the AmpliSeq WES4 panel was noticeably different than the other three WES panels. In particular, the other panels tested technical replicates for each cell lines while the WES4 results had only one singlet per cell line. The WES4 results also had lower coverage per cell line which presented challenges in detecting lower frequency variants in certain cell lines, especially the TLY and BLY cell lines that had a higher concentration of variants at lower VAF values. We also had many fewer pipeline outputs with WES4. Therefore, the WES4 results were used as an independent confirmation of the positives derived from WES1-3 and WGS1 which all used Illumina-based sequencing. We were successful in that 97.8% of the Sample A positives having a VAF greater than 5% were detected by WES4. The gap is primarily due to the lower depth of WES4 especially for BLY. However, we encourage the community to evaluate Sample A with other alternative sequencing technologies so that omissions can be uncovered and any potential false positives, especially variants identified at the lower allele frequencies (less than 2%), can be identified.

The consortium made extensive use of the v3.3.2 NIST high confidence (benchmark) regions from the ongoing GIAB project [2, 43, 44]. We examined the high-confidence regions from five individuals: the female Caucasian (HG001), the Ashkenazim trio (HG002-HG004), and the Chinese son (HG005) within a trio [2]. NIST has publicly provided well-annotated and defined high-confidence benchmark regions for detecting small variants (SNVs and small Indels) for both hg19 and hg38 versions for their entire genomes. We observed that the high-confidence regions for HG002-HG005 had greater than 95% overlap (min X ∩ Y greater than 95% for X and Y ε HG002-HG005) for the coding regions of the human genome. However, the high-confidence region for HG001 was noticeably different than the other four in terms of size: HG001 benchmark regions were noticeably larger by 3–4% (even after excluding chrX regions exclusive to HG001). Reasons for these differences are discussed in the section “NIST high-confidence regions (v3.3.2 benchmarking regions)” in “Methods.”

We chose a more conservative approach for ground truth and created a common high-confidence region based on overlapping regions of HG002, HG003, HG004, and HG005 to serve as the basis for high-confidence regions for this initial release of positives and negatives. In addition, we excluded a small amount of low complexity regions [32] overlapping with this more universal high-confidence region which served as the basis for both Class1 and Class 2 variant detection. Although these benchmark sets were developed for NIST sample-specific purposes, the consortium determined early on that the reproducibility of variant calls in general was much greater within these benchmark regions than outside. As we utilized the v3 NIST benchmark regions for this release, it is more suited for use with panels employing shorter-read technologies. As v4 benchmarks become available, this Sample A reference should be updated to be more compatible with long-read methods [45].

From ddPCR analysis, we detected a small reference bias in the WES results. This bias was largest in the middle of the dynamic range of variants measured in Sample A [.01,1] and generally larger for indels than SNVs. We typically observed SNV VAF values that were up to 7% relatively higher for ddPCR than that observed from the consensus WES estimate. Indels could be as much as 15% relatively higher for ddPCR than the WES estimate. For example, SNVs and indels observed at 10% VAF from WES would typically be observed at 10.2 to 10.7% and 10.5 to 11.5% respectively from ddPCR. Although this bias is rather small, it may have certain impacts for thresholding, even at lower VAF thresholds when comparing results from orthogonal measurement platforms. The bias can be most easily observed in Fig. 3a where biases in the indels are clearly observable to the left relative to the other types of variants (especially near VAF = .5 and 1). See Additional file 3: Supplementary information section “Bias in reported WES allele frequencies” for more details.

When using a reference sample such as Sample A for cancer panel or liquid biopsy performance evaluation as in companion efforts by the SEQC2 consortium [46, 47], caution must be taken when performing the final evaluation. A large portion of the low-frequency variants in Sample A originated as germline variants in the original cell line. As some pipelines may automatically remove certain known germline variants when identifying somatic changes, one may need to review filtered results to properly account for putative false negatives. Otherwise, key metrics such as recall/sensitivity and precision may be greatly impacted. Likewise, complex variants such as multi-allelic variants and multi-nucleotide variants (MNVs) can pose challenges in both representation and proper assessment of performance of solid tumor and liquid biopsy panels. Reference Sample A contains both multi-allelic variants and MNVs. See the section “Complex variants including multi-allelic variants and multi-nucleotide variants (MNVs)” in “Methods” for a more detailed discussion of these variant types in Sample A and techniques that properly characterize them.

In addition, we implemented certain constraints to increase confidence in our negative positions as proving a negative is inherently difficult. Those constraints may omit regions that are subject to systematic errors in targeted sequencing thereby potentially causing an underestimate in the true false positive rate when using Sample A alone and its identified negatives. Additional orthogonal sequencing technologies can potentially be used to further expand the set of negative variant loci of Sample A so that systematic errors related to a particular technology or genomic region are not hidden. Complementary alternative methods to estimating the false positive rate indirectly include assessing the rate of low VAF loci in known normal cells (something that should not exist). This method be unbiased relative to systematic sequencing errors that could have biased our negative set for Sample A.

Reference samples or reference DNA are ideally produced using a large pool of such DNA at one time so that aliquots will be a homogeneous and consistent resource for several years. This was done for this study and this key practice has important ramifications. The cancer cell lines that constitute Sample A already have chromosomal remodeling and significant copy number alterations (CNA). As the cell lines go through additional passages, more alterations and new variants could be reasonably expected. Therefore, each new iteration of the resulting large DNA reference sample pool should be thoroughly assayed to monitor and quantify changes, which will invariably occur given the magnitude of relevant variants having low VAF in the reference Sample A. Due to the different ploidies that occur within and between each cancer cell line, the pooled Sample A is not as suitable a reference sample for detecting CN alterations, even at the ploidy level. As we do not propose Sample A for this purpose, given the creation of a large pool of DNA with verified variants, the main impact of variations in ploidy levels are corresponding variations in observed depth levels of Sample A. However, the “natural” variation in the resulting library due to enrichment bait bias impacts coverage depths in the final pool more noticeably in general than ploidy-level changes in individual cell lines. Therefore, ploidy variation in the individual cell lines create no worse variation in overall read depths in Sample A than what is already occurring due to the nature of the bait-based WES assays (based on comparing the distribution of read depths in variants common between the normal Sample B which only has bait bias impacting local depth and Sample A which has both bait bias and ploidy variation—data not shown). So long as new iterations of the cell lines and Sample A are properly tested, we see no issue in updating the variant information for each iteration.

## Conclusions

We have reported efforts by the SEQC2 consortium Onco-panel Sequencing Working Group to address urgent needs in the regulatory science and precision medicine communities regarding genomics reference samples. NGS is rapidly changing clinical care in oncology, drug development, and regulatory science. The 21st Century Cures Act sought to catalyze the development of new medical technologies when fundamental advances in our understandings of the genetic basis of disease and advances in medical technology are allowing significant progress on previously vexing conditions.

The two reference samples (Sample A and B) described in this document have an unprecedented number of known positive variants and known negative loci down to a 1% VAF. In particular, we have identified 42,570 distinct positive variants and greater than 10,000,000 negative loci in human exome coding regions. In reference Sample A, we have identified 1653 variants in COSMIC genes with VAF between 1 and 20%, implying on average more than two positive low allele frequency variants per COSMIC gene. Sample A has, to our knowledge, the largest number of identified positive variants in COSMIC genes: ~ 5-fold as many as the Acrometrix control, ~ 40-fold as much as OncoSpan, 100-fold the amount in HCC1395) having low variant allele frequency values [1–20%] for one reference specimen.

When combined with the normal Sample B reference genomic background or similar, one can create a new reference sample that has ever decreasing lower ranges of VAF for validating highly sensitive cancer panels or liquid biopsy assays. The Sample A and B content verification utilized four different WES library prep methods having very high levels of concordance. The study also used WGS to enrich known positives in COSMIC genes which were not in common ROIs to all kits. The methods and results were verified with ddPCR across a wide range of variant characteristics (low VAF, difficult indels, random SNVs, and VAF influenced by CNA).

Initially, the consortium utilized a wide range of bioinformatic methods to ascertain the relevant content of the candidate reference samples. We subsequently determined that the consortium-wide bioinformatic effort could be greatly reproduced by implementing a single high-quality pipeline (such as SomaticSeq) with fewer (two or three) enrichment kits, but requiring duplicate libraries of sufficient quality, complexity, and depth. Similarly, duplicate libraries from a small number of enrichment kits can monitor any drift in the cell lines and reference sample to provide quality assurance for future batches.

## Methods

### Library preparation and sequencing of cell lines and Sample A with WES1 (Roche) and WES2 (IDT) exome kits performed at University of Texas Southwestern Medical Center and Novogene

All genomic DNA samples were provided by Agilent (Agilent Technologies). Whole exome sequencing libraries were constructed using KAPA Hyper Prep kit (Kapa Biosystems) and Roche NimbleGen SeqCap EZ hybridization and wash kit (Roche Sequencing Solutions), or Next Ultra II DNA Library Prep kit for Illumina (New England Biolabs) and IDT xGen hybridization and wash kit (Integrated DNA technologies, Inc.) according to the manufacturers’ instructions. Briefly, genomic DNA was sheared to an average fragment size of 200 bp or 300 bp on Covaris S220 (Covaris). Ten nanograms, for 10 UHR cell lines and Sample B in duplicate, or 100 ng for Sample A in triplicate, of fragmented DNA, was used as input for the library preparation. Samples were sequentially end-repaired, A-tailed, and adapter-ligated. The libraries were then subjected to minimal PCR cycling and quantified with Agilent DNA 1000 assay. One microgram of each sample library was hybridized with WES1 and 500 ng of each sample library with WES2. The hybridized probe-target complexes were captured with streptavidin beads and washed to remove non-targeted DNA. Captured libraries were amplified by PCR, and the quality of the libraries was validated by Agilent high sensitivity DNA assay and quantitative PCR. The libraries from each panel were pooled in equimolar amounts and subjected to 150-bppaired-end sequencing (PE150) at Novogene on an Illumina HiSeq 4000 for the cell line libraries and on an Illumina HiSeq X Ten for Sample A libraries.

### Library preparation and sequencing of cell lines and Sample A with WES3 (Agilent SureSelect) exome kits performed at Q^2^ Solutions/EA Genomics and Novogene

Genomic DNA libraries from individual cell lines blended in Sample A and the final pooled sample were constructed in duplicate for individual cell lines and in triplicate for Sample A according to the SureSelectXT Target Enrichment System for Illumina Paired-End Multiplexed Sequencing workflow at EA Genomics with additional automation protocol modifications as described elsewhere [48]. In brief, 200 ng of each cell line high molecular weight genomic DNA was sonicated in a 50 μl total volume in a Covaris E220 instrument to a mean size of 150 bp (Duty Factor: 10%, Peak Incident Power: 175, Cycles per Burst: 200, Treatment Time: 2 × 180 s, Bath Temperature: 2° to 8 °C). DNA fragments were then end-repaired and A-tailed, followed by ligation to XT adaptors for 15 min at 20 °C. Adapter-ligated fragments were amplified by PCR in a 50 μl total volume with Herculase II Fusion DNA Polymerase under the following conditions: 2 min at 98 °C (initial denaturation), 10 cycle amplification of 30 s at 98 °C, 30 s at 65 °C, 1 min at 72 °C, and 10 min at 72 °C (final extension). Library quality control (concentration and size distribution) was then assessed using a Picogreen assay and the 2200 TapeStation with D1000 screen tape. In total, 750 ng of prepared gDNA libraries was then hybridized to SureSelect Human All Exon V6 biotinylated RNA probes for 24 h at 65 °C and captured with Dynabeads MyOne Streptavidin T1 beads. SureSelect enriched gDNA libraries were PCR amplified and indexed using on-bead protocol in a 50 μl total volume with Herculase II Fusion DNA Polymerase under the following conditions: 2 min at 98 °C (initial denaturation), 11 cycles of 30 s at 98 °C, 30 s at 57 °C, 1 min at 72 °C (amplification), and 10 min at 72 °C (final extension), followed by 4 °C hold. All DNA purifications between steps were performed with AMPure XP beads as indicated in the user manual. Post-capture library quantification was done using qPCR and fragment size distribution determined by HSD1000 screen tape assay on TapeStation 2200. Indexed libraries were finally pooled and sequenced on either an Illumina HiSeq 2500 or HiSeq X Ten instrument. Individual cell lines were sequenced at EA Genomics to a 200× on-target mean read depth after deduplication on HiSeq 2500 instruments in high-output mode with V4 sequencing reagents using a 2 × 100 bp paired-end protocol (Q30 scores ≥ 80%). Pooled Sample A libraries with 200 ng of input were sequenced at Novogene on a HiSeq X Ten-PE150 with standard workflow (post-deduplication target depth of 270×, 417×, and 583× using a 2 × 150bp paired-end protocol (Q30 scores ≥ 75%).

### Library preparation and sequencing of cell lines with WES4 (Thermo Fisher AmpliSeq) kits performed at Thermo Fisher

The Ion AmpliSeq™ Exome Panel [49] was utilized to generate libraries for next-generation sequencing on the Ion Torrent S5 platform. The panel contains 293,903 amplicons in 12 pools. This assay enables analysis of variants across > 97% of the Consensus Coding Sequences (CCDS). Eleven DNA samples (10 human cell lines and one human reference gDNA) were used to prepare libraries with a mean insert size of 215 bp. Exome libraries were generated following the manufacturer’s instructions in the Ion AmpliSeq Exome RDY Library Preparation User Guide [50] with 100 ng input for each sample. Each sample was assigned a distinct IonCode barcode [51]. Each barcoded library was diluted to 30 pM for template preparation on the Ion Chef™ Instrument using the Ion 540™ Kit-Chef [52]. Sequencing was performed with the Ion S5™ XL System [53] and the Ion 540™ Chip [54].

Signal processing and base calling were performed using Torrent Suite Software v5.4 using default parameters for the AmpliSeq Exome assay. The signal processing step consists of modeling the pH dynamics on the semiconductor surface taking account of the varying local pH in each individual sensor coming from the different reagent flows across the chip and from any nucleotide incorporation that may be happening over each sensor [55]. The base calling step consists of taking the estimated levels of nucleotide incorporation for each read and each nucleotide flow and modeling the de-phasing process whereby some templates within each clonally amplified population run ahead or behind in terms of their nucleotide incorporation. During the base calling process, sample-specific barcodes and 3′ adapters are annotated.

### Library preparation and sequencing of WGS1 with 10X Genomics performed at Cornell University and data analysis at National Heart Lung and Blood Institute

Samples were shipped on dry ice to the HudsonAlpha Institute. Sample aliquots were profiled for QC on a 0.4% agarose gel with ErBr, run at 58 V for 1.75 h. Libraries of each sample were synthesized using the 10X Genomics Chromium Genome kit according to the manufacturer protocol. Each library was sequenced on one lane of an Illumina HiSeqX. Raw sequence data was demultiplexed and converted to barcode and read data FASTQ files using 10X Genomics Long Ranger mkfastq version 2.2.1. Alignment, deduplication, filtering, and subsequent calling and phasing of SNPs, indels, and structural variants was achieved for each sample using Long Ranger wgs version 2.2.2, against both hg19 and GRCh38 reference human genomes retrieved from the 10X Genomics Long Ranger downloader website. Both GATK and FreeBayes variant callers were employed using Long Ranger alignments for WGS variant analysis.

### Agilent analysis of WES3

Each sample was demultiplexed using bcl2fastq with the base mask Y150, I8, Y10, Y150, and all default settings except for mask-short-adapter-reads, which was set to 0. Adapters were trimmed using AGeNT Trimmer (Agilent). All data was aligned to the hg19 reference genome using bwa-mem [29] v1.7.10 with default settings. Quality control was performed using Picard tools and an internally developed pipeline. Deduplication was performed with Picard [56] MarkDuplicatesWithMateCigar v2.9.2 with default settings except for a minimum distance of 500.

Variant calls were done as follows. SureCall v3.5.1.46 (Agilent) was run starting from aligned files with default settings with two exceptions: the ‘minimum number of reads supporting variant allele’ was set to 3 and the ‘minimum allele frequency’ value was reduced to .001. Samtools calls were completed using samtools [24] v1.3.1 and bcftools [57] v1.3.1, with commands of the form ‘samtools mpileup -d 8000 -uvf $REF_FASTA $BAM_FILE | bcftools call -mv | bcftools view -O v’. Platypus calls were completed using platypus [23] v0.8.1 and using default settings. GATK [58] Unified Genotyper v2.2–3 were run with default settings, except the filter filterMBQ was applied and dbSNP v147 was used as a reference.

### Roche analysis of WES1, WES2, and WES3

The FASTQ files from the WES1 dataset are quality checked with FastQC [59] to ensure the sequencing quality. Adapters were trimmed using AlienTrimmer [60]. The reads were then mapped to reference genome hg38 and hg19 with bwa-mem [29] (v0.7.12 (RSSMSN01-RSSMSN04)). Samtools [24] fixmate tool was applied to correct any read-pairing issues that may be introduced by bwa. Duplicated reads were marked with Picard [56] MarkDuplicates, and base quality score was recalibrated with GATK [58] BaseRecalibrator and ApplyBQSR. The processed BAM files were then supplied to four variant callers, i.e., GATK mutect2 (v2.1-beta/v 4.0 alpha), Vardict (v1.5.1), samtools mpileup (v1.2), and Sentieon TNscope [25] (201,704.03).

For the WES1, WES2 and WES3 datasets run with the RSSMSN05 and RSSMSN06 pipelines, a similar analysis was performed, but the mapping step to hg38 was done with bwa-mem [29] (v0.7.17). The processed BAM files were supplied to GATK [58] mutect2 (v 4.0.6.0) using default settings and GATK HaplotypeCaller (v 4.0.6.0) for variant calling.

### National Center for Toxicological Research (NCTR) analysis of WES1, WES2, and WES3

The NCTR team’s pipelines were applied on WES1, WES2, and WES3 datasets. Reference genomes were downloaded from Illumina iGenomes website for both hg19 and hg38 version. Reads were aligned with BWA-MEM [29] (v0.7.12-r1039) and Bowtie2 [31] (v2.3.2). Duplicate reads were marked using Picard [56] (v2.7.1) MarkDuplicates function. The reads were then local realigned around small insertions and deletions (indels) and base quality scores were recalibrated using GATK [58] (v3.6-0-g89b7209). dbSNP (b150) was supplied to GATK for indel realignment and quality score recalibration. GATK’s default downsampling option was suspended by setting “--downsampling_type NONE.” Variants were called with FreeBayes [21] (v1.1.0-46-g8d2b3a0) and VarScan2 [61] (v2.4.0) with their default settings.

### National Institute for Environmental Health Sciences (NIEHS) analysis of WES1, WES2, and WES3

The NIEHS team’s pipeline was applied to WES1, WES2, and WES3 datasets. Raw data from the FASTQ files was preprocessed with cutadapt [62] (v. 1.12) by removing the sequencing adapter and low quality read (Q20), then was aligned to human reference genome (hg19) with BWA-MEM [29] (v0.7.15-r1140) with default parameters (bwa mem -M -t 4 -a -V -T 60 -p). The alignment was post-processed with Picard [56] (v2.9.1) by removing the duplication, then bam files for each tissue were merged (by exome capture vendor platform library). All three batches of whole exome sequencing alignments have gone through an in-houseEnsemble-Variant-Calling pipeline, which contains Samtools [24] (v1.3.1.), Mutect1 [22] (v1.1.4), and VarScan2 [61] (v2.4.3), and the calling was done with default recommended parameters. Whenever the reference genome was needed for variant calling, hg19 was used for WES1, WES2, and WES3. Human SNPs obtained from dbSNP v.146 were used in the variant calling to mask germline variants. Somatic variants were reported according to each exome capture platform vender’s recommended filtering criteria and in conjunction with a minimum read depth of 20.

### Instituto de Genetica Medica y Molecular (INGEMM) analysis of WES1

The INGEMM team’s pipeline follows best practices of GATK version 3.3–0. First, the FASTQ files were preprocessed with trimommatic [63] v0.32. Then, the filtered sequences were mapped to the UCSC human reference genome hg19 (version February 2009) with Bowtie2 [31]. Duplicate reads were removed using Picard [56] RemoveDuplicated function. Indel realignment and base quality score recalibration was performed afterwards (RealignerTargetCreator and IndelRealigner functions from the suite GATK [58]). Variant calling was performed over the realigned and recalibrated BAM files. The variants characterized were the result of in-house consensus criteria between the outputs of the GATK variant callers UnifiedGenotyper and HaplotypeCaller. The consensus VCF files were filtered and annotated with Annovar [64]. In addition, the vcf file was enriched with prediction tools of pathogenicity provided by the proxy dbNSFP [65] (v3.0) together with population data (Exac Non-Finnish European data [66], clinical and genomic information).

The quality of library amplification and the sequencing procedure was assessed by a range of markers such as the percentage of mapped reads, the percentage of mapped reads in the region of interest (ROI), the percentage of duplicated reads, and the percentage of ROI over a depth of 20× (i.e., horizontal coverage). Also, the final efficiency of each sample was measured by the ratio of the sequences that are able for variant determination and the initial number of mapped reads.

Finally, the mean depth and the horizontal coverage together with a set of markers was evaluated to establish if the samples were suitable for the study. For that, each sample was flagged with any of the three statuses: Ok, warning, or rejection.

### Q^2^ solutions/EA genomics analysis of WES1, WES2, and WES3

WES1, WES2, and WES3 for individual cell lines were aligned with BWA-MEM [29] (v0.7.10) using hg19 reference sequence and local alignment was done with ABRA [67] (v0.94). Alignment was replicates for hg38 but for WES1 only. Deduplication was performed with Picard [56] (v1.140). Variants were called with VarDict [26] (v1.5.1) and Sentieon [68] TNscope (v201704) (http://www.sentieon.com/). Similarly, variants were called on Sample A using the same methods. Separately, WES3 cell line variants were also called for hg19 only with GATK HaplotypeCaller (v3.6–0). All results were output as VCF (v4.2). In addition, for some analyses, CNAs were called for WES3 with Covit (in-house tool) and CNV Radar [69] (in-house tool).

### Fondazione Bruno Kessler (FBK) analysis of WES2

Multiple lanes of the same replicate were merged into a single FASTQ file before read trimming with SeqPurge [70] (version 0.1-886-gf72a054) and alignment on hg19 with HISAT2 [71] (v2.0.4). At each analysis step, FastQC [59] (v0.11.5) was used to check for quality and processing progress. Variants were called using Platypus [23] (v0.8.1). We note that duplicate reads were not removed prior to variant calling, as Platypus directly deduplicates BAM files during the calling process. BCFtools [57] (v1.9-207-g2299ab6) was used for variant annotation, filtering and selection. tabix (v1.6) and RTGtools [72] (v3.8.4) were used to manipulate the VCF files and compute basic statistics.

### University of Fudan analysis of WES1, WES2, and WES3

Reference genome hg38 was used for all work. Reads were mapped with BWA [29] (v0.7.12-r1039). Variants were called with Sentieon Haplotyper (v201611.02). CNV estimation from WGS data was done with Breakdancer [73] (v1.1), CNVnator [74] (v0.3.3), Delly2 [75], DWAC-Seq(v0.7), GenomeSTRiP [76] (v2.0), Meerkat [77](v0.189), MetaSV [78] (v0.5.2), read depth (v0.9.8.4), svclassify [79], and Pindel [80] (v0.2.0). CNV estimation for the WES datasets was performed with CNVkit [81] (v0.8.5), CODEX [82] (v1.6.0), CopywriteR [83] (v2.6.0), DeAnnCNV [84], EXCAVATOR2 [85] (v1.1.2), ExomeDepth [86] (v1.1.10), GATK [58] 4 Alpha, RefCNV [87], SAAS-CNV [88] (v0.3.4), and VarScan2 [61] (v2.4.2). All results were output as VCF files.

### Thermo Fisher analysis of WES4

After completion of primary analysis with Torrent Suite [89] v5.4, reads were uploaded to Ion Reporter [90] v5.6 for subsequent processing. Reads were aligned with tmap [91], which uses the BWA fastmap routine to map reads and applies post-processing of the alignments to optimize for technology-specific error patterns. After alignment, variant calling was performed with Torrent Variant Caller (TVC) [92], a variant calling framework optimized for Ion Torrent data. TVC takes as input the aligned reads and uses a modified version of Freebayes to generate a very permissive list of candidate de novo alleles to be evaluated. The de novo alleles are evaluated in a statistical likelihood model that compares the observed flow signals for all of the aligned reads with the flow signals that would be expected under reference and non-reference hypotheses. The use of flow signals leads to significant improvements in variant calling compared to variant calling approaches that rely on base calls alone. At each position evaluated, the posterior likelihood of each evaluated allele’s frequency is assessed to determine if the null hypothesis that the allele frequency is less than or equal to a particular threshold can be rejected. Finally, a series of post-calling filters are applied to variant calls to filter out situations where the statistical model of flow signals is not a good fit for the observed data, and to eliminate potential artifacts where a variant appears on only one strand in regions with coverage on both strands, or in only one amplicon in regions where more than one amplicon spans the variant.

### CN analysis of individual cell lines

The CN analysis of individual cell lines to assess the level of chromosomal alterations individual cell lines was performed using the GenetiSure Cancer Research CHG + SNP microarray. Another method used WES3 VAF and relative depth profile data (WES3 had highest average depth of the WES kits) to illustrate certain ploidy-level characteristics of specific cell lines as shown in Additional file 2: Fig. S3.

The GenetiSure Cancer Research CGH + SNP Microarray, 2 × 400 K (Agilent, G5975A), used in this study contains approximately 300,000 CGH probes and 120,000 SNP probes. Many of CGH probes are targeted to cancer regions of the genome with median CGH probe spacing of 10 kb in the targeted cancer regions. CN changes (amplifications and deletions) are measured using three or more CGH probes for almost 90% of the covered exons, providing resolution down to the single exon level. Percent of CNAs in each individual UHR cell line was estimated using the CytoGenomics software v 4.0.

DNA samples were prepared using the SureTaq complete DNA labeling kit (Agilent, PN 5190-4240) as described in the Agilent Oligonucleotide Array-Based CGH for Genomic DNA Analysis Protocol (Version 7.5 June 2016). One microgram of each DNA sample was enzymatically digested and then labeled with Cy5 or Cy3 dyes. Ten individual UHR DNA samples labeled with Cy5 were hybridized against sex-matched reference samples labeled with Cy3. Samples were hybridized to the GenetiSure Cancer Research CGH + SNP Microarray, 2 × 400 K for 40 h at 67 °C, then washed, scanned using the SureScan scanner, and analyzed using the CytoGenomics software v 4.0.

### QC analysis of the Sample A individual libraries

FastQC [59] v0.11.6 was used to determine the quality of the demultiplexed FASTQ files. Each sample in each lane was downsampled to 120 million read pairs using SeqTK v1.0 and mapped using bwa-mem [29] v0.7.15 with default parameters. Reads from 5 different lanes were merged using Picard [56] v2.9.0 MergeSamFiles. To assess the library size and percent duplication, duplicates reads were identified with Picard v2.9.0 MarkDuplicates. Duplicate marked BAM files were used to evaluate target enrichment using Picard v2.9.0 HsMetrics.

### In silico Sample A methods (confirmation)

For each WES kit, we prepared the in silico Sample A by combining reads from all cell lines. For each cell line replicate, we aligned and marked the duplicated reads using Picard [56] MarkDuplicates. Then, we mixed the deduplicated alignments of all cell line replicates, followed by GATK [58] IndelRealigner and Base Quality Score Recalibration (BQSR) steps on the mixed alignment for the derived in silico Sample A. We then used SomaticSeq [28] in tumor-only mode to combine somatic mutation predictions from six individual somatic mutation callers, Strelka2 [93], MuTect2 [94], VarScan2 [61], VarDict [26], LoFreq [95], and Scalpel [96] on this sample. Scalpel only detects INDELs and the other five callers detect both SNVs and INDELs. Mutations with ≥ 3 supports were included in the final output. The VAF for the reported calls are computed using the minimum mapping quality of 1 and the minimum base quality of 5.

### Merged-BAM Sample A methods (confirmation)

For each WES kit, we prepared the merged-BAM Sample A by combining aligned reads from different libraries of pooled Sample A. Each pooled Sample A library was aligned and deduplicated separately using Picard [56] MarkDuplicates. Then, we mixed the deduplicated alignments, followed by GATK [58] IndelRealigner and BQSR steps on the mixed alignment to obtain the merged library A alignment. Using SomaticSeq [28] in tumor-only mode, we then combined predictions from six individual somatic mutation callers, Strelka2 [93], MuTect2 [94], VarScan2 [61], VarDict [26], LoFreq [95], and Scalpel [96] on this merged library. Scalpel only detects INDELs and the other five callers detect both SNVs and INDELs. Mutations with ≥ 3 supports were used for assessment. The VAF for the reported calls are computed using the minimum mapping quality of 1 and the minimum base quality of 5.

### NIST high-confidence regions (v3.3.2 benchmark regions)

As described in the main text, NIST has, through their Genome-in-a-bottle (GIAB) program, made available at ftp://ftp-trace.ncbi.nlm.nih.gov/giab/ftp/release/ variants and high-confidence genomic calling regions for (at the time) five distinct persons. High confidence for GIAB samples was achieved by several methods, of which the initial primary results were from determining consensus among distinct library preps (including 10X Chromium preps), sequencing, (e.g., sequencing platforms typically involved Illumina, Ion Torrent, and SOLID), and variant calling methods. The genome HG001 (aka NA12878), had additional data from more sequencing platforms and additional review to identify extended regions from the initial consensus for what was viewed as “high-confidence” calls for that genome. Due to this special case of additional data and review of the high-confidence regions, the SEQC2 consortium instead examined the high-confidence regions for HG002, HG003, HG004, and HG005 (all v3.3.2) and their overlap for hg19 and hg38, respectively. The overlap in high-confidence regions between any pair of this set was greater than 95% while the overlap in high confidence of any member of the set with HG001 was usually less than 91% of HG001 due to its much larger size and that HG001 includes chrX while the others did not. The final region selected as the high-confidence region for this study for the individual cell lines and their mixture was the intersection of the high-confidence regions of HG002-HG005. Although we performed parallel discovery and validation for hg19 and hg38, the positives are primarily determined using the high-confidence region of hg19 as hg38 is noticeably smaller in size.

### Human exome

Human genome annotation files were downloaded from Ensembl FTP site. For hg19, gtf file was downloaded on August 23, 2017 from ftp://ftp.ensembl.org/pub/release-75/gtf/homo_sapiens /Homo_sapiens.GRCh37.75.gtf.gz. For hg38, gff3 file was downloaded from ftp://ftp.ensembl.org/pub/ release-97/gtf/homo_sapiens/Homo_sapiens.GRCh38.97.gtf.gz on September 22, 2019. The exon regions of major chromosomes (chr1-22, chrX, chrY, and chrM) were extracted and saved as BED files for both versions accordingly.

### Human coding regions

Human coding regions were downloaded from UCSC. In UCSC Table Browser, we chose “Feb. 2009 (GRCh37/hg19)” for assembly, “Genes and Gene Predictions” for group, “NCBI RefSeq” for track, and “UCSC RefSeq (refGene)” for table. We chose “BED” as the output format and then clicked “get output”. We chose “Coding Exons” and clicked “get BED.” We performed the same procedure for hg38. The Coding BED file for hg19 was retrieved on August 23, 2017, and the hg38 version was retrieved on September 22, 2019. Both files can be downloaded from our data repository [97] at figshare. To further restrict the human coding regions, we only took the intersection between UCSC Coding regions and the Ensembl Exon regions.

### Low complexity regions

Low complexity regions were identified using an implementation of sdust [32] from https://github.com/lh3/minimap2, utilizing default parameters (-w 64 -t 20). The entire hg19 or hg38 genome file was processed to produce a bed file. Low complexity regions which overlapped with Ensembl Exon regions (GRCh37-75 for hg19 and GRCh38-97 for hg38) were extracted using bedtools [98] intersect. 5-nt was padded to both ends of each interval.

### Consensus target region

The consensus target region (CTR) [97] is primarily defined by the intersection of (i) the Interval4 regions, (ii) the human coding regions, and (iii) the NIST high-confidence regions. Interval4 is simply the intersection of the targeted design regions for WES1–4. CTR was generated for both hg19 and hg38. The targeted regions for WES1, which were designed in hg38, were lifted over to hg19 using the UCSC LiftOver tool. The targeted regions for WES2–4, which were designed in hg19, were lifted over to hg38. Finally, the low complexity regions were excluded. The size of CTR is 22,694,348 in hg19 and 21,710,990 in hg38 and shown in comparison to other regions of interest in Additional fie1; Table S2.

### Rules for determining Class 1 positive variants (by each genome version)

There were a diversity of variant calling pipelines used by members of the SEQC consortium. We asked the bioinformaticians of each organization to develop their preferred pipelines with their best expertise to call variants on their selected WES and WGS datasets. For WES datasets, there were twenty-two pipelines developed by nine teams as shown in Additional file 1: Table S3. All teams selected certain WES datasets for which they had their best experience. Each WES1–3 dataset was analyzed by seven to fourteen different pipelines on either reference genome versions. All teams selected certain WES datasets for which they had their best experience. Each WES1–3 dataset was analyzed by seven to fourteen different pipelines on either reference genome versions.

The freedom of choice of datasets, reference genome versions, mappers, and callers as well as parameters and filters created diversity and resulted in marginal to significantly different results between variant calling pipelines on the same input data. We investigated the similarity of the pipeline-library combinations (PLCs) in terms of variant calling on the individual UHR cell lines. The results did not fall into simple patterns. Many PLCs provided quite similar variant calls while outlier pipelines were also detected.

To achieve consensus, we defined a Class 1 positive variant as having at least half of the PLCs call the variant with alternative allele frequency (VAF) no less than 10% on the same cell line for each of WES1–3. The variant list for each cell line was then pooled together across the cell lines by kit to generate a non-redundant list of variants for the pooled Sample A by kit. We then took the intersection of the non-redundant variants called for each of WES1, WES2, and WES3 to compose the Class 1 list of variants, which are defined as known positives in this study. We also considered the region for which we would define the Class 1 variants. Only variants called within the CTR were termed as Class 1 known positives. We performed this procedure for hg19 then conducted a liftover for mapping to hg38 genome positions.

The Class 1 positives are not a complete list of variants for pooled Sample A. However, given (i) the large sequencing depth, (ii) only variants with VAF ≥ 10% were considered by cell line, (iii) the variants were selected by voting with multiple PLCs with diversity of callers and also agreed among three WES datasets, and (iv) a random sample of 114 (including 33 at VAF < 5%) of these variants were 100% orthogonally verified by ddPCR, we consider the Class 1 variants to be known positives.

### Rules for determining Class 2 positive variants

Variants from WGS 10X Chromium libraries of individual cell lines were examined in high-confidence coding regions of COSMIC genes but outside of CTR regions that includes common design coding regions to all WES kits utilized in this study. If the same variant was called by two different WGS variant callers (Freebayes and GATK) and was called by SomaticSeq from two different WES kits in the same cell line, then the variant was categorized as a Class 2 positive variant. The magnitude of the Class 2 positives was much smaller (by two orders of magnitude) than those of the Class 1 positives (359 vs. 42,211). This is primarily due to Class 2 positives being restricted to COSMIC genes and from the WES kits having highly overlapping target regions, which exclude these loci from being considered as a Class 2 variant. However, the number of Class 2 positives in COSMIC genes with VAF < 20% (210) is more than 10% of the identified total positives with VAF < 20% in COSMIC genes (1653).

### Complex variants including multi-allelic variants and multi-nucleotide variants (MNVs)

Particular attention should be paid to complex variants as there is typically variability in the manner in which they are documented. For example, we detected 32 loci that had distinct alternative alleles between various cell lines. When pooled into Sample A, these will appear as multi-allelic variants. For simplicity for analytical performance calculations and given the small magnitude of these variants, they were removed from our official positive variant list. However, we are quite confident in our assessment that these 32 loci are multi-allelic in Sample A and those 32 loci (64 variants) are provided in Additional file 1: Table S11. In addition to multi-allelic variants, we have also documented certain SNVs/indels in phase with other SNVs/indels which could be properly termed MNVs. Our list of positives includes over 200 dinucleotide and some trinucleotide positions that can more properly be termed as MNVs as well as other potential MNVs.

When evaluating the performance of cancer panels, it is important that proper guidelines and tools be used relative to the known content of complex reference samples [99]. We recommend using tools such as RTG vcfeval when reviewing panel results with Sample A positives to resolve the ambiguities with multiple representation of variants so that false positive and false negatives are best characterized.

### Rules for determining known negative variant positions

In addition to known positives, we also determined a set of negative positions. The total read depth (DP), reference allele depth (RDP) and alternative allele depth (ADP) were counted with the samtools [24] (v1.9) mpileup function at each position. Positions with DP < 50 in one library were removed. DP and RDP of the two libraries for each cell line were summed together and the reference allele frequency (RF), alternative allele frequency (AF) recalculated. We then applied filters by merged DP ≥ 125 and recalculated RF ≥ 99%. A simple averaged RF of the ten cell lines was calculated and filtered by average RF ≥ 99.5%. We only considered negative positions inside the CTR (which is a subset of the intersection of the coding target regions for the WES1–3) as used for the Class 1 positives. We did this procedure for both genome versions hg19 and hg38. We then lifted over the hg19 version to hg38 (noted “hg19ToHg38”) and hg38 version to hg19 (noted “hg38ToHg19”) with R package rtracklayer [100] (v1.42.0). For INDEL calls with the simple samtools mpileup, the adjacent positions from both ends of the INDEL calls were removed. To further remove possible variants, we employed a similar route to call negative positions through WES1–3 data of pooled Sample A. The DP and RDP of all libraries of each of WES1–3 were summed together. Any positions with total DP ≥ 500, ADP > 2, and AF ≥ 0.002 were removed. The adjacent positions from both ends of the INDEL calls were also removed. Finally, we took the intersection between hg19 and hg38ToHg19, or between hg38 and hg19ToHg38 as known negatives for the pooled Sample A in this study. This stringent process led to 10,229,649 negative positions in hg19 and 10,208,086 negative positions in hg38.

### ddPCR methods (orthogonal validation)

Droplet digital PCR (ddPCR) technology uses water-oil emulsion to partition the DNA sample into thousands of nanoliter-sized droplets and thus generates thousands of measurement data points for independent PCR amplification event [101]. We chose Bio-Rad’s PrimerPCR™ Assays for ddPCR as a proven technology for orthogonal validation to NGS with a convenient pipeline for custom assay design. Given a 100 ng DNA sample, PrimerPCR assays accurately detect mutant DNA in 10,000-fold wild type background (Bio-Rad Droplet Digital PCR Application Guide).

In total, 375 targets were selected for ddPCR validation and testing. These targets represented multiple variants variant types such as Class 1 and Class 2 positive variants, known negatives, and some investigational loci. SNVs, indels, and variants under influence of CN variation were examples of variant types. We established three criteria to guide the process of target selection: (i) preference was given to targets with clinical relevance or those in cancer related genes such as COSMIC genes; (ii) stratification with random selection: a sufficient number of targets were assigned to each category and then targets were randomly selected within each category; (iii) targets with close-by variant(s) were avoided in order to minimize interference with the primer or probe of the ddPCR assay. Mapped COSMIC IDs or sequence around the target were used as the input for online assay design. Only targets with a successful assay design were kept for further consideration. The assays and their categories are listed in Additional file 1: Table S12. Below is the description and count of assays in each category.

Category 0: Variants with documented oncology relevance. Subset 0.1 includes 19 hotspot variants detected in cell lines or Sample A (investigational). Subset 0.2 includes 39 targets which are known negatives. Subset 0.3 includes 9 investigational targets as putative negatives.

Category 1: Class 1 variants (SNV or small indels) in 219 COSMIC Classic genes. Out of 340 designable targets, 132 variants were randomly selected. Seventeen assays were excluded due to design errors discovered after the fact. As a result, there were 115 assays randomly selected as Class 1 variants from COSMIC genes.

Category 2: Class 2 variants (SNV or indels) in COSMIC genes. From 359 Class 2 variant targets, 50 variants were selected for ddPCR assay design, all in COSMIC genes as all Class 2 variants are in COSMIC genes.

Category 3: Low VAF Class 1 variants. These variants have VAF in Sample A ranging from 1 to 2.5%. This category includes 40 positives that are present in only one cell line that is not BLY and then 10 variants that are present only in BLY.

Category 4: Challenging and other indels. All candidate indels were first called in WES data and confirmed by 10X Genomics WGS data. Indels longer than 20 bps could not be designed. Out of all designable indels, 50 indels were randomly chosen with one design failure leaving 49 ddPCR assays for indels. Challenging indels included 14 indels that were low frequency (i.e., VAF < 10%), 8 complex (insertion or deletion at least 2 bases in length), and 20 that were both. In addition, there were 7 additional simple Indels.

Category 5: Variants under greater CN influence. A list of about 40 potential candidates was first generated for 13 regions under CN influence in various individual cell lines. At least one designable variant was then manually chosen to represent each region. For certain wide regions, two variants were chosen. In total, there were 20 Class 1 positives assayed by ddPCR under greater CN influence.

Category 6: Additional investigational low VAF variants (not Class 1 or Class 2) called by Accugenomics pipeline [102] that incorporate the background sequencing error rate inferred from Accugenomics controls. Potential variants were reported in two pan-cancer panels from the Pan-Cancercross-lab study [102] using Sample A. There were 24 investigational ddPCR assays in this category.

Each ddPCR assay was used to test gDNA from 10 individual UHR cell lines and reference Samples A, B, C, D, and E. The sample input amount for 10 UHR cell lines and Sample A was 11 ng. The input amount was chosen to enable variant allele detection sensitivity of 0.1% while moderately conserving samples. Eight assays and 11 samples were multiplexed to a 96-well plate with one well for a no-template control (NTC) sample for each assay. The input amount was increased to 55 ng for more sensitive detection in reference Samples B, C, D, and E. To ensure the results were reproducible, Samples B, D, and E were tested in duplicate wells. Twelve assays and four samples (occupying seven wells) were multiplexed to a 96-well plate with one well for an NTC sample for each assay. In total, 84 plates were used to implement the whole ddPCR experiment. Following Bio-Rad’s ddPCR protocol, samples were first fragmented by the enzyme specified in each assay design and loaded to the QX200™ AutoDG instrument (Bio-Rad) for analysis. Data analysis was then carried out through Bio-Rad’s software with the users manually checking and, if necessary, adjusting cluster thresholds for each assay. After clustering, the magnitude of group cluster membership of individual droplets (negative for both alleles, allele1 occupancy, allele2 occupancy, occupancy by both alleles) was evaluated for consistency with expected values from binomial distribution assumptions related to joint probabilities for distinct alleles occupying the same droplet, single alleles occupying a droplet and for neither allele occupying a droplet. Inconsistencies were corrected and assays not achieving expected distributional frequencies were deemed as failed. A description of the loci tested by ddPCR is also provided in Additional file 1: Table S12.

### Analysis of cell line mixture proportions

In principle, it should be possible to calculate/estimate the theoretical allele frequencies in Sample A for each of the variants identified in the individual cell lines starting from the allele frequencies and/or the read counts at a locus determined independently for each cell line. This would provide evidence that the mixtures were performed properly and are at expected levels in the pooled final reference. We attempted to build models that used read counts in each cell line related to known positives to predict their VAF by locus in Sample A. VAFs calculated on the deeply sequenced sample A were used to estimate the error of the prediction. To simplify analysis and given the abundance of variants available, we identified private known positives by cell line and performed a linear regression tuned on the private known positives so that the VAF from positives of Sample A could be expressed as a linear combination of the cell line VAFs (positive or 0). The model multipliers were obtained by solving the linear system whose matrix is computed as the convex combination of the depth and the alternate allele counts on a chosen subset of all possible genomic positions. While some models appeared more reasonable than others, we observed large variations in the β estimates for the cell line mix ratios for different models and subsets, resulting in fundamentally unreliable VAF predictions. The instability in estimates was possibly due to the large number of rearrangements in the cancer cell lines, creating inconsistent depth at a given locus from cell line to cell line. A simple linear regression of the VAF of each cell line onto the Sample A VAF provided reasonable if oversimplified results that indicated the cell lines were properly mixed (given the approximate 1:2 mixture of TLY into BLY described elsewhere).

### Funding

This research was supported, in part, by the Intramural Research Program of the National Institutes of Health (NIH), National Institute of Environmental Health Sciences (NIEHS). This research has also been financially supported by the MEYS of the CR under the project CEITEC 2020 (LQ1601), by MH CR, grant No. (NV19–03-00091). Part of this work was carried out with the support of research infrastructure EATRIS-CZ, ID number LM2015064, funded by MEYS CR. Leming Shi and Yuanting Zheng were supported by the National Key R&D Project of China (2018YFE0201600), the National Natural Science Foundation of China (31720103909), and Shanghai Municipal Science and Technology Major Project (2017SHZDZX01).

## Supplementary Information

**Additional file 1:.** Table S1. Basic library statistics from the cell line library runs WES1–4 and WGS1. Table S2. Design size of each kit, exome, and other considerations (bed file sizes, hg19 and hg38). Table S3. Listing of enrichment methods and pipeline runs along with respective genomic versions used for creating the variant set. RSS02 was used for confirmation and not for identification of variants (○). Table S4. The number of variants identified as ground truth in Sample A broken down into different categories. Table S5. Basic sequencing statistics for independent testing of pooled Sample A replicates from three kits: WES1 Roche MedExome, WES2- IDT xGen, WES3 – Agilent SureSelect (unmerged BAM statistics). Table S6. a: Descriptive statistics for the detection of the Class 1 and 2 positives by SomaticSeq in the union of pooled Sample A libraries (merged-BAM libraries) or the in silico Sample A compared with the detection of Class 1 and 2 positives by SomaticSeq when examining individual cell lines. b: Table showing exemplar results of raw sensitivity for Class 1 cell line variants for selected pipelines and WES kits by cell line. If the variant was detected in either cell line replicate, it was counted as detected by that pipeline for this table. Table S7. Summary of variant class types for orthogonal ddPCR validation of variants in Sample A. Complex indels involve insertions or deletions of more than two bases. Challenging indels included low frequency indels, complex indels, or both. Low VAF for Indels was defined as VAF ≤10% in Sample A for this table. Low VAF for SNVs was defined as VAF ≤ 5% in Sample A for this table. Table S8. List of the number of variants identified per cell line. The difference in magnitudes between the two genomic versions is due to variations in the NIST high-confidence (benchmark) regions between the two genome reference versions. Table S9. Pathogenic or important variants identified as being present in Sample A. Table S10. List of number of variants in Sample A identified by type and by chromosome. A breakdown is provided by allele frequency ranges. Table S11. List of 32 verified multi-allelic SNVs that were not otherwise identified and counted as positives. The observed VAF in pool A for each multi-allele variant is relative to reference. Thus, the individual variants VAFs may add up to greater than 100%. Table S12. Basic information of the ddPCR assays and their variant class. LF is for Low frequency.

**Additional file 2:.** Fig. S1. Unique Fragments, total Deduplicated Depth, and Coverage of Sample A by Exome enrichment kit replicate. WES1–3 are Illumina sequencing libraries. Fig. S2. Data related to Thermo Fisher’s WES panel related to its a) sensitivity by cell line at VAF greater than 0.1 and 0.2, b) total number of WES4 variants called relative to total positives by cell line at VAF greater than 0.1 and 0.2 and c) precision (or positive predictive value) of the WES4 panel on individual cell line variants. Fig. S3. TLY VAF profile at top where common variants from dbSNP are in red (het) or green (hom). Variants not found in dbSNP are in orange. Since TLY has an asymmetric VAF profile indicated in by the somatic variants in orange, the reasonable explanation (combined with cytology) is that TLY is inherently tetraploid with some noticeable large sCNA (e.g., chr 4, 7, 8, 17 and 20 are most noteworthy) and that the somatic variants are all near 0.25 as somatic changes are randomly scattered among the two chromosome pairs from the cancer cell, rarely affecting both. In essence, cell line TLY is a fusion of a normal and cancerous cell. Fig. S4. VAF histogram of Sample A for Class 2 variants only (COSMIC genes but not in CTR). Fig. S5. a-j) Histograms of VAF using all positives (Class 1 and Class 2) contributed by each of the 10 cell lines. Class 1 positives are > 99% of all positives. k-t) Histograms of VAF using only Class 2 positives contributed by each of the 10 cell lines. Fig. S6. Concordance in VAF of Pooled Sample A between bait captures, in silico results, merged-BAM library results and the averaging of individual cell line results. Results between same kits are slightly more highly correlated. Cell line average results are concordant except for a small number of variants, many related to CNA influences from individual cell lines. Fig. S7. Concordance of putative positives (variants) from CTR using WES1–3. The area of each figure represents the magnitude of the overlap between the different kits and the different methods: directly interrogating merged-BAM triplicates of Sample A or in silico Sample A. We observe 96% of putative positives from the CTR region are detected in all three kits in both the merged-BAM Sample A and the in silico Sample A using only the single SomaticSeq pipeline. Only 0.24% are not detected by SomaticSeq in either in silico Sample A or merged-BAM Sample A. Fig. S8. CN and other data establishing BLY as a mixture of original BLY and TLY. VAF profile at top consists of only common SNPs as well as the log ratio of TLY and BLY depths (compared to normal reference Sample B). The green regions indicate TLY somatic CN loss and the red regions indicate TLY somatic CN gain. Gray regions are somatic CN gain or loss examples that only exist in BLY and thus must have come from the original B lymphocyte cell line. All TLY gains and losses clearly appear exactly in BLY except for the gain in chr4. Thus, the original B lymphocyte cell line must have a corresponding chr4 loss, which was verified by subsequent CNV analysis of the original B lymphocyte cell line (data not shown). Fig. S9. Each point is a putative variant either associated with canonical het and homozygous non-reference alleles (blue), putative variants with replicated non-zero VAF but outside canonical regions (red), or putative variants observed in only one replicate (gray). The replicate VAF of the Normal B Reference cell line is shown for the Q201 pipeline for all variants in coding regions (top) and then only those in high-confidence (HC) coding regions (bottom) by capture kit. It is expected that the Normal B Reference will only have canonical het and homozygous non-reference alleles, which is more compatible with the graphs illustrating variants from HC regions only. The red and gray positions, indicating a deviation from canonical states, range from 17% to more than 21% of putative variants over the three WES kits when outside HC regions. However, for HC-only regions, 97.8% to > 99% of the putative variants are in blue representing expected VAF for canonical alleles. Observed variation of VAF near 0.5 and 1.0 is due to stochastic sampling and sequencing error. Fig. S10. As a supplement to Fig. 3a, this figure illustrates the difference observed in VAF between ddPCR and consensus WES results across a range of VAF and different types of variants tested by ddPCR. One can see the bias in the VAF estimate from WES and how it is more pronounced at higher VAF values and for indels vs. SNVs. Fig. S11. Results from a series of simulations using 2 to 12 independent unrelated cell lines from 13 cell line candidates and simulating VAF and number of variants using a mixture of a random subset of them. The yellow line indicates the median of the typical VAF given the various mixtures of cell lines. The orange dashed lines indicate the median of the 25th (Q1) and 75th (Q3) percentiles of the VAF given the various mixtures of the cell lines. The solid blue lines indicate the median total coding variants that one could identify in the CTR while the dashed blue line indicates the typical (median) total number of coding variants that one expects to have an allele frequency less than 10%. Corresponding values observed for Sample A are also shown.

**Additional file 3.** Supplementary information. Bias in reported WES allele frequencies. Recommended process for reference sample creation.

**Additional file 4.** Review history.

## Data Availability

Important data items from the consortium reference sample effort that we are disclosing include: a) A list of identified variants or positives (> 40,000) and negative positions (> 10,000,000) in the coding regions of the genome for Pool Sample A [97]. b) Pool Sample A, in silico mixed Sample A and Reference Sample B FASTQ files from the various whole exome sequencing enrichment protocols and platforms [103]. c) Droplet digital PCR data from the independent orthogonal validation effort including the design file [97].
