# Supplementary material for: A verified genomic reference sample for assessing performance of cancer panels detecting small variants of low allele frequency
Source: Genome Biol. 2021 Apr 16;22:111. doi: 10.1186/s13059-021-02316-z (PMC8051128; doi:10.1186/s13059-021-02316-z)
Supplement: Supplementary file 1 — Additional file 1:. Table S1. Basic library statistics from the cell line library runs WES1–4 and WGS1. Table S2. Design size of each kit, exome, and other considerations (bed file sizes, hg19 and hg38). Table S3. Listing of enrichment methods and pipeline runs along with respective genomic versions used for creating the variant set. RSS02 was used for confirmation and not for identification of variants (○). Table S4. The number of variants identified as ground truth in Sample A broken down into different categories. Table S5. Basic sequencing statistics for independent testing of pooled Sample A replicates from three kits: WES1 Roche MedExome, WES2- IDT xGen, WES3 – Agilent SureSelect (unmerged BAM statistics). Table S6. a: Descriptive statistics for the detection of the Class 1 and 2 positives by SomaticSeq in the union of pooled Sample A libraries (merged-BAM libraries) or the in silico Sample A compared with the detection of Class 1 and 2 positives by SomaticSeq when examining individual cell lines. b: Table showing exemplar results of raw sensitivity for Class 1 cell line variants for selected pipelines and WES kits by cell line. If the variant was detected in either cell line replicate, it was counted as detected by that pipeline for this table. Table S7. Summary of variant class types for orthogonal ddPCR validation of variants in Sample A. Complex indels involve insertions or deletions of more than two bases. Challenging indels included low frequency indels, complex indels, or both. Low VAF for Indels was defined as VAF ≤10% in Sample A for this table. Low VAF for SNVs was defined as VAF ≤ 5% in Sample A for this table. Table S8. List of the number of variants identified per cell line. The difference in magnitudes between the two genomic versions is due to variations in the NIST high-confidence (benchmark) regions between the two genome reference versions. Table S9. Pathogenic or important variants identified as being present in Sample A. Table S10. List of [file 13059_2021_2316_MOESM1_ESM.docx]

**Table S1: Basic library statistics from the cell line library runs WES1-4 and WGS1**

| **1a WES Sequencing: WES kits 1-3 (Illumina)** | | |  |  |  |  |  |
| --- | --- | --- | --- | --- | --- | --- | --- |
| **Cell Line** | **Replicate** | **paired reads (2) x 100** | **unmapped reads** | **Est. % Dup** | **Mapped %** | **Effective Bases** | **Library Enrichment Kit** |
| B | WES3-Lib1 | 1.903E+08 | 261626 | 47% | 99.9% | 2.022E+10 | Agilent |
| B | WES3-Lib2 | 1.638E+08 | 257465 | 30% | 99.8% | 2.294E+10 | Agilent |
| B-Lymphocyte | WES3-Lib1 | 1.657E+08 | 156508 | 43% | 99.9% | 1.900E+10 | Agilent |
| B-Lymphocyte | WES3-Lib2 | 1.599E+08 | 149826 | 33% | 99.9% | 2.140E+10 | Agilent |
| Brain | WES3-Lib1 | 1.626E+08 | 183741 | 31% | 99.9% | 2.256E+10 | Agilent |
| Brain | WES3-Lib2 | 1.620E+08 | 185173 | 43% | 99.9% | 1.853E+10 | Agilent |
| Breast | WES3-Lib1 | 1.730E+08 | 250690 | 30% | 99.9% | 2.433E+10 | Agilent |
| Breast | WES3-Lib2 | 1.652E+08 | 176434 | 59% | 99.9% | 1.359E+10 | Agilent |
| Cervix | WES3-Lib1 | 1.575E+08 | 193483 | 29% | 99.9% | 2.237E+10 | Agilent |
| Cervix | WES3-Lib2 | 1.703E+08 | 212425 | 38% | 99.9% | 2.099E+10 | Agilent |
| Liposarcoma | WES3-Lib1 | 1.559E+08 | 161700 | 43% | 99.9% | 1.778E+10 | Agilent |
| Liposarcoma | WES3-Lib2 | 1.650E+08 | 179396 | 36% | 99.9% | 2.095E+10 | Agilent |
| Liver | WES3-Lib1 | 1.438E+08 | 125886 | 32% | 99.9% | 1.965E+10 | Agilent |
| Liver | WES3-Lib2 | 1.513E+08 | 171725 | 33% | 99.9% | 2.027E+10 | Agilent |
| Macrophage | WES3-Lib1 | 1.817E+08 | 198929 | 34% | 99.9% | 2.412E+10 | Agilent |
| Macrophage | WES3-Lib2 | 1.401E+08 | 192965 | 34% | 99.9% | 1.857E+10 | Agilent |
| Skin | WES3-Lib1 | 1.742E+08 | 191715 | 36% | 99.9% | 2.223E+10 | Agilent |
| Skin | WES3-Lib2 | 1.520E+08 | 192242 | 42% | 99.9% | 1.775E+10 | Agilent |
| Testis | WES3-Lib1 | 1.737E+08 | 207035 | 37% | 99.9% | 2.170E+10 | Agilent |
| Testis | WES3-Lib2 | 1.586E+08 | 178342 | 46% | 99.9% | 1.708E+10 | Agilent |
| T-Lymphoblast | WES3-Lib1 | 1.606E+08 | 251185 | 39% | 99.8% | 1.960E+10 | Agilent |
| T-Lymphoblast | WES3-Lib2 | 1.474E+08 | 153955 | 26% | 99.9% | 2.176E+10 | Agilent |
|  |  |  |  |  |  |  |  |
|  |  | **paired reads (2) x 150** | |  |  |  |  |
| B | WES2-Lib1 | 9.033E+07 | 108164 | 41% | 99.9% | 1.062E+10 | IDT |
| B | WES2-Lib2 | 8.115E+07 | 137842 | 38% | 99.8% | 9.973E+09 | IDT |
| B-Lymphocyte | WES2-Lib1 | 6.327E+07 | 79844 | 33% | 99.9% | 8.504E+09 | IDT |
| B-Lymphocyte | WES2-Lib2 | 6.801E+07 | 83826 | 34% | 99.9% | 8.934E+09 | IDT |
| Brain | WES2-Lib1 | 7.569E+07 | 110910 | 45% | 99.9% | 8.246E+09 | IDT |
| Brain | WES2-Lib2 | 7.612E+07 | 150414 | 49% | 99.8% | 7.727E+09 | IDT |
| Breast | WES2-Lib1 | 7.041E+07 | 149374 | 43% | 99.8% | 8.002E+09 | IDT |
| Breast | WES2-Lib2 | 7.332E+07 | 94433 | 45% | 99.9% | 8.055E+09 | IDT |
| Cervix | WES2-Lib1 | 6.904E+07 | 81816 | 35% | 99.9% | 9.002E+09 | IDT |
| Cervix | WES2-Lib2 | 7.260E+07 | 69638 | 37% | 99.9% | 9.166E+09 | IDT |
| Liposarcoma | WES2-Lib1 | 7.788E+07 | 94266 | 36% | 99.9% | 9.927E+09 | IDT |
| Liposarcoma | WES2-Lib2 | 6.970E+07 | 104885 | 33% | 99.8% | 9.376E+09 | IDT |
| Liver | WES2-Lib1 | 6.624E+07 | 83481 | 40% | 99.9% | 7.983E+09 | IDT |
| Liver | WES2-Lib2 | 6.821E+07 | 87433 | 44% | 99.9% | 7.694E+09 | IDT |
| Macrophage | WES2-Lib1 | 6.627E+07 | 75139 | 40% | 99.9% | 7.970E+09 | IDT |
| Macrophage | WES2-Lib2 | 6.845E+07 | 85642 | 34% | 99.9% | 9.024E+09 | IDT |
| Skin | WES2-Lib1 | 7.905E+07 | 111966 | 52% | 99.9% | 7.540E+09 | IDT |
| Skin | WES2-Lib2 | 8.596E+07 | 130657 | 50% | 99.8% | 8.520E+09 | IDT |
| Testis | WES2-Lib1 | 8.478E+07 | 113259 | 44% | 99.9% | 9.471E+09 | IDT |
| Testis | WES2-Lib2 | 7.786E+07 | 110218 | 43% | 99.9% | 8.907E+09 | IDT |
| T-Lymphoblast | WES2-Lib1 | 7.416E+07 | 88281 | 33% | 99.9% | 9.976E+09 | IDT |
| T-Lymphoblast | WES2-Lib2 | 7.243E+07 | 87867 | 33% | 99.9% | 9.667E+09 | IDT |
|  |  |  |  |  |  |  |  |
|  |  | **paired reads (2) x 150** | |  |  |  |  |
| B | WES1-Lib1 | 1.017E+08 | 130873 | 18% | 99.9% | 1.660E+10 | Roche |
| B | WES1-Lib2 | 1.146E+08 | 150317 | 19% | 99.9% | 1.850E+10 | Roche |
| B-Lymphocyte | WES1-Lib1 | 8.390E+07 | 55315 | 21% | 99.9% | 1.333E+10 | Roche |
| B-Lymphocyte | WES1-Lib2 | 7.936E+07 | 59991 | 18% | 99.9% | 1.296E+10 | Roche |
| Brain | WES1-Lib1 | 9.016E+07 | 67345 | 20% | 99.9% | 1.442E+10 | Roche |
| Brain | WES1-Lib2 | 1.035E+08 | 92508 | 22% | 99.9% | 1.605E+10 | Roche |
| Breast | WES1-Lib1 | 8.779E+07 | 64887 | 20% | 99.9% | 1.400E+10 | Roche |
| Breast | WES1-Lib2 | 8.742E+07 | 69940 | 21% | 99.9% | 1.388E+10 | Roche |
| Cervix | WES1-Lib1 | 8.567E+07 | 63447 | 21% | 99.9% | 1.344E+10 | Roche |
| Cervix | WES1-Lib2 | 9.326E+07 | 71699 | 22% | 99.9% | 1.461E+10 | Roche |
| Liposarcoma | WES1-Lib1 | 8.737E+07 | 63523 | 19% | 99.9% | 1.410E+10 | Roche |
| Liposarcoma | WES1-Lib2 | 9.767E+07 | 71522 | 21% | 99.9% | 1.541E+10 | Roche |
| Liver | WES1-Lib1 | 7.477E+07 | 48747 | 19% | 99.9% | 1.209E+10 | Roche |
| Liver | WES1-Lib2 | 9.481E+07 | 68157 | 22% | 99.9% | 1.478E+10 | Roche |
| Macrophage | WES1-Lib1 | 1.010E+08 | 75081 | 22% | 99.9% | 1.580E+10 | Roche |
| Macrophage | WES1-Lib2 | 8.711E+07 | 60692 | 19% | 99.9% | 1.403E+10 | Roche |
| Skin (melanoma) | WES1-Lib1 | 8.356E+07 | 70444 | 20% | 99.9% | 1.334E+10 | Roche |
| Skin (melanoma) | WES1-Lib2 | 9.761E+07 | 79586 | 22% | 99.9% | 1.514E+10 | Roche |
| Testis | WES1-Lib1 | 6.227E+07 | 51793 | 20% | 99.9% | 9.895E+09 | Roche |
| Testis | WES1-Lib2 | 9.586E+07 | 79554 | 23% | 99.9% | 1.474E+10 | Roche |
| T-Lymphoblast | WES1-Lib1 | 8.479E+07 | 58963 | 20% | 99.9% | 1.352E+10 | Roche |
| T-Lymphoblast | WES1-Lib2 | 8.085E+07 | 60730 | 20% | 99.9% | 1.299E+10 | Roche |
|  |  |  |  |  |  |  |  |
|  |  |  |  |  |  |  |  |
|  |  |  |  |  |  |  |  |
|  |  |  |  |  |  |  |  |
|  |  |  |  |  |  |  |  |
|  |  |  |  |  |  |  |  |
| **1b WES Sequencing (Thermo Fisher)** | |  |  |  |  |  |  |
| **Cell Line** | **Replicate** | **Mapped  Single Reads** | **Mean Read Length** | **Est. % Dup** |  | **Effective Bases** | **Library Enrichment Kit** |
| B | WES4-Lib1 | 3.478E+07 | 175.5 | NA |  | 5.485E+09 | Thermo |
| B-Lymphocyte | WES4-Lib1 | 3.378E+07 | 179.3 | NA |  | 5.395E+09 | Thermo |
| Brain | WES4-Lib1 | 4.255E+07 | 176.9 | NA |  | 6.764E+09 | Thermo |
| Breast | WES4-Lib1 | 5.020E+07 | 180.7 | NA |  | 8.214E+09 | Thermo |
| Cervix | WES4-Lib1 | 4.135E+07 | 182.6 | NA |  | 6.835E+09 | Thermo |
| Liposarcoma | WES4-Lib1 | 4.942E+07 | 187.7 | NA |  | 8.455E+09 | Thermo |
| Liver | WES4-Lib1 | 4.699E+07 | 181.0 | NA |  | 7.659E+09 | Thermo |
| Macrophage | WES4-Lib1 | 4.256E+07 | 183.5 | NA |  | 6.981E+09 | Thermo |
| Skin (melanoma) | WES4-Lib1 | 5.212E+07 | 183.1 | NA |  | 8.596E+09 | Thermo |
| Testis | WES4-Lib1 | 4.003E+07 | 175.4 | NA |  | 6.291E+09 | Thermo |
| T-Lymphoblast | WES4-Lib1 | 4.263E+07 | 181.1 | NA |  | 6.871E+09 | Thermo |

**1c - WGS Sequencing**

| **Cell Line** | **Number of Reads** | **Median Insert Size** | **WGS Average Depth** | **Percentage Mapped Reads** | **PCR Dup. Rate** |
| --- | --- | --- | --- | --- | --- |
| B | 1.5734E+09 | 378 | 64.47 | 93.4% | 11.5% |
| B-Lymphocyte | 1.7402E+09 | 335 | 72.92 | 94.1% | 10.6% |
| Brain | 1.6414E+09 | 393 | 69.43 | 94.6% | 10.3% |
| Breast | 1.6336E+09 | 392 | 68.07 | 93.6% | 10.3% |
| Cervix | 1.6679E+09 | 353 | 65.23 | 92.8% | 10.4% |
| Liposarcoma | 1.7602E+09 | 394 | 74.86 | 94.5% | 10.1% |
| Liver | 1.7243E+09 | 394 | 72.48 | 93.8% | 10.0% |
| Macrophage | 1.7034E+09 | 389 | 71.33 | 93.7% | 11.5% |
| Skin (melanoma) | 1.7465E+09 | 393 | 74.06 | 94.4% | 9.9% |
| Testes | 1.6900E+09 | 399 | 72.41 | 95.2% | 11.5% |
| T-Lymphoblast | 1.6830E+09 | 379 | 69.40 | 92.9% | 10.9% |

**Table S2:** **Design size of each kit, exome, and other considerations (bed file sizes, hg19 and hg38)**

| Regions of Interest | Hg19 Size (b) | Hg38 Size (b) |
| --- | --- | --- |
| Roche MedExome Target Regions (WES1) | 65,630,009 | 66,064,715 |
| IDT xGen Design Regions (WES2) | 38,874,504 |  |
| Agilent SureSelect Design Regions (WES3) | 60,456,963 |  |
| Thermo Fisher AmpliSeq Exome Effective Regions (WES4) | 46,347,343 |  |
| Exome (Human Coding) Region | 33,945,911 | 34,310,542 |
| Interval4 Coding Region | 27,228,131 | 27,231,396 |
| High-Confidence Region (Exome) | 27,778,707 | 26,416 ,304 |
| Consensus Target Region (CTR) | 22,694,348 | 21,710,990 |

**Table S3: Listing of enrichment methods and pipeline runs along with respective genomic versions used for creating the variant set. RSS02 was used for confirmation and not for identification of variants (○).**

| **pipeline** | **mapper** | **version** | **caller** | **version** | **hg19** | | | | **hg38** | | | |
| --- | --- | --- | --- | --- | --- | --- | --- | --- | --- | --- | --- | --- |
|  |  |  |  |  | WES1 | WES2 | WES3 | WES4 | WES1 | WES2 | WES3 | WES4 |
| **AGL01** | bwa_mem | 0.7.10 | mpileup | 1.3.1 |  |  | ● |  |  |  |  |  |
| **AGL02** | bwa_mem | 0.7.10 | GATK unified genotype caller | v2.2-3-gde33222 |  |  | ● |  |  |  |  |  |
| **AGL03** | bwa_mem | 0.7.10 | Platypus | 0.8.1 |  |  | ● |  |  |  |  |  |
| **AGL04** | bwa_mem | 0.7.10 | Surecall | v3.5.1.46 |  |  | ● |  |  |  |  |  |
| **FBK01** | HISAT2 | v2.0.4 | Platypus | 0.8.1 |  | ● |  |  |  |  |  |  |
| **FUDAN01** | bwa_mem | v0.7.12-r1039 | Sentieon Haplotyper | 201611.02 |  | ● | ● |  | ● |  |  |  |
| **INGEMM** | bowtie2 |  | GATK and Annovar | v1.1.4 | ● |  |  |  |  |  |  |  |
| **NCTR01** | bwa_mem | 0.7.12 | freebayes | v1.1.0-46-g8d2b3a0 | ● | ● | ● |  | ● | ● | ● |  |
| **NCTR02** | bowtie2 | 2.3.2-legacy | freebayes | v1.1.0-46-g8d2b3a0 | ● | ● | ● |  | ● | ● | ● |  |
| **NCTR05** | bwa_mem | 0.7.12 | VarScan | v2.4.0 | ● | ● | ● |  | ● | ● | ● |  |
| **NCTR06** | bowtie2 | 2.3.2-legacy | VarScan | v2.4.0 | ● | ● | ● |  | ● | ● | ● |  |
| **NIEHS01** | bwa_mem | v0.7.15-r1140 | Mutect1 | v1.1.4 | ● | ● | ● |  |  |  |  |  |
| **Q201** | bwa_mem | 0.7.10 | vardict | 1.5.1 | ● | ● | ● |  | ● |  |  |  |
| **Q202** | bwa_mem | 0.7.10 | Sentieon tnscope | 201704 | ● | ● | ● |  | ● |  |  |  |
| **Q203** | bwa_mem | 0.7.10 | GATK HaplotypeCaller | v3.6-0-g89b7209 |  |  | ● |  |  |  |  |  |
| **RSSMSN01** | bwa_mem | 0.7.12 | mutect2 | 2.1-beta/GATK 4.0a | ● |  |  |  | ● |  |  |  |
| **RSSMSN02** | bwa_mem | 0.7.12 | vardict | 1.5.1 | ● |  |  |  | ● |  |  |  |
| **RSSMSN03** | bwa_mem | 0.7.12 | mpileup | 1.2 | ● |  |  |  | ● |  |  |  |
| **RSSMSN04** | bwa_mem | 0.7.12 | Sentieon tnscope | 201704.03 | ● |  |  |  | ● |  |  |  |
| **RSSMSN05** | bwa_mem | 0.7.17 | mutect2 | 4.0.6.0 |  |  |  |  | ● | ● | ● |  |
| **RSSMSN06** | bwa_mem | 0.7.17 | GATK HaplotypeCaller | 4.0.6.0 |  |  |  |  | ● | ● | ● |  |
| **RSS02** | bwa_mem |  | SomaticSeq | v2.7.0 | ○ | ○ | ○ |  | ○ | ○ | ○ |  |
| **TF01** | TMAP |  | tvc | 5.4-10 (4ecb828) |  |  |  | ● |  |  |  |  |

**Table S4: The number of variants identified as ground truth in Sample A broken down into different categories**

**
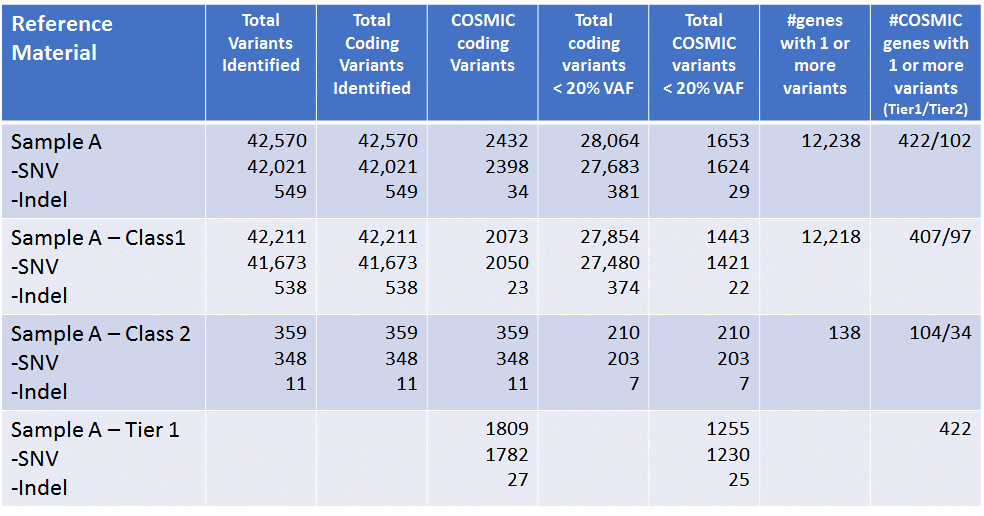
**

**Table S5: Basic sequencing statistics for independent testing of pooled Sample A replicates from three kits: WES1 Roche MedExome, WES2- IDT xGen, WES3 – Agilent SureSelect (unmerged BAM statistics)**

**Table S6a: Descriptive statistics for the detection of the Class 1 and 2 positives by SomaticSeq in the union of pooled Sample A libraries (merged-BAM libraries) or the *in silico* Sample A compared with the detection of Class 1 and 2 positives by SomaticSeq when examining individual cell lines**

| **SomaticSeq-only results** | **Identified Positives** | **Percent** |
| --- | --- | --- |
| Detected within *in silico* Sample A | 42415 | 99.64% |
| Detected within merged-BAM Sample A | 42447 | 99.71% |
| Detected within either *in silico* or merged-BAM Sample A | 42466 | 99.76% |
| Detected via individual cell lines | 42551 | 99.96% |
| Positives not called by SomaticSeq in any cell line or *in silico*, or merge-BAM Sample A | 19 | 0.04% |

**Table S6b: Table showing exemplar results of raw sensitivity for Class 1 cell line variants for selected pipelines and WES kits by cell line. If the variant was detected in either cell line replicate, it was counted as detected by that pipeline for this table.**

**Table S7: Summary of variant class types for orthogonal ddPCR validation of variants in Sample A. Complex indels involve insertions or deletions of more than two bases. Challenging indels included low frequency indels, complex indels, or both. Low VAF for Indels was defined as VAF ≤10% in Sample A for this table. Low VAF for SNVs was defined as VAF ≤ 5% in Sample A for this table.**

| **Class** | **Frequency** | | | **% of Grand Total** | |  |
| --- | --- | --- | --- | --- | --- | --- |
| Class 1 Indel (total) | 50 |  |  | | 13.3 | |
| Class 1 Indel (simple) |  | 7 |  | | 1.8 | |
| Class 1 Indel (low VAF COSMIC gene) |  | 1 |  | | 0.3 | |
| Class 1 Challenging Indels |  | 42 |  | | 11.2 | |
| Class 1 Indel (complex) |  |  | 8 | | 2.1 | |
| Class 1 Indel (low VAF) |  |  | 14 | | 3.7 | |
| Class 1 Indel (low VAF and complex) |  |  | 20 | | 5.3 | |
| Class 1 SNV (total) | 184 |  |  | | 49.1 | |
| Class 1 SNV (random - COSMIC gene) |  | 114 |  | | 30.4 | |
| Class 1 SNV (random/COSMIC low VAF) |  |  | 33 | | 8.8 | |
| Class 1 SNV (random/COSMIC not low VAF) |  |  | 81 | | 21.6 | |
| Class 1 SNV low VAF unique to a cell line |  | 40 |  | | 10.7 | |
| Class 1 SNV low VAF unique to BLY |  | 10 |  | | 2.7 | |
| Class 1 Var@CNA |  | 20 |  | | 5.3 | |
| Class 2 SNV (COSMIC gene) | 50 |  |  | | 13.3 | |
| Class 2 SNV (COSMIC & low VAF) |  | 12 |  | | 3.2 | |
| Class 2 SNV (COSMIC not low VAF) |  | 38 |  | | 10.1 | |
| Investigational | 52 |  |  | | 13.9 | |
| Hot-spot loci |  | 19 |  | |  | |
| Putative Negatives |  | 9 |  | |  | |
| Accugenomics-related |  | 24 |  | |  | |
| Negatives | 39 |  |  | | 10.4 | |
| Grand Total Positives | 284 |  |  | | 75.7 | |
| Grand Total Overall incl. Negatives/ Investigational | 375 |  |  | | 100.0 | |

**Table S8: List of the number of variants identified per cell line. The difference in magnitudes between the two genomic versions is due to variations in the NIST high-confidence (benchmark) regions between the two genome reference versions.**

| **# Variants** | **hg19** | **hg38** |
| --- | --- | --- |
| B (normal) | 13901 | 12623 |
| BLY | 23683 | 21472 |
| BRA | 12163 | 11020 |
| BRE | 12885 | 11735 |
| CRV | 15387 | 13968 |
| LIP | 12536 | 11430 |
| LIV | 13623 | 12381 |
| MAC | 12552 | 11359 |
| SKN | 13762 | 12483 |
| TES | 12415 | 11252 |
| TLY | 19177 | 17399 |

**Table S9: Pathogenic or important variants identified as being present in Sample A.**

Three reference sets were used:

- OncoKB. (OncoKB includes several levels of clinical evidence. Level 1 is for FDA-recognized biomarker predictive of response to an FDA-approved drug in this indication.)
- Cancer Hotspots. https://www.cancerhotspots.org/#/home
- Clinvar. <https://www.ncbi.nlm.nih.gov/clinvar/>. Filter by Clinical significance = pathogenic.

| **ID** | **Source** | **VAR_ID** | **Gene** | **Cell_line** | **Protein-change** | **Information** |
| --- | --- | --- | --- | --- | --- | --- |
| 1 | All | chr7-140453136-A-T | BRAF | LIP,SKN | V600E | Level 1; Q value 0; Pathogenic |
| 2 | Hotspots, Clinvar | chr17-7577022-G-A | TP53 | BLY,TLY | R306* | Q value 1.86E-26; Pathogenic |
| 3 | Hotspots, Clinvar | chr17-7577085-C-T | TP53 | BLY | E285K | Q value 1.42E-26; Pathogenic |
| 4 | Hotspots | chr17-7577529-A-T | TP53 | LIP | I251N | Q value 0.0066 |
| 5 | Hotspots, Clinvar | chr17-7578211-C-T | TP53 | BRA | R213Q | Q value 1.29E-203; Pathogenic |
| 6 | Hotspots | chr16-56873495-C-T | NUP93 | BLY,MAC,TLY | A733A | Q value 0.048 |
| 7 | Hotspots | chr5-67589147-A-G | PIK3R1 | LIP | K379E | Q value 0.0051 |
| 8 | Hotspots | chr8-68930087-T-G | PREX2 | LIV | L50V | Q value 4.17E-05 |
| 9 | Hotspots, Clinvar | chr1-115258748-C-A | NRAS | BLY,TLY | G12C | Q value 3.67E-130, Pathogenic |
| 10 | Hotspots | chr12-121416650-A-C | HNF1A | BRA,SKN,TES | I27L | Q value 0.032 |
| 11 | Hotspots | chr3-189587118-C-T | TP63 | SKN | R379C | Q value 8.73E-08 |
| 12 | Hotspots | chr2-204732714-A-G | CTLA4 | BLY,BRA,BRE,CRV,LIP,LIV,MAC,TES,TLY | T17A | Q value 0.024 |
| 13 | Hotspots | chr13-110435231-C-T | IRS2 | BLY,BRE,CRV,LIP,LIV,MAC,TLY | G1057D | Q value 8.54E-02 |
| 14 | Clinvar | chr10-115636390-G-T | NHLRC2 | BLY,TLY | D148Y | Pathogenic |
| 15 | Clinvar | chr10-70645376-A-C | STOX1 | CRV,MAC | E608D;E498D | Pathogenic |
| 16 | Clinvar | chr10-89717769-TA-T | PTEN | BLY,TLY | K26* | Pathogenic |
| 17 | Clinvar | chr1-115256529-T-A | NRAS | LIV | Q61L | Pathogenic |
| 18 | Clinvar | chr11-32456429-G-A | WT1 | BLY,TLY | Q155* | Pathogenic |
| 19 | Clinvar | chr11-48145375-A-C | PTPRJ | MAC,TES | Q276P | Pathogenic |
| 20 | Clinvar | chr11-66333834-G-A | CTSF | BLY,TLY | R217* | Pathogenic |
| 21 | Clinvar | chr1-179528845-C-T | NPHS2 | TLY | R168H | Pathogenic |
| 22 | Clinvar | chr12-112888162-G-C | PTPN11 | MAC | G60R | Pathogenic |
| 23 | Clinvar | chr12-25398284-C-G | KRAS | BLY | G12A | Pathogenic |
| 24 | Clinvar | chr12-6925407-C-T | CD4 | CRV | R265W | Pathogenic |
| 25 | Clinvar | chr12-76740563-C-T | BBS10 | BRE | G401E | Pathogenic |
| 26 | Clinvar | chr13-48916747-C-T | RB1 | SKN | Q93* | Pathogenic |
| 27 | Clinvar | chr14-51094875-C-T | ATL1 | BLY,TLY | R416C | Pathogenic |
| 28 | Clinvar | chr14-58899156-AG-A | KIAA0586 | BLY,TLY | R143* | Pathogenic |
| 29 | Clinvar | chr15-64698591-C-T | TRIP4 | TLY | R254* | Pathogenic |
| 30 | Clinvar | chr15-65313871-G-A | MTFMT | LIP | S209L | Pathogenic |
| 31 | Clinvar | chr15-66727483-G-A | MAP2K1 | TLY | D67N | Pathogenic |
| 32 | Clinvar | chr16-2376202-C-T | ABCA3 | MAC | R43H | Pathogenic |
| 33 | Clinvar | chr16-89351149-G-A | ANKRD11 | BLY,TLY | R601* | Pathogenic |
| 34 | Clinvar | chr17-56355397-G-A | MPO | LIP | A364V;A332V | Pathogenic |
| 35 | Clinvar | chr17-8139594-G-A | CTC1 | BLY,TLY | R287*;R252* | Pathogenic |
| 36 | Clinvar | chr19-1207057-T-G | STK11 | SKN | Y49D | Pathogenic |
| 37 | Clinvar | chr19-1220622-C-T | STK11 | BLY,TLY | Q214* | Pathogenic |
| 38 | Clinvar | chr19-33353427-C-T | SLC7A9 | LIP | A182T | Pathogenic |
| 39 | Clinvar | chr19-45412040-C-T | APOE | LIP | R163C;R189C | Pathogenic |
| 40 | Clinvar | chr19-45912070-A-G | CD3EAP | LIP | T284A;T282A | Pathogenic |
| 41 | Clinvar | chr21-44483184-A-G | CBS | LIP | I278T;I190T | Pathogenic |
| 42 | Clinvar | chr2-166012375-C-T | SCN3A | LIP | R357Q | Pathogenic |
| 43 | Clinvar | chr2-241737132-C-T | KIF1A | BLY,TLY | R13H | Pathogenic |
| 44 | Clinvar | chr2-44021647-G-A | DYNC2LI1 | BLY,TLY | W124*;W107* | Pathogenic |
| 45 | Clinvar | chr2-96953706-G-A | SNRNP200 | CRV | S1087L | Pathogenic |
| 46 | Clinvar | chr3-10331457-G-T | GHRL | SKN | L72M;L71M;L60M;L59M | Pathogenic |
| 47 | Clinvar | chr3-165548529-T-C | BCHE | BLY,TLY | D98G | Pathogenic |
| 48 | Clinvar | chr3-48621017-G-A | COL7A1 | SKN | P1458L | Pathogenic |
| 49 | Clinvar | chr4-1807803-G-A | FGFR3 | BLY,TLY | R623H;R622H;R509H;R621H | Pathogenic |
| 50 | Clinvar | chr4-187158034-G-A | KLKB1 | BLY,BRA,CRV,LIP,LIV,MAC,SKN,TES,TLY | S190N;S143N;S105N | Pathogenic |
| 51 | Clinvar | chr4-47954625-G-A | CNGA1 | BLY,TLY | R101*;R32* | Pathogenic |
| 52 | Clinvar | chr4-68606400-C-T | GNRHR | LIP | R262Q;T219T | Pathogenic |
| 53 | Clinvar | chr5-131930733-C-T | RAD50 | TLY | R656*;R517*;R595* | Pathogenic |
| 54 | Clinvar | chr5-150227998-C-T | IRGM | BRA,CRV | L105L | Pathogenic |
| 55 | Clinvar | chr5-176520243-G-A | FGFR4 | BLY,BRA,BRE,LIP,LIV,MAC,TES,TLY | G388R;G23R | Pathogenic |
| 56 | Clinvar | chr6-159403534-G-A | RSPH3 | BLY,TLY | R369*;R227*;R131*;R273* | Pathogenic |
| 57 | Clinvar | chr6-26091179-C-G | HFE | BRA,BRE | H63D;H40D | Pathogenic |
| 58 | Clinvar | chr8-90994994-G-A | NBN | BRE | R43* | Pathogenic |

**Table S10: List of number of variants in Sample A identified by type and by chromosome. A breakdown is provided by allele frequency ranges.**

| **** |
| --- |

**Table S11: List of 32 verified multi-allelic SNVs that were not otherwise identified and counted as positives. The observed VAF in pool A for each multi-allele variant is relative to reference. Thus, the individual variants VAFs may add up to greater than 100%.**

| \| **Multi-Allelic Variant** \| **chr** \| **Pos** \| **Ref** \| **Alt** \| **Observed VAF in pool A relative to ref** \| **Allele frequency in individual cell line** \| \| --- \| --- \| --- \| --- \| --- \| --- \| --- \| \| 1 \| chr1 \| 111783982 \| C \| T \| 0.0480 \| 0.460 \| \| 1 \| chr1 \| 111783982 \| C \| A \| 0.1900 \| 0.318,0.977,0.487 \| \| 2 \| chr1 \| 118165691 \| C \| G \| 0.0676 \| 0.475 \| \| 2 \| chr1 \| 118165691 \| C \| T \| 0.3197 \| 0.978,0.991,0.998,0.498 \| \| 3 \| chr1 \| 146714392 \| G \| T \| 0.5480 \| 0.995,0.487 \| \| 3 \| chr1 \| 146714392 \| G \| C \| 0.8073 \| 0.678,0.995,0.503,0.998,0.996,0.468 \| \| 4 \| chr1 \| 156594258 \| G \| C \| 0.0448 \| 0.204,0.386 \| \| 4 \| chr1 \| 156594258 \| G \| A \| 0.0637 \| 0.461 \| \| 5 \| chr2 \| 62449763 \| T \| G \| 0.0229 \| 0.212 \| \| 5 \| chr2 \| 62449763 \| T \| A \| 0.0429 \| 0.484 \| \| 6 \| chr2 \| 179325735 \| C \| G \| 0.0460 \| 0.426 \| \| 6 \| chr2 \| 179325735 \| C \| T \| 0.0932 \| 0.444,0.495 \| \| 7 \| chr2 \| 179605180 \| C \| A \| 0.0990 \| 0.629 \| \| 7 \| chr2 \| 179605180 \| C \| T \| 0.2353 \| 0.122,0.666,0.726,0.314 \| \| 8 \| chr5 \| 115394626 \| G \| T \| 0.1613 \| 0.453 \| \| 8 \| chr5 \| 115394626 \| G \| A \| 0.7113 \| 0.261,0.756,0.999,0.999,0.527,0.999,0.999,0.483 \| \| 9 \| chr6 \| 90402840 \| C \| A \| 0.6443 \| 0.993 \| \| 9 \| chr6 \| 90402840 \| C \| G \| 0.9193 \| 0.828,0.997,0.995,0.998,0.999,0.514,0.998,1,0.998 \| \| 10 \| chr7 \| 87160618 \| A \| T \| 0.2627 \| 0.662 \| \| 10 \| chr7 \| 87160618 \| A \| C \| 0.6197 \| 0.998,0.572,0.995,0.583,0.997,0.633,0.638 \| \| 11 \| chr7 \| 139724555 \| C \| T \| 0.2247 \| 0.996,0.371,0.335 \| \| 11 \| chr7 \| 139724555 \| C \| G \| 0.2810 \| 0.689,0.347,0.374,0.648 \| \| 12 \| chr8 \| 6389889 \| C \| A \| 0.1307 \| 0.321 \| \| 12 \| chr8 \| 6389889 \| C \| G \| 0.3020 \| 0.314,0.481,0.995,0.306,0.367 \| \| 13 \| chr8 \| 67796120 \| C \| T \| 0.0443 \| 0.167,0.187 \| \| 13 \| chr8 \| 67796120 \| C \| G \| 0.0883 \| 0.512,0.35 \| \| 14 \| chr9 \| 5921844 \| G \| T \| 0.0383 \| 0.484 \| \| 14 \| chr9 \| 5921844 \| G \| A \| 0.0511 \| 0.201,0.235 \| \| 15 \| chr9 \| 130536484 \| C \| T \| 0.0257 \| 0.305 \| \| 15 \| chr9 \| 130536484 \| C \| A \| 0.1210 \| 0.843 \| \| 16 \| chr10 \| 27692279 \| A \| G \| 0.0834 \| 0.436 \| \| 16 \| chr10 \| 27692279 \| A \| C \| 0.3073 \| 0.65,0.991,0.437,0.454,0.432,0.462 \| \| 17 \| chr10 \| 123970722 \| T \| C \| 0.0574 \| 0.550 \| \| 17 \| chr10 \| 123970722 \| T \| A \| 0.1613 \| 0.334,0.497,0.432 \| \| 18 \| chr11 \| 5221645 \| C \| A \| 0.5953 \| 0.998,0.989,0.471 \| \| 18 \| chr11 \| 5221645 \| C \| G \| 0.6713 \| 0.63,0.99,0.46,0.996,0.44 \| \| 19 \| chr11 \| 5989415 \| C \| A \| 0.4053 \| 0.971,0.994 \| \| 19 \| chr11 \| 5989415 \| C \| T \| 0.5440 \| 0.995,0.998,0.51,0.995,0.44 \| \| 20 \| chr11 \| 6579106 \| C \| T \| 0.0598 \| 0.443 \| \| 20 \| chr11 \| 6579106 \| C \| A \| 0.2720 \| 0.608,0.475,0.988,0.475 \| \| 21 \| chr12 \| 6730253 \| G \| C \| 0.0306 \| 0.133 \| \| 21 \| chr12 \| 6730253 \| G \| A \| 0.0419 \| 0.17,0.234 \| \| 22 \| chr12 \| 104048454 \| C \| G \| 0.0296 \| 0.292 \| \| 22 \| chr12 \| 104048454 \| C \| A \| 0.0727 \| 0.989 \| \| 23 \| chr14 \| 73727509 \| T \| C \| 0.0270 \| 0.238 \| \| 23 \| chr14 \| 73727509 \| T \| G \| 0.2217 \| 0.983,0.648,0.477 \| \| 24 \| chr14 \| 105180706 \| A \| G \| 0.3430 \| 0.227,0.411,0.996 \| \| 24 \| chr14 \| 105180706 \| A \| C \| 0.5990 \| 0.994,0.485,0.996,0.995,0.993 \| \| 25 \| chr16 \| 57937856 \| G \| T \| 0.3703 \| 0.335 \| \| 25 \| chr16 \| 57937856 \| G \| C \| 0.8437 \| 0.793,0.996,0.999,0.998,0.996,0.643,0.996 \| \| 26 \| chr16 \| 89265101 \| C \| A \| 0.0449 \| 0.314 \| \| 26 \| chr16 \| 89265101 \| C \| T \| 0.2313 \| 0.568,0.538,0.303,0.444 \| \| 27 \| chr17 \| 33806812 \| C \| A \| 0.0675 \| 0.484 \| \| 27 \| chr17 \| 33806812 \| C \| T \| 0.2040 \| 0.438,0.613,0.606 \| \| 28 \| chr17 \| 48356260 \| G \| A \| 0.4290 \| 0.997 \| \| 28 \| chr17 \| 48356260 \| G \| C \| 0.5853 \| 0.391,0.998,0.992,0.454 \| \| 29 \| chr17 \| 66596463 \| C \| G \| 0.0214 \| 0.167 \| \| 29 \| chr17 \| 66596463 \| C \| T \| 0.1320 \| 0.587 \| \| 30 \| chr18 \| 34647323 \| T \| A \| 0.1052 \| 0.2,0.645 \| \| 30 \| chr18 \| 34647323 \| T \| G \| 0.1910 \| 0.801,0.466 \| \| 31 \| chr19 \| 52249672 \| A \| G \| 0.1443 \| 0.732,0.479 \| \| 31 \| chr19 \| 52249672 \| A \| C \| 0.2400 \| 0.631,0.335,0.999 \| \| 32 \| chr21 \| 43169357 \| C \| T \| 0.0801 \| 0.548,0.215 \| \| 32 \| chr21 \| 43169357 \| C \| G \| 0.1623 \| 0.443,0.999,0.462 \| |  |
| --- | --- | --- | --- | --- | --- | --- | --- | --- | --- | --- | --- | --- | --- | --- | --- | --- | --- | --- | --- | --- | --- | --- | --- | --- | --- | --- | --- | --- | --- | --- | --- | --- | --- | --- | --- | --- | --- | --- | --- | --- | --- | --- | --- | --- | --- | --- | --- | --- | --- | --- | --- | --- | --- | --- | --- | --- | --- | --- | --- | --- | --- | --- | --- | --- | --- | --- | --- | --- | --- | --- | --- | --- | --- | --- | --- | --- | --- | --- | --- | --- | --- | --- | --- | --- | --- | --- | --- | --- | --- | --- | --- | --- | --- | --- | --- | --- | --- | --- | --- | --- | --- | --- | --- | --- | --- | --- | --- | --- | --- | --- | --- | --- | --- | --- | --- | --- | --- | --- | --- | --- | --- | --- | --- | --- | --- | --- | --- | --- | --- | --- | --- | --- | --- | --- | --- | --- | --- | --- | --- | --- | --- | --- | --- | --- | --- | --- | --- | --- | --- | --- | --- | --- | --- | --- | --- | --- | --- | --- | --- | --- | --- | --- | --- | --- | --- | --- | --- | --- | --- | --- | --- | --- | --- | --- | --- | --- | --- | --- | --- | --- | --- | --- | --- | --- | --- | --- | --- | --- | --- | --- | --- | --- | --- | --- | --- | --- | --- | --- | --- | --- | --- | --- | --- | --- | --- | --- | --- | --- | --- | --- | --- | --- | --- | --- | --- | --- | --- | --- | --- | --- | --- | --- | --- | --- | --- | --- | --- | --- | --- | --- | --- | --- | --- | --- | --- | --- | --- | --- | --- | --- | --- | --- | --- | --- | --- | --- | --- | --- | --- | --- | --- | --- | --- | --- | --- | --- | --- | --- | --- | --- | --- | --- | --- | --- | --- | --- | --- | --- | --- | --- | --- | --- | --- | --- | --- | --- | --- | --- | --- | --- | --- | --- | --- | --- | --- | --- | --- | --- | --- | --- | --- | --- | --- | --- | --- | --- | --- | --- | --- | --- | --- | --- | --- | --- | --- | --- | --- | --- | --- | --- | --- | --- | --- | --- | --- | --- | --- | --- | --- | --- | --- | --- | --- | --- | --- | --- | --- | --- | --- | --- | --- | --- | --- | --- | --- | --- | --- | --- | --- | --- | --- | --- | --- | --- | --- | --- | --- | --- | --- | --- | --- | --- | --- | --- | --- | --- | --- | --- | --- | --- | --- | --- | --- | --- | --- | --- | --- | --- | --- | --- | --- | --- | --- | --- | --- | --- | --- | --- | --- | --- | --- | --- | --- | --- | --- | --- | --- | --- | --- | --- | --- | --- | --- | --- | --- | --- | --- | --- | --- | --- | --- | --- | --- | --- | --- | --- | --- | --- | --- | --- | --- | --- | --- | --- | --- | --- | --- | --- | --- | --- | --- | --- | --- | --- | --- | --- | --- | --- | --- | --- | --- | --- | --- | --- | --- | --- | --- | --- | --- | --- | --- | --- | --- | --- | --- | --- | --- | --- | --- | --- | --- | --- | --- | --- | --- | --- |

**Table S12: Basic information of the ddPCR assays and their variant class. LF is for Low frequency.**

| **BioRad_assay_ID** | **COSMIC ID** | **Gene** | **Chr** | **Pos** | **Strand** | **Ref** | **Alt** | **Class** |
| --- | --- | --- | --- | --- | --- | --- | --- | --- |
| dHsaMDS629388267 |  | TUT1 | chr11 | 62359060 | - | AG | A | Class 1 Indel |
| dHsaMDS452271618 |  | NR2E3 | chr15 | 72105928 | + | AC | A | Class 1 Indel |
| dHsaMDS499654470 |  | HSH2D | chr19 | 16268207 | + | TA | T | Class 1 Indel |
| dHsaMDS642431706 |  | TIGD6 | chr5 | 149374879 | - | CT | C | Class 1 Indel |
| dHsaMDS611432465 |  | CYFIP2 | chr5 | 156721863 | + | T | TC | Class 1 Indel |
| dHsaMDS474404020 |  | XKR5 | chr8 | 6673377 | - | CA | C | Class 1 Indel |
| dHsaMDS447280785 |  | UBXN8 | chr8 | 30620840 | + | G | GT | Class 1 Indel |
| dHsaMDS411642066 | COSM6978756 | GATA3 | chr10 | 8111513 | + | T | TG | Class 1 Indel COSMIC |
| dHsaMDS131371839 |  | DENND4B | chr1 | 153904867 | - | AG | A | Class 1 Indel LF |
| dHsaMDS389006411 |  | EHF | chr11 | 34654157 | + | A | AG | Class 1 Indel LF |
| dHsaMDS999815596 |  | CCDC81 | chr11 | 86108740 | + | AG | A | Class 1 Indel LF |
| dHsaMDS341541009 |  | KIAA0586 | chr14 | 58899156 | + | AG | A | Class 1 Indel LF |
| dHsaMDS171857374 |  | AP4E1 | chr15 | 51285689 | + | TG | T | Class 1 Indel LF |
| dHsaMDS822591953 |  | TBC1D10B | chr16 | 30369576 | - | G | GA | Class 1 Indel LF |
| dHsaMDS398032227 |  | MYO5B | chr18 | 47432834 | - | GC | G | Class 1 Indel LF |
| dHsaMDS980439245 |  | RBM6 | chr3 | 50005040 | + | CG | C | Class 1 Indel LF |
| dHsaMDS233908974 |  | SLC12A8 | chr3 | 124829073 | - | TG | T | Class 1 Indel LF |
| dHsaMDS736870949 |  | LYAR | chr4 | 4276251 | - | CT | C | Class 1 Indel LF |
| dHsaMDS264901159 |  | STXBP5 | chr6 | 147646187 | + | TA | T | Class 1 Indel LF |
| dHsaMDS139552125 |  | DNAH11 | chr7 | 21726823 | + | C | CT | Class 1 Indel LF |
| dHsaMDS804679022 |  | TAF6 | chr7 | 99711868 | - | GC | G | Class 1 Indel LF |
| dHsaMDS405472888 |  | ASB15 | chr7 | 123267185 | + | CG | C | Class 1 Indel LF |
| dHsaMDS169941228 |  | BEST4 | chr1 | 45250401 | - | CCTT | C | Class 1 Indel LF & Complex |
| dHsaMDS937435255 |  | KCTD3 | chr1 | 215792630 | + | C | CTAG | Class 1 Indel LF & Complex |
| dHsaMDS144716159 |  | DNAH14 | chr1 | 225380561 | + | CAAAG | C | Class 1 Indel LF & Complex |
| dHsaMDS214379238 |  | GANAB | chr11 | 62400542 | - | C | CAG | Class 1 Indel LF & Complex |
| dHsaMDS560034215 |  | CLPB | chr11 | 72145489 | - | T | TAAA | Class 1 Indel LF & Complex |
| dHsaMDS147651305 |  | CDIP1 | chr16 | 4563725 | - | CAT | C | Class 1 Indel LF & Complex |
| dHsaMDS767394512 |  | OR4D1 | chr17 | 56232679 | + | CCA | C | Class 1 Indel LF & Complex |
| dHsaMDS378458581 |  | ABCA10 | chr17 | 67145190 | - | TGA | T | Class 1 Indel LF & Complex |
| dHsaMDS225933689 |  | ABCA10 | chr17 | 67190536 | - | AACAG | A | Class 1 Indel LF & Complex |
| dHsaMDS704022664 |  | DEFB126 | chr20 | 126155 | + | GCAAA | G | Class 1 Indel LF & Complex |
| dHsaMDS206778655 |  | SAMHD1 | chr20 | 35555633 | - | CAT | C | Class 1 Indel LF & Complex |
| dHsaMDS908376548 |  | CACNA1D | chr3 | 53842691 | + | CCTT | C | Class 1 Indel LF & Complex |
| dHsaMDS848698758 |  | CCDC37 | chr3 | 126142433 | + | CGGA | C | Class 1 Indel LF & Complex |
| dHsaMDS607382350 |  | AP3B1 | chr5 | 77311339 | - | ACAG | A | Class 1 Indel LF & Complex |
| dHsaMDS928556350 |  | AP3B1 | chr5 | 77396835 | - | TTTC | T | Class 1 Indel LF & Complex |
| dHsaMDS248048714 |  | KIF6 | chr6 | 39387731 | - | ACTT | A | Class 1 Indel LF & Complex |
| dHsaMDS485172760 |  | KIAA1009 | chr6 | 84896313 | - | TTTC | T | Class 1 Indel LF & Complex |
| dHsaMDS139526943 |  | CEP85L | chr6 | 118812810 | - | ATGT | A | Class 1 Indel LF & Complex |
| dHsaMDS853307825 |  | ECHDC1 | chr6 | 127611132 | - | GAACA | G | Class 1 Indel LF & Complex |
| dHsaMDS508765899 |  | TAS2R60 | chr7 | 143140930 | + | GTCT | G | Class 1 Indel LF & Complex |
| dHsaMDS896318547 |  | OR5M1 | chr11 | 56380546 | - | CCAGA | C | Class 1 Indel-Complex |
| dHsaMDS131540988 |  | LRRC49 | chr15 | 71276480 | + | GCAA | G | Class 1 Indel-Complex |
| dHsaMDS409973570 |  | KANK3 | chr19 | 8389892 | - | GTCC | G | Class 1 Indel-Complex |
| dHsaMDS560635842 |  | KCNA7 | chr19 | 49573362 | - | TGGA | T | Class 1 Indel-Complex |
| dHsaMDS255267516 |  | SLC41A3 | chr3 | 125725268 | - | TAACA | T | Class 1 Indel-Complex |
| dHsaMDS960447891 |  | VEGFC | chr4 | 177605081 | - | CTCA | C | Class 1 Indel-Complex |
| dHsaMDS439788935 |  | NPVF | chr7 | 25266569 | - | TTAA | T | Class 1 Indel-Complex |
| dHsaMDS460669204 |  | ARHGAP39 | chr8 | 145773341 | - | TCTG | T | Class 1 Indel-Complex |
| dHsaMDS500788702 | COSM5021001 | MTOR | chr1 | 11184593 | - | A | G | Class 1 SNV COSMIC |
| dHsaMDS864647843 | COSM4142152 | MTOR | chr1 | 11288758 | - | G | A | Class 1 SNV COSMIC |
| dHsaMDS227766171 | COSM6494338 | CSF3R | chr1 | 36934805 | - | C | G | Class 1 SNV COSMIC |
| dHsaMDS456016425 | COSM5020949 | MPL | chr1 | 43804340 | + | G | A | Class 1 SNV COSMIC |
| dHsaMDS988391040 | COSM5019688 | MPL | chr1 | 43805240 | + | A | G | Class 1 SNV COSMIC |
| dHsaMDS835373540 | COSM3751352 | JAK1 | chr1 | 65311262 | - | G | A | Class 1 SNV COSMIC |
| dHsaMDS581135936 | COSM5019756 | JAK1 | chr1 | 65312342 | - | G | A | Class 1 SNV COSMIC |
| dHsaMDS817387298 | COSM3996788 | NOTCH2 | chr1 | 120458924 | - | G | A | Class 1 SNV COSMIC |
| dHsaMDS923559197 | COSM6494259 | NOTCH2 | chr1 | 120483244 | - | C | T | Class 1 SNV COSMIC |
| dHsaMDS833188765 | COSM4144894 | GATA3 | chr10 | 8100647 | + | C | T | Class 1 SNV COSMIC |
| dHsaMDS720650892 | COSM5983421 | RET | chr10 | 43606687 | + | A | G | Class 1 SNV COSMIC |
| dHsaMDS742565194 | COSM1957202 | RET | chr10 | 43608351 | + | G | A | Class 1 SNV COSMIC |
| dHsaMDS246540935 | COSM14078 | PTEN | chr10 | 89717769 | + | TA | T | Class 1 SNV COSMIC |
| dHsaMDS175410602 | COSM2110318 | ATM | chr11 | 108098524 | + | C | T | Class 1 SNV COSMIC |
| dHsaMDS788764010 | COSM4985271 | ATM | chr11 | 108106443 | + | T | A | Class 1 SNV COSMIC |
| dHsaMDS366984534 | COSM6493967 | ATM | chr11 | 108119770 | + | C | G | Class 1 SNV COSMIC |
| dHsaMDS781316075 | COSM4590264 | ATM | chr11 | 108183167 | + | A | G | Class 1 SNV COSMIC |
| dHsaMDS579057705 | COSM6494016 | CBL | chr11 | 119170362 | + | C | T | Class 1 SNV COSMIC |
| dHsaMDS414392380 | COSM3746300 | COL2A1 | chr12 | 48367976 | - | C | T | Class 1 SNV COSMIC |
| dHsaMDS977927486 | COSM6986742 | KMT2D | chr12 | 49418488 | - | G | C | Class 1 SNV COSMIC |
| dHsaMDS961730427 | COSM1361971 | KMT2D | chr12 | 49425037 | - | C | T | Class 1 SNV COSMIC |
| dHsaMDS508359418 | COSM3998819 | MLL2 | chr12 | 49425978 | - | T | C | Class 1 SNV COSMIC |
| dHsaMDS981953983 | COSM6984176 | MLL2 | chr12 | 49426326 | - | G | A | Class 1 SNV COSMIC |
| dHsaMDS248273268 | COSM2007123 | MLL2 | chr12 | 49435269 | - | C | T | Class 1 SNV COSMIC |
| dHsaMDS925581600 | COSM6915278 | MLL2 | chr12 | 49447772 | - | G | T | Class 1 SNV COSMIC |
| dHsaMDS904118198 | COSM1677074 | ERBB3 | chr12 | 56481390 | + | C | T | Class 1 SNV COSMIC |
| dHsaMDS660235038 | COSM6494130 | SH2B3 | chr12 | 111884608 | + | T | C | Class 1 SNV COSMIC |
| dHsaMDS156779143 | COSM5020679 | HNF1A | chr12 | 121416864 | + | C | T | Class 1 SNV COSMIC |
| dHsaMDS102452487 | COSM3931546 | HNF1A | chr12 | 121435342 | + | C | T | Class 1 SNV COSMIC |
| dHsaMDS947262496 | COSM4984989 | HNF1A | chr12 | 121435427 | + | G | A | Class 1 SNV COSMIC |
| dHsaMDS372585399 | COSM6930961 | POLE | chr12 | 133212582 | - | G | A | Class 1 SNV COSMIC |
| dHsaMDS243335575 | COSM2001711 | POLE | chr12 | 133241895 | - | G | A | Class 1 SNV COSMIC |
| dHsaMDS833043652 | COSM2001749 | POLE | chr12 | 133248860 | - | G | A | Class 1 SNV COSMIC |
| dHsaMDS480557847 | COSM19545 | RB1 | chr13 | 48916747 | + | C | T | Class 1 SNV COSMIC |
| dHsaMDS623870489 | COSM5019393 | FOXA1 | chr14 | 38060646 | - | C | T | Class 1 SNV COSMIC |
| dHsaMDS469982939 | COSM5019750 | HIF1A | chr14 | 62207557 | + | C | T | Class 1 SNV COSMIC |
| dHsaMDS768634536 | COSM3754139 | TSHR | chr14 | 81562998 | + | T | C | Class 1 SNV COSMIC |
| dHsaMDS883891064 | COSM143917 | AXIN1 | chr16 | 348042 | - | C | A | Class 1 SNV COSMIC |
| dHsaMDS461012556 | COSM4606359 | TSC2 | chr16 | 2110795 | + | G | A | Class 1 SNV COSMIC |
| dHsaMDS630495205 | COSM4985532 | TSC2 | chr16 | 2133798 | + | G | A | Class 1 SNV COSMIC |
| dHsaMDS557432550 | COSM6981152 | TSC2 | chr16 | 2136298 | + | G | T | Class 1 SNV COSMIC |
| dHsaMDS866006981 | COSM5019155 | CREBBP | chr16 | 3779594 | - | C | T | Class 1 SNV COSMIC |
| dHsaMDS426546017 | COSM3188712 | STAT3 | chr17 | 40490777 | - | G | A | Class 1 SNV COSMIC |
| dHsaMDS705773004 | COSM4985686 | BRCA1 | chr17 | 41222975 | - | C | T | Class 1 SNV COSMIC |
| dHsaMDS385989876 | COSM148278 | BRCA1 | chr17 | 41244936 | - | G | A | Class 1 SNV COSMIC |
| dHsaMDS257400736 | COSM3755566 | BRCA1 | chr17 | 41245237 | - | A | G | Class 1 SNV COSMIC |
| dHsaMDS241968756 | COSM6908580 | RNF43 | chr17 | 56492800 | - | T | C | Class 1 SNV COSMIC |
| dHsaMDS891627970 | COSM148316 | CD79B | chr17 | 62007498 | - | A | G | Class 1 SNV COSMIC |
| dHsaMDS635666928 | COSM6494525 | SETBP1 | chr18 | 42529996 | + | G | C | Class 1 SNV COSMIC |
| dHsaMDS903883380 | COSM6494528 | SETBP1 | chr18 | 42532923 | + | T | C | Class 1 SNV COSMIC |
| dHsaMDS633378125 | COSM6494530 | SETBP1 | chr18 | 42533130 | + | A | G | Class 1 SNV COSMIC |
| dHsaMDS851259804 | COSM6921987 | SMAD2 | chr18 | 45375015 | - | C | T | Class 1 SNV COSMIC |
| dHsaMDS602842844 | COSM27023 | STK11 | chr19 | 1207057 | + | T | G | Class 1 SNV COSMIC |
| dHsaMDS678545025 | COSM5020491 | MAP2K2 | chr19 | 4117528 | - | G | A | Class 1 SNV COSMIC |
| dHsaMDS358727085 | COSM3756293 | SMARCA4 | chr19 | 11169514 | + | C | T | Class 1 SNV COSMIC |
| dHsaMDS626283823 | COSM3286487 | CD79A | chr19 | 42383236 | + | G | A | Class 1 SNV COSMIC |
| dHsaMDS435356956 | COSM3756832 | CIC | chr19 | 42795554 | + | T | C | Class 1 SNV COSMIC |
| dHsaMDS617089985 | COSM4001621 | DNMT3A | chr2 | 25469502 | - | C | T | Class 1 SNV COSMIC |
| dHsaMDS154473017 | COSM307361 | DNMT3A | chr2 | 25523096 | - | T | G | Class 1 SNV COSMIC |
| dHsaMDS592363814 | COSM148824 | ALK | chr2 | 29449819 | - | C | T | Class 1 SNV COSMIC |
| dHsaMDS159244617 | COSM3186058 | FBXO11 | chr2 | 48036361 | - | T | C | Class 1 SNV COSMIC |
| dHsaMDS638144109 | COSM5019588 | LRP1B | chr2 | 141457985 | - | T | A | Class 1 SNV COSMIC |
| dHsaMDS486734853 | COSM4964776 | LRP1B | chr2 | 141751592 | - | G | A | Class 1 SNV COSMIC |
| dHsaMDS766279517 | COSM6494781 | SF3B1 | chr2 | 198281489 | - | T | C | Class 1 SNV COSMIC |
| dHsaMDS211984362 | COSM1741220 | IDH1 | chr2 | 209113192 | - | G | A | Class 1 SNV COSMIC |
| dHsaMDS508465523 | COSM96923 | IDH1 | chr2 | 209113296 | - | C | T | Class 1 SNV COSMIC |
| dHsaMDS138703644 | COSM6914247 | ERBB4 | chr2 | 212587142 | - | C | T | Class 1 SNV COSMIC |
| dHsaMDS620732908 | COSM6946807 | SRC | chr20 | 36030939 | + | G | C | Class 1 SNV COSMIC |
| dHsaMDS747764993 | COSM4134539 | PLCG1 | chr20 | 39792063 | + | A | G | Class 1 SNV COSMIC |
| dHsaMDS223830778 | COSM5020737 | ERG | chr21 | 39775468 | - | G | A | Class 1 SNV COSMIC |
| dHsaMDS428380642 | COSM53293 | SMARCB1 | chr22 | 24167513 | + | G | A | Class 1 SNV COSMIC |
| dHsaMDS164001412 | COSM5009621 | EP300 | chr22 | 41548008 | + | A | G | Class 1 SNV COSMIC |
| dHsaMDS538013266 | COSM1131469 | MLH1 | chr3 | 37053568 | + | A | G | Class 1 SNV COSMIC |
| dHsaMDS397470176 | COSM149376 | SETD2 | chr3 | 47125385 | - | G | A | Class 1 SNV COSMIC |
| dHsaMDS824887592 | COSM4002743 | PBRM1 | chr3 | 52584787 | - | T | C | Class 1 SNV COSMIC |
| dHsaMDS472901709 | COSM6494853 | CBLB | chr3 | 105422844 | - | C | T | Class 1 SNV COSMIC |
| dHsaMDS599597924 | COSM5019736 | GATA2 | chr3 | 128200072 | - | C | T | Class 1 SNV COSMIC |
| dHsaMDS727571184 | COSM6494874 | GATA2 | chr3 | 128204877 | - | C | G | Class 1 SNV COSMIC |
| dHsaMDS423277485 | COSM149487 | ATR | chr3 | 142281612 | - | A | G | Class 1 SNV COSMIC |
| dHsaMDS822378744 | COSM5019247 | PIK3CA | chr3 | 178927410 | + | A | G | Class 1 SNV COSMIC |
| dHsaMDS664516678 | COSM1428696 | FGFR3 | chr4 | 1803704 | + | T | C | Class 1 SNV COSMIC |
| dHsaMDS875472833 | COSM327093 | FGFR3 | chr4 | 1807803 | + | G | A | Class 1 SNV COSMIC |
| dHsaMDS605241459 | COSM6927286 | PDGFRA | chr4 | 55127448 | + | G | A | Class 1 SNV COSMIC |
| dHsaMDS978773791 | COSM4416371 | PDGFRA | chr4 | 55130078 | + | T | C | Class 1 SNV COSMIC |
| dHsaMDS616547679 | COSM1666925 | PDGFRA | chr4 | 55133726 | + | T | G | Class 1 SNV COSMIC |
| dHsaMDS385711705 | COSM5008347 | PDGFRA | chr4 | 55139771 | + | T | C | Class 1 SNV COSMIC |
| dHsaMDS795155993 | COSM4417622 | PDGFRA | chr4 | 55143577 | + | G | A | Class 1 SNV COSMIC |
| dHsaMDS2511598 | COSM1325 | KIT | chr4 | 55602765 | + | G | C | Class 1 SNV COSMIC |
| dHsaMDS733185050 | COSM4158786 | FAT4 | chr4 | 126336703 | + | T | C | Class 1 SNV COSMIC |
| dHsaMDS164898894 | COSM4416273 | FAT1 | chr4 | 187630590 | - | G | A | Class 1 SNV COSMIC |
| dHsaMDS830701339 | COSM5020092 | TERT | chr5 | 1253918 | - | C | T | Class 1 SNV COSMIC |
| dHsaMDS160525354 | COSM3761218 | IL7R | chr5 | 35871273 | + | C | T | Class 1 SNV COSMIC |
| dHsaMDS926019402 | COSM4407349 | PIK3R1 | chr5 | 67522722 | + | C | T | Class 1 SNV COSMIC |
| dHsaMDS257796315 | COSM33672 | APC | chr5 | 112175770 | + | G | A | Class 1 SNV COSMIC |
| dHsaMDS581965709 | COSM5020477 | CSF1R | chr5 | 149450132 | - | T | C | Class 1 SNV COSMIC |
| dHsaMDS574960722 | COSM6942813 | ESR1 | chr6 | 152265522 | + | G | C | Class 1 SNV COSMIC |
| dHsaMDS751087126 | COSM3761556 | ESR1 | chr6 | 152420095 | + | G | A | Class 1 SNV COSMIC |
| dHsaMDS377562075 | COSM5019288 | CARD11 | chr7 | 2957005 | - | T | C | Class 1 SNV COSMIC |
| dHsaMDS214371112 | COSM4162140 | CARD11 | chr7 | 2976767 | - | G | A | Class 1 SNV COSMIC |
| dHsaMDS680106736 | COSM5019424 | CARD11 | chr7 | 2985586 | - | C | G | Class 1 SNV COSMIC |
| dHsaMDS865335490 | COSM5019979 | EGFR | chr7 | 55233089 | + | C | T | Class 1 SNV COSMIC |
| dHsaMDS764936753 | COSM1451600 | EGFR | chr7 | 55249063 | + | G | A | Class 1 SNV COSMIC |
| dHsaMDS657874824 | COSM3762773 | EGFR | chr7 | 55268916 | + | C | T | Class 1 SNV COSMIC |
| dHsaMDS123432194 | COSM5020205 | MET | chr7 | 116340269 | + | C | T | Class 1 SNV COSMIC |
| dHsaMDS670919316 | COSM4419559 | MET | chr7 | 116397572 | + | A | G | Class 1 SNV COSMIC |
| dHsaMDS599414013 | COSM150377 | MET | chr7 | 116435768 | + | C | T | Class 1 SNV COSMIC |
| dHsaMDS491244810 | COSM3750133 | PREX2 | chr8 | 69058536 | + | C | T | Class 1 SNV COSMIC |
| dHsaMDS714778374 | COSM87165 | PAX5 | chr9 | 36840623 | - | G | A | Class 1 SNV COSMIC |
| dHsaMDS678705699 | COSM5019121 | ABL1 | chr9 | 133761001 | + | A | G | Class 1 SNV COSMIC |
| dHsaMDS337033655 | COSM1461158 | NOTCH1 | chr9 | 139397707 | - | G | A | Class 1 SNV COSMIC |
| dHsaMDS685586293 | COSM6950647 | NOTCH1 | chr9 | 139405721 | - | C | T | Class 1 SNV COSMIC |
| dHsaMDS625815438 | COSM3763787 | NOTCH1 | chr9 | 139407932 | - | A | G | Class 1 SNV COSMIC |
| dHsaMDS310100512 | COSM3216118 | NOTCH1 | chr9 | 139418189 | - | C | T | Class 1 SNV COSMIC |
| dHsaMDS231886687 | COSM1756050 | NOTCH1 | chr9 | 139418260 | - | A | G | Class 1 SNV COSMIC |
| dHsaMDS126760902 |  | RAD54L | chr1 | 46724370 | + | C | T | Class 1 SNV LF unique to a cell line |
| dHsaMDS930035853 |  | BMS1 | chr10 | 43292647 | + | A | G | Class 1 SNV LF unique to a cell line |
| dHsaMDS251300278 |  | PKD2L1 | chr10 | 102048208 | - | G | T | Class 1 SNV LF unique to a cell line |
| dHsaMDS634084281 |  | PDE3B | chr11 | 14666116 | + | C | A | Class 1 SNV LF unique to a cell line |
| dHsaMDS133071749 |  | CHRM1 | chr11 | 62678306 | - | G | T | Class 1 SNV LF unique to a cell line |
| dHsaMDS254514854 |  | C1S | chr12 | 7173913 | + | T | C | Class 1 SNV LF unique to a cell line |
| dHsaMDS742015309 |  | ETV6 | chr12 | 12043904 | + | G | A | Class 1 SNV LF unique to a cell line |
| dHsaMDS992782939 |  | MDGA2 | chr14 | 47613364 | - | G | T | Class 1 SNV LF unique to a cell line |
| dHsaMDS607403518 |  | PPP1R14D | chr15 | 41120763 | - | G | T | Class 1 SNV LF unique to a cell line |
| dHsaMDS610490889 |  | SLC28A2 | chr15 | 45556951 | + | G | T | Class 1 SNV LF unique to a cell line |
| dHsaMDS638032362 |  | NDRG4 | chr16 | 58540875 | + | G | A | Class 1 SNV LF unique to a cell line |
| dHsaMDS340043646 |  | CTCF | chr16 | 67645069 | + | G | T | Class 1 SNV LF unique to a cell line |
| dHsaMDS182929552 |  | SF3B3 | chr16 | 70605093 | + | C | T | Class 1 SNV LF unique to a cell line |
| dHsaMDS715950632 |  | ERN1 | chr17 | 62137930 | - | G | A | Class 1 SNV LF unique to a cell line |
| dHsaMDS860373034 |  | SAP30BP | chr17 | 73689541 | + | C | T | Class 1 SNV LF unique to a cell line |
| dHsaMDS682715369 |  | RAC3 | chr17 | 79990825 | + | C | T | Class 1 SNV LF unique to a cell line |
| dHsaMDS591961210 |  | PTPRM | chr18 | 8253395 | + | T | A | Class 1 SNV LF unique to a cell line |
| dHsaMDS252586812 |  | CD22 | chr19 | 35828831 | + | G | A | Class 1 SNV LF unique to a cell line |
| dHsaMDS797235784 |  | PRKD2 | chr19 | 47200453 | - | G | A | Class 1 SNV LF unique to a cell line |
| dHsaMDS143471457 |  | PREB | chr2 | 27356487 | - | C | T | Class 1 SNV LF unique to a cell line |
| dHsaMDS912610722 |  | PTCD3 | chr2 | 86359481 | + | A | G | Class 1 SNV LF unique to a cell line |
| dHsaMDS975542950 |  | ANKRD44 | chr2 | 198001319 | - | A | G | Class 1 SNV LF unique to a cell line |
| dHsaMDS232084453 |  | LAMP5 | chr20 | 9510297 | + | T | C | Class 1 SNV LF unique to a cell line |
| dHsaMDS261909011 |  | SOGA1 | chr20 | 35467826 | - | G | A | Class 1 SNV LF unique to a cell line |
| dHsaMDS868878001 |  | AP000304.12 | chr21 | 34997018 | - | C | T | Class 1 SNV LF unique to a cell line |
| dHsaMDS378435377 |  | KCTD17 | chr22 | 37458586 | + | C | T | Class 1 SNV LF unique to a cell line |
| dHsaMDS717481584 |  | NEK10 | chr3 | 27335120 | - | T | C | Class 1 SNV LF unique to a cell line |
| dHsaMDS821892659 |  | SH3BP2 | chr4 | 2834081 | + | G | A | Class 1 SNV LF unique to a cell line |
| dHsaMDS750279601 |  | SEL1L3 | chr4 | 25834686 | - | A | G | Class 1 SNV LF unique to a cell line |
| dHsaMDS641868522 |  | ELOVL7 | chr5 | 60063722 | - | C | A | Class 1 SNV LF unique to a cell line |
| dHsaMDS265567984 |  | IQGAP2 | chr5 | 75969360 | + | G | T | Class 1 SNV LF unique to a cell line |
| dHsaMDS110597252 |  | PDE8B | chr5 | 76700611 | + | G | A | Class 1 SNV LF unique to a cell line |
| dHsaMDS974239386 |  | ADAMTS19 | chr5 | 128844838 | + | A | G | Class 1 SNV LF unique to a cell line |
| dHsaMDS885067518 |  | PPP2R2B | chr5 | 146070789 | - | G | A | Class 1 SNV LF unique to a cell line |
| dHsaMDS570755749 |  | UBE3D | chr6 | 83667045 | - | C | T | Class 1 SNV LF unique to a cell line |
| dHsaMDS452213092 |  | CROT | chr7 | 87011283 | + | A | T | Class 1 SNV LF unique to a cell line |
| dHsaMDS525770934 |  | PRKAG2 | chr7 | 151262959 | - | G | A | Class 1 SNV LF unique to a cell line |
| dHsaMDS687646292 |  | BMP1 | chr8 | 22037971 | + | G | A | Class 1 SNV LF unique to a cell line |
| dHsaMDS165679413 |  | ADAMDEC1 | chr8 | 24256052 | + | C | T | Class 1 SNV LF unique to a cell line |
| dHsaMDS689006907 |  | CLVS1 | chr8 | 62212402 | + | C | T | Class 1 SNV LF unique to a cell line |
| dHsaMDS183293602 |  | SH3D21 | chr1 | 36773127 | + | G | A | Class 1 SNV LF unique to BLY |
| dHsaMDS564822559 |  | PRKG1 | chr10 | 53822301 | + | A | G | Class 1 SNV LF unique to BLY |
| dHsaMDS460649839 |  | SYNPO2L | chr10 | 75413991 | - | G | A | Class 1 SNV LF unique to BLY |
| dHsaMDS177039574 |  | FGF14 | chr13 | 102379088 | - | A | G | Class 1 SNV LF unique to BLY |
| dHsaMDS720472505 |  | ANKS3 | chr16 | 4751124 | - | A | G | Class 1 SNV LF unique to BLY |
| dHsaMDS893036422 |  | ALOX15 | chr17 | 4535301 | - | G | A | Class 1 SNV LF unique to BLY |
| dHsaMDS848108741 |  | NLK | chr17 | 26518107 | + | C | A | Class 1 SNV LF unique to BLY |
| dHsaMDS306408698 |  | KLK12 | chr19 | 51535330 | - | G | A | Class 1 SNV LF unique to BLY |
| dHsaMDS417567458 |  | SLC22A7 | chr6 | 43266318 | + | T | C | Class 1 SNV LF unique to BLY |
| dHsaMDS859182481 |  | SIM1 | chr6 | 100838438 | - | G | C | Class 1 SNV LF unique to BLY |
| dHsaMDS743394744 |  | DENND2C | chr1 | 115168600 | - | A | T | Class 1 Var@CNA |
| dHsaMDS394931251 |  | DYRK4 | chr12 | 4708908 | + | T | C | Class 1 Var@CNA |
| dHsaMDS836737470 |  | GPR162 | chr12 | 6935955 | + | T | C | Class 1 Var@CNA |
| dHsaMDS262255667 |  | TEX14 | chr17 | 56659018 | - | C | T | Class 1 Var@CNA |
| dHsaMDS405751058 |  | TUBD1 | chr17 | 57963537 | - | A | G | Class 1 Var@CNA |
| dHsaMDS747944985 |  | BRIP1 | chr17 | 59760996 | - | A | G | Class 1 Var@CNA |
| dHsaMDS958765959 |  | BRIP1 | chr17 | 59763347 | - | A | G | Class 1 Var@CNA |
| dHsaMDS477509235 |  | ITGA6 | chr2 | 173352458 | + | C | G | Class 1 Var@CNA |
| dHsaMDS408384193 |  | ZMYND8 | chr20 | 45853037 | - | A | G | Class 1 Var@CNA |
| dHsaMDS491158027 |  | PREX1 | chr20 | 47262549 | - | C | T | Class 1 Var@CNA |
| dHsaMDS275927042 |  | PREX1 | chr20 | 47307618 | - | G | A | Class 1 Var@CNA |
| dHsaMDS520115562 |  | SALL4 | chr20 | 50408377 | - | G | C | Class 1 Var@CNA |
| dHsaMDS318180179 |  | PRICKLE2 | chr3 | 64142859 | - | C | T | Class 1 Var@CNA |
| dHsaMDS288350975 |  | SEMA5A | chr5 | 9044674 | - | G | A | Class 1 Var@CNA |
| dHsaMDS784435839 |  | TRIO | chr5 | 14389469 | + | C | T | Class 1 Var@CNA |
| dHsaMDS464803381 |  | CDH6 | chr5 | 31302288 | + | C | T | Class 1 Var@CNA |
| dHsaMDS915051733 |  | CDH6 | chr5 | 31317952 | + | T | C | Class 1 Var@CNA |
| dHsaMDS241745519 |  | AGXT2 | chr5 | 35037115 | - | C | T | Class 1 Var@CNA |
| dHsaMDS303090022 |  | SPEF2 | chr5 | 35670303 | + | G | A | Class 1 Var@CNA |
| dHsaMDS793987508 |  | ADAMTS19 | chr5 | 129040056 | + | A | T | Class 1 Var@CNA |
| dHsaMDS213731558 | COSM3996743 | MTOR | chr1 | 11181327 | - | C | T | Class 2 SNV COSMIC |
| dHsaMDS502101423 | COSM4142157 | MTOR | chr1 | 11301714 | - | A | G | Class 2 SNV COSMIC |
| dHsaMDS856946806 | COSM4142934 | SPEN | chr1 | 16256007 | + | T | C | Class 2 SNV COSMIC |
| dHsaMDS902497592 | COSM6943377 | SPEN | chr1 | 16258405 | + | C | T | Class 2 SNV COSMIC |
| dHsaMDS180025739 | COSM3751252 | MUTYH | chr1 | 45797505 | - | C | G | Class 2 SNV COSMIC |
| dHsaMDS335615569 | COSM5762903 | NTRK1 | chr1 | 156830779 | + | G | A | Class 2 SNV COSMIC |
| dHsaMDS748406820 | COSM425019 | ELF3 | chr1 | 201981774 | + | G | A | Class 2 SNV COSMIC |
| dHsaMDS220018471 | COSM5019897 | CYP2C8 | chr10 | 96827030 | - | C | T | Class 2 SNV COSMIC |
| dHsaMDS695996814 | COSM5019135 | LMO1 | chr11 | 8246181 | - | A | G | Class 2 SNV COSMIC |
| dHsaMDS258374192 | COSM6494056 | NUMA1 | chr11 | 71714981 | - | G | A | Class 2 SNV COSMIC |
| dHsaMDS501381225 | COSM6494061 | NUMA1 | chr11 | 71717106 | - | G | A | Class 2 SNV COSMIC |
| dHsaMDS572954748 | COSM5020622 | KMT2A | chr11 | 118368665 | + | A | G | Class 2 SNV COSMIC |
| dHsaMDS964052294 | COSM5019532 | SH2B3 | chr12 | 111872722 | + | T | C | Class 2 SNV COSMIC |
| dHsaMDS567651531 | COSM3765730 | AKT1 | chr14 | 105239894 | - | C | T | Class 2 SNV COSMIC |
| dHsaMDS155409710 | COSM4128580 | FES | chr15 | 91428290 | + | C | T | Class 2 SNV COSMIC |
| dHsaMDS358889039 | COSM5019529 | TSC2 | chr16 | 2114407 | + | C | T | Class 2 SNV COSMIC |
| dHsaMDS206898678 | COSM4128736 | MYH11 | chr16 | 15850204 | - | A | G | Class 2 SNV COSMIC |
| dHsaMDS763031726 | COSM5019920 | MYH11 | chr16 | 15870032 | - | A | G | Class 2 SNV COSMIC |
| dHsaMDS898536273 | COSM4985454 | PALB2 | chr16 | 23646191 | - | T | C | Class 2 SNV COSMIC |
| dHsaMDS789330123 | COSM4986139 | ERBB2 | chr17 | 37855834 | + | C | A | Class 2 SNV COSMIC |
| dHsaMDS399835000 | COSM4130449 | RNF43 | chr17 | 56435885 | - | G | T | Class 2 SNV COSMIC |
| dHsaMDS363104339 | COSM6494512 | SRSF2 | chr17 | 74733099 | - | G | A | Class 2 SNV COSMIC |
| dHsaMDS831165069 | COSM437734 | RNF213 | chr17 | 78262161 | + | T | C | Class 2 SNV COSMIC |
| dHsaMDS294079812 | COSM5019332 | RNF213 | chr17 | 78346870 | + | C | A | Class 2 SNV COSMIC |
| dHsaMDS939125469 | COSM4140650 | MAP2K2 | chr19 | 4099272 | - | G | A | Class 2 SNV COSMIC |
| dHsaMDS183129089 | COSM3756290 | SMARCA4 | chr19 | 11101993 | + | G | A | Class 2 SNV COSMIC |
| dHsaMDS596216468 | COSM3756833 | CIC | chr19 | 42799049 | + | C | T | Class 2 SNV COSMIC |
| dHsaMDS424901267 | COSM5020025 | LRP1B | chr2 | 141267573 | - | G | A | Class 2 SNV COSMIC |
| dHsaMDS298889775 | COSM3117461 | LRP1B | chr2 | 141625371 | - | C | A | Class 2 SNV COSMIC |
| dHsaMDS584018974 | COSM6494786 | CUL3 | chr2 | 225346646 | - | T | C | Class 2 SNV COSMIC |
| dHsaMDS877070397 | COSM3760366 | FAT4 | chr4 | 126237971 | + | C | T | Class 2 SNV COSMIC |
| dHsaMDS905725928 | COSM3760371 | FAT4 | chr4 | 126239986 | + | C | T | Class 2 SNV COSMIC |
| dHsaMDS368635450 | COSM5008351 | FAT4 | chr4 | 126240510 | + | T | C | Class 2 SNV COSMIC |
| dHsaMDS517442425 | COSM3760373 | FAT4 | chr4 | 126241335 | + | C | G | Class 2 SNV COSMIC |
| dHsaMDS416091618 | COSM5008353 | FAT4 | chr4 | 126241871 | + | C | T | Class 2 SNV COSMIC |
| dHsaMDS849896621 | COSM4415581 | FAT4 | chr4 | 126372742 | + | G | A | Class 2 SNV COSMIC |
| dHsaMDS264678856 | COSM5019428 | LIFR | chr5 | 38486065 | - | C | T | Class 2 SNV COSMIC |
| dHsaMDS573545407 | COSM3761168 | FLT4 | chr5 | 180030313 | - | C | A | Class 2 SNV COSMIC |
| dHsaMDS651616231 | COSM5480661 | CDKN1A | chr6 | 36645696 | + | A | G | Class 2 SNV COSMIC |
| dHsaMDS880228172 | COSM3762618 | CARD11 | chr7 | 2946461 | - | T | C | Class 2 SNV COSMIC |
| dHsaMDS374876146 | COSM4416211 | AKAP9 | chr7 | 91691601 | + | C | T | Class 2 SNV COSMIC |
| dHsaMDS406576371 | COSM6495117 | CUX1 | chr7 | 101747623 | + | G | A | Class 2 SNV COSMIC |
| dHsaMDS721015466 | COSM1568187 | CUX1 | chr7 | 101837149 | + | G | A | Class 2 SNV COSMIC |
| dHsaMDS568192952 | COSM4161680 | CUX1 | chr7 | 101844851 | + | A | G | Class 2 SNV COSMIC |
| dHsaMDS977047741 | COSM1755000 | CUX1 | chr7 | 101917521 | + | G | A | Class 2 SNV COSMIC |
| dHsaMDS703675486 | COSM1755070 | SMO | chr7 | 128845223 | + | C | T | Class 2 SNV COSMIC |
| dHsaMDS555439772 | COSM4161857 | BRAF | chr7 | 140449150 | - | T | C | Class 2 SNV COSMIC |
| dHsaMDS674373106 | COSM6495174 | EZH2 | chr7 | 148515192 | - | A | G | Class 2 SNV COSMIC |
| dHsaMDS684308118 | COSM150522 | EXT1 | chr8 | 118847782 | - | G | A | Class 2 SNV COSMIC |
| dHsaMDS483277830 | COSM6938976 | NOTCH1 | chr9 | 139412205 | - | G | A | Class 2 SNV COSMIC |
| dHsaMDV2010069 | COSM583 | NRAS | chr1 | 115256529 | - | A | T | Investigational |
| dHsaMDS794087263 |  | NRAS | chr1 | 115258669 | - | A | T | Investigational |
| dHsaMDV2510530 | COSM562 | NRAS | chr1 | 115258748 | - | G | T | Investigational |
| dHsaMDS485465667 | COSM1159820 | RET | chr10 | 43613908 | + | A | T | Investigational |
| dHsaMDS344791716 | COSM29832 | FGFR2 | chr10 | 123298199 | - | C | T | Investigational |
| dHsaMDS706173836 |  | KRAS | chr12 | 25380233 | - | C | G | Investigational |
| dHsaMDV2510586 | COSM522 | KRAS | chr12 | 25398284 | - | G | C | Investigational |
| dHsaMDS283890588 | COSM13010 | PTPN11 | chr12 | 112888162 | + | G | C | Investigational |
| dHsaMDS168526746 | COSM935848 | RAB35 | chr12 | 120536641 | - | C | T | Investigational |
| dHsaMDS2514068 | COSM18606 | FLT3 | chr13 | 28608087 | - | C | T | Investigational |
| dHsaMDS302129293 | COSM1678546 | MAP2K1 | chr15 | 66727483 | + | G | A | Investigational |
| dHsaMDS400568388 | COSM235614 | MAP2K1 | chr15 | 66729162 | + | C | T | Investigational |
| dHsaMDV2510552 | COSM10663 | TP53 | chr17 | 7577022 | - | C | T | Investigational |
| dHsaMDS535511007 | COSM10722 | TP53 | chr17 | 7577085 | - | C | T | Investigational |
| dHsaMDS2516048 | COSM10769 | TP53 | chr17 | 7577118 | - | C | A | Investigational |
| dHsaMDS2514160 | COSM10735 | TP53 | chr17 | 7578211 | - | C | T | Investigational |
| dHsaMDS325663947 |  | TP53 | chr17 | 7578448 | - | G | T | Investigational |
| dHsaMDS579102032 | COSM18654 | TP53 | chr17 | 7578469 | - | C | - (del) | Investigational |
| dHsaMDS2511476 | COSM51374 | JAK3 | chr19 | 17949108 | - | C | T | Investigational |
| dHsaMDV2510548 | COSM5664 | CTNNB1 | chr3 | 41266124 | + | A | G | Investigational |
| dHsaMDV2010075 | COSM763 | PIK3CA | chr3 | 178936091 | + | G | A | Investigational |
| dHsaMDS533259606 |  | SOX2 | chr3 | 181430978 | + | A | C | Investigational |
| dHsaMDS823442540 |  | FGFR3 | chr4 | 1803385 | + | G | C | Investigational |
| dHsaMDV2516760 | COSM715 | FGFR3 | chr4 | 1803568 | + | C | G | Investigational |
| dHsaMDS2514232 | COSM28026 | KIT | chr4 | 55593464 | + | A | C | Investigational |
| dHsaMDV2010023 | COSM1314 | KIT | chr4 | 55599321 | + | A | T | Investigational |
| dHsaMDS610702609 |  | KIT | chr4 | 55599360 | + | T | A | Investigational |
| dHsaMDS127477390 | COSM149673 | KDR | chr4 | 55972974 | - | T | A | Investigational |
| dHsaMDS164103691 | COSM2155617 | PIK3R1 | chr5 | 67589147 | + | A | G | Investigational |
| dHsaMDS534456141 |  | EGFR | chr7 | 55242482 | + | C | T | Investigational |
| dHsaMDV2010019 | COSM6240 | EGFR | chr7 | 55249071 | + | C | T | Investigational |
| dHsaMDS266005801 | COSM53104 | EGFR | chr7 | 55249092 | + | G | A | Investigational |
| dHsaMDS362259477 |  | EGFR | chr7 | 55249235 | + | C | A | Investigational |
| dHsaMDV2010021 | COSM6224 | EGFR | chr7 | 55259515 | + | T | G | Investigational |
| dHsaMDS2513878 | COSM707 | MET | chr7 | 116411990 | + | C | T | Investigational |
| dHsaMDS568624045 |  | MET | chr7 | 116412044 | + | G | T | Investigational |
| dHsaMDV2010027 | COSM476 | BRAF | chr7 | 140453136 | - | A | T | Investigational |
| dHsaMDV2010061 | COSM12600 | JAK2 | chr9 | 5073770 | + | G | T | Investigational |
| dHsaMDS487367531 |  | CD274 | chr9 | 5467955 | + | G | A | Investigational |
| dHsaMDS2512004 | COSM12771 | NOTCH1 | chr9 | 139399344 | - | A | G | Investigational |
| dHsaMDS352165583 |  | NOTCH1 | chr9 | 139399897 | - | T | G | Investigational |
| dHsaMDS949669741 | COSM3997965 | RET | chr10 | 43620335 | + | C | T | Investigational* |
| dHsaMDS404236397 | COSM6984196 | TSC2 | chr16 | 2115506 | + | C | T | Investigational* |
| dHsaMDS547535260 | COSM96507 | CREBBP | chr16 | 3795363 | - | G | A | Investigational* |
| dHsaMDS513410713 | COSM27386 | NF1 | chr17 | 29687504 | + | G | A | Investigational* |
| dHsaMDS162559440 | COSM5019249 | SMAD2 | chr18 | 45371695 | - | A | G | Investigational* |
| dHsaMDS758039626 | COSM4416085 | RAF1 | chr3 | 12645007 | - | C | T | Investigational* |
| dHsaMDS909542383 | COSM6952009 | BAP1 | chr3 | 52440827 | - | C | A | Investigational* |
| dHsaMDS719612314 | COSM5019327 | SBDS | chr7 | 66453476 | - | A | G | Investigational* |
| dHsaMDS485514048 | COSM14252 | CDKN2A | chr9 | 21968159 | - | G | A | Investigational* |
| dHsaMDS993135503 | COSM3759436 | ZRSR2 | chrX | 15838366 | + | C | T | Investigational* |
| dHsaMDS482125089 | COSM6925171 | KDM6A | chrX | 44733136 | + | A | T | Investigational* |
| dHsaMDS2513748 | COSM27286 | MPL | chr1 | 43814979 | + | G | A | Neg |
| dHsaMDS2512816 | COSM503 | HRAS | chr11 | 533873 | - | C | G | Neg |
| dHsaMDS769273707 | COSM1644274 | CCND1 | chr11 | 69456211 | + | T | G | Neg |
| dHsaMDS439915394 | COSM180943 | ETV6 | chr12 | 12037474 | + | C | T | Neg |
| dHsaMDS808994749 | COSM4384670 | ETV6 | chr12 | 12037475 | + | G | A | Neg |
| dHsaMDS360123786 | COSM6023570 | SMAD3 | chr15 | 67473692 | + | G | A | Neg |
| dHsaMDS339089491 | COSM6023586 | SMAD3 | chr15 | 67482818 | + | G | T | Neg |
| dHsaMDS2511414 | COSM48358 | ERBB2 | chr17 | 37868208 | + | C | T | Neg |
| dHsaMDS525644749 | COSM4075497 | JAK3 | chr19 | 17948862 | - | A | C | Neg |
| dHsaMDS567694131 | COSM51210 | PPP2R1A | chr19 | 52715982 | + | C | G | Neg |
| dHsaMDS843931504 | COSM6188567 | EPAS1 | chr2 | 46607399 | + | G | A | Neg |
| dHsaMDS177787972 | COSM190062 | MSH6 | chr2 | 48026204 | + | G | A | Neg |
| dHsaMDV2510562 | COSM27887 | GNAS | chr20 | 57484420 | + | C | T | Neg |
| dHsaMDV2516796 | COSM27895 | GNAS | chr20 | 57484421 | + | G | A | Neg |
| dHsaMDS315076176 | COSM17894 | VHL | chr3 | 10183864 | + | C | G | Neg |
| dHsaMDS719308991 | COSM6196869 | VHL | chr3 | 10188201 | + | A | G | Neg |
| dHsaMDS431429677 | COSM6503490 | VHL | chr3 | 10188202 | + | C | G | Neg |
| dHsaMDS2511768 | COSM14312 | VHL | chr3 | 10188210 | + | T | C | Neg |
| dHsaMDS605653313 | COSM188063 | CTNNB1 | chr3 | 41274911 | + | T | G | Neg |
| dHsaMDV2510558 | COSM746 | PIK3CA | chr3 | 178916876 | + | G | A | Neg |
| dHsaMDS328946589 | COSM27493 | PIK3CA | chr3 | 178916890 | + | C | T | Neg |
| dHsaMDS2510604 | COSM771 | PIK3CA | chr3 | 178952018 | + | A | G | Neg |
| dHsaMDV2510574 | COSM12597 | PIK3CA | chr3 | 178952090 | + | G | C | Neg |
| dHsaMDS541553933 | COSM42038 | TET2 | chr4 | 106164069 | + | T | G | Neg |
| dHsaMDS518045307 | COSM1451536 | EGFR | chr7 | 55211079 | + | A | G | Neg |
| dHsaMDS2511196 | COSM21683 | EGFR | chr7 | 55211080 | + | G | A | Neg |
| dHsaMDS741433983 | COSM3412174 | *EGFR* | chr7 | 55220295 | + | A | T | Neg |
| dHsaMDS682025590 | COSM21684 | EGFR | chr7 | 55221743 | + | A | C | Neg |
| dHsaMDS2515220 | COSM21687 | EGFR | chr7 | 55221822 | + | C | T | Neg |
| dHsaMDS784745459 | COSM236671 | EGFR | chr7 | 55228007 | + | A | C | Neg |
| dHsaMDS2513904 | COSM13424 | EGFR | chr7 | 55259439 | + | T | G | Neg |
| dHsaMDV2010043 | COSM6213 | EGFR | chr7 | 55259524 | + | T | A | Neg |
| dHsaMDS502778724 | COSM13197 | EGFR | chr7 | 55259532 | + | G | A | Neg |
| dHsaMDS931471131 | COSM707337 | EGFR | chr7 | 55259535 | + | G | A | Neg |
| dHsaMDS258234785 | COSM694 | MET | chr7 | 116423475 | + | G | A | Neg |
| dHsaMDS750221389 | COSM216037 | SMO | chr7 | 128846398 | + | C | T | Neg |
| dHsaMDS2514940 | COSM12608 | ABL1 | chr9 | 133738330 | + | A | G | Neg |
| dHsaMDS995710939 | COSM131574 | ABL1 | chr9 | 133750328 | + | T | A | Neg |
| dHsaMDS991864975 | COSM12604 | ABL1 | chr9 | 133750356 | + | A | G | Neg |
