# Supplementary material for: A verified genomic reference sample for assessing performance of cancer panels detecting small variants of low allele frequency
Source: Genome Biol. 2021 Apr 16;22:111. doi: 10.1186/s13059-021-02316-z (PMC8051128; doi:10.1186/s13059-021-02316-z)
Supplement: Supplementary file 2 — Additional file 2:. Fig. S1. Unique Fragments, total Deduplicated Depth, and Coverage of Sample A by Exome enrichment kit replicate. WES1–3 are Illumina sequencing libraries. Fig. S2. Data related to Thermo Fisher’s WES panel related to its a) sensitivity by cell line at VAF greater than 0.1 and 0.2, b) total number of WES4 variants called relative to total positives by cell line at VAF greater than 0.1 and 0.2 and c) precision (or positive predictive value) of the WES4 panel on individual cell line variants. Fig. S3. TLY VAF profile at top where common variants from dbSNP are in red (het) or green (hom). Variants not found in dbSNP are in orange. Since TLY has an asymmetric VAF profile indicated in by the somatic variants in orange, the reasonable explanation (combined with cytology) is that TLY is inherently tetraploid with some noticeable large sCNA (e.g., chr 4, 7, 8, 17 and 20 are most noteworthy) and that the somatic variants are all near 0.25 as somatic changes are randomly scattered among the two chromosome pairs from the cancer cell, rarely affecting both. In essence, cell line TLY is a fusion of a normal and cancerous cell. Fig. S4. VAF histogram of Sample A for Class 2 variants only (COSMIC genes but not in CTR). Fig. S5. a-j) Histograms of VAF using all positives (Class 1 and Class 2) contributed by each of the 10 cell lines. Class 1 positives are > 99% of all positives. k-t) Histograms of VAF using only Class 2 positives contributed by each of the 10 cell lines. Fig. S6. Concordance in VAF of Pooled Sample A between bait captures, in silico results, merged-BAM library results and the averaging of individual cell line results. Results between same kits are slightly more highly correlated. Cell line average results are concordant except for a small number of variants, many related to CNA influences from individual cell lines. Fig. S7. Concordance of putative positives (variants) from CTR using WES1–3. The area of each figure represents the magnitude of th [file 13059_2021_2316_MOESM2_ESM.docx]

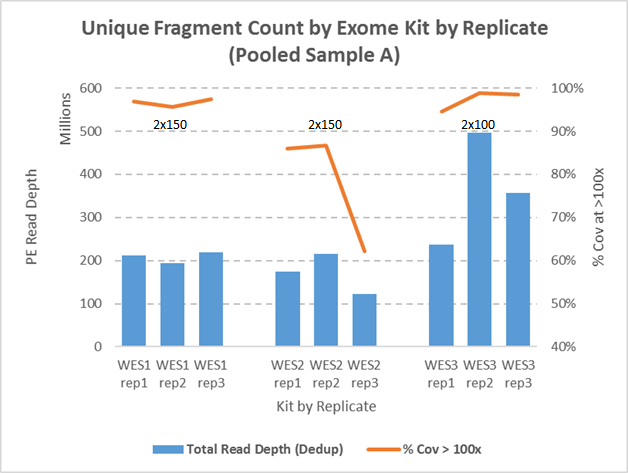


**Fig. S1: Unique Fragments, total Deduplicated Depth, and Coverage of Sample A by Exome enrichment kit replicate. WES1-3 are Illumina sequencing libraries**


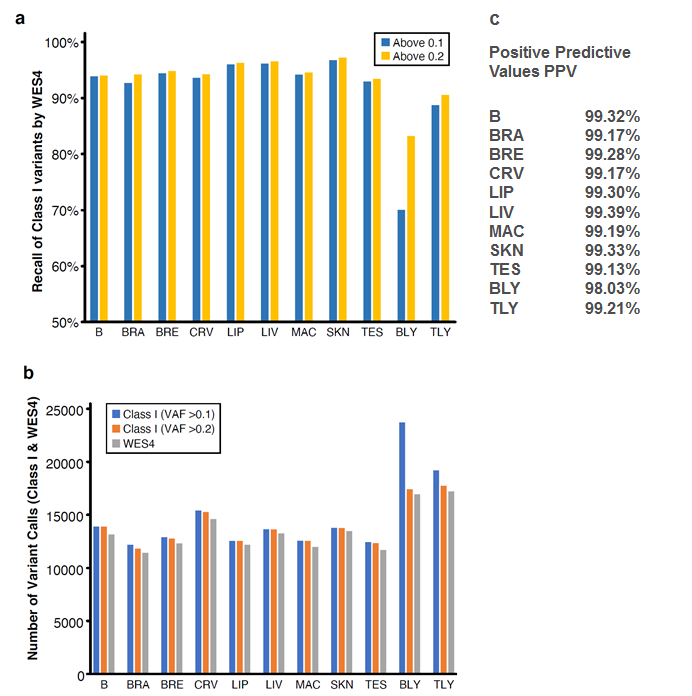


**Fig. S2: Data related to Thermo Fisher’s WES panel related to its a) sensitivity by cell line at VAF greater than 0.1 and 0.2, b) total number of WES4 variants called relative to total positives by cell line at VAF greater than 0.1 and 0.2 and c) precision (or positive predictive value) of the WES4 panel on individual cell line variants**


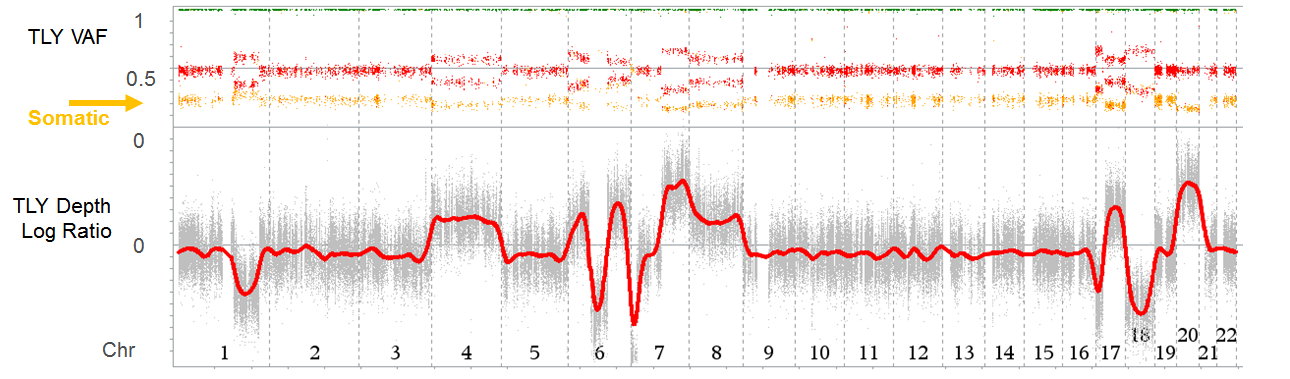
 **Fig. S3: TLY VAF profile at top where common variants from dbSNP are in red (het) or green (hom). Variants not found in dbSNP are in orange. Since TLY has an asymmetric VAF profile indicated in by the somatic variants in orange, the reasonable explanation (combined with cytology) is that TLY is inherently tetraploid with some noticeable large sCNA (e.g., chr 4, 7, 8, 17 and 20 are most noteworthy) and that the vast majority of somatic variants are near 0.25 as somatic changes are randomly scattered among the two chromosome pairs from the cancer cell, rarely affecting both. In essence, cell line TLY is a fusion of a normal and cancerous cell.**


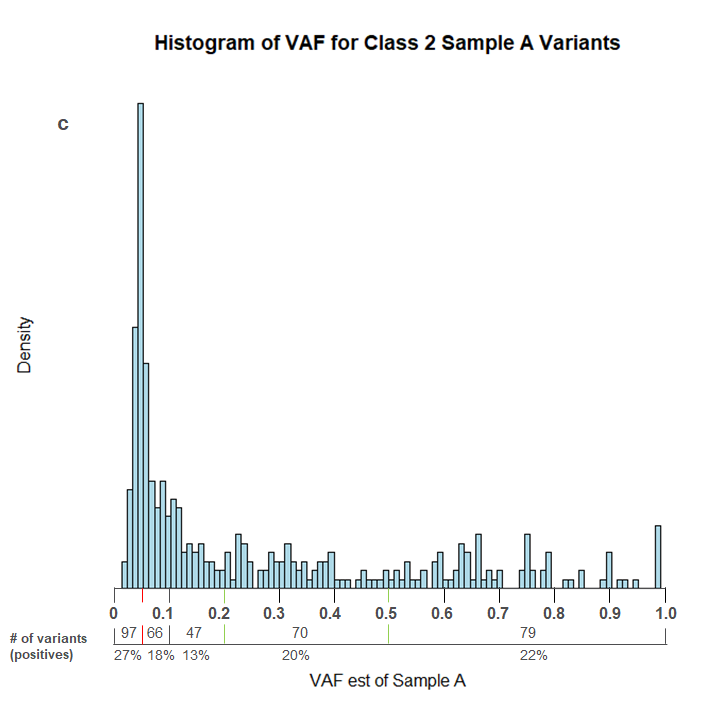


**Fig. S4: VAF histogram of Sample A for Class 2 variants only (COSMIC genes but not in CTR).**

**
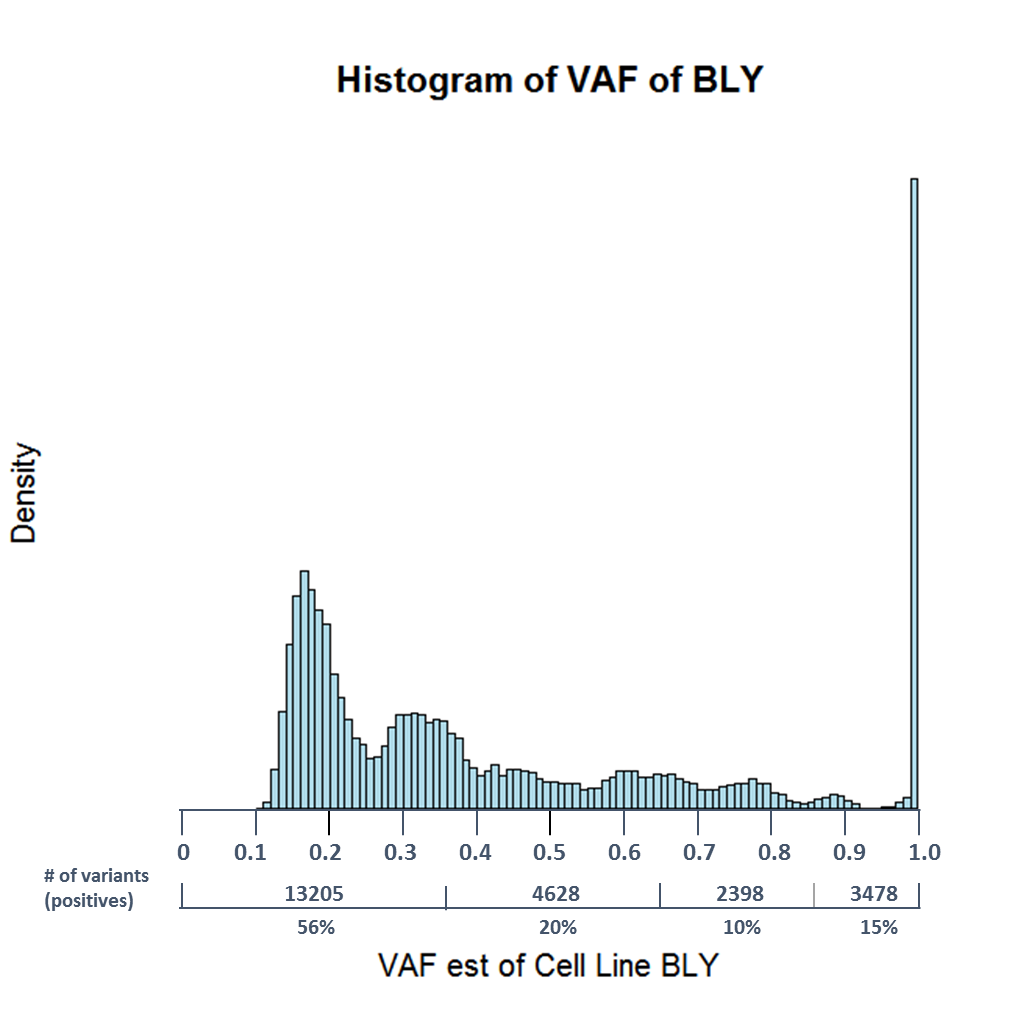
**

**
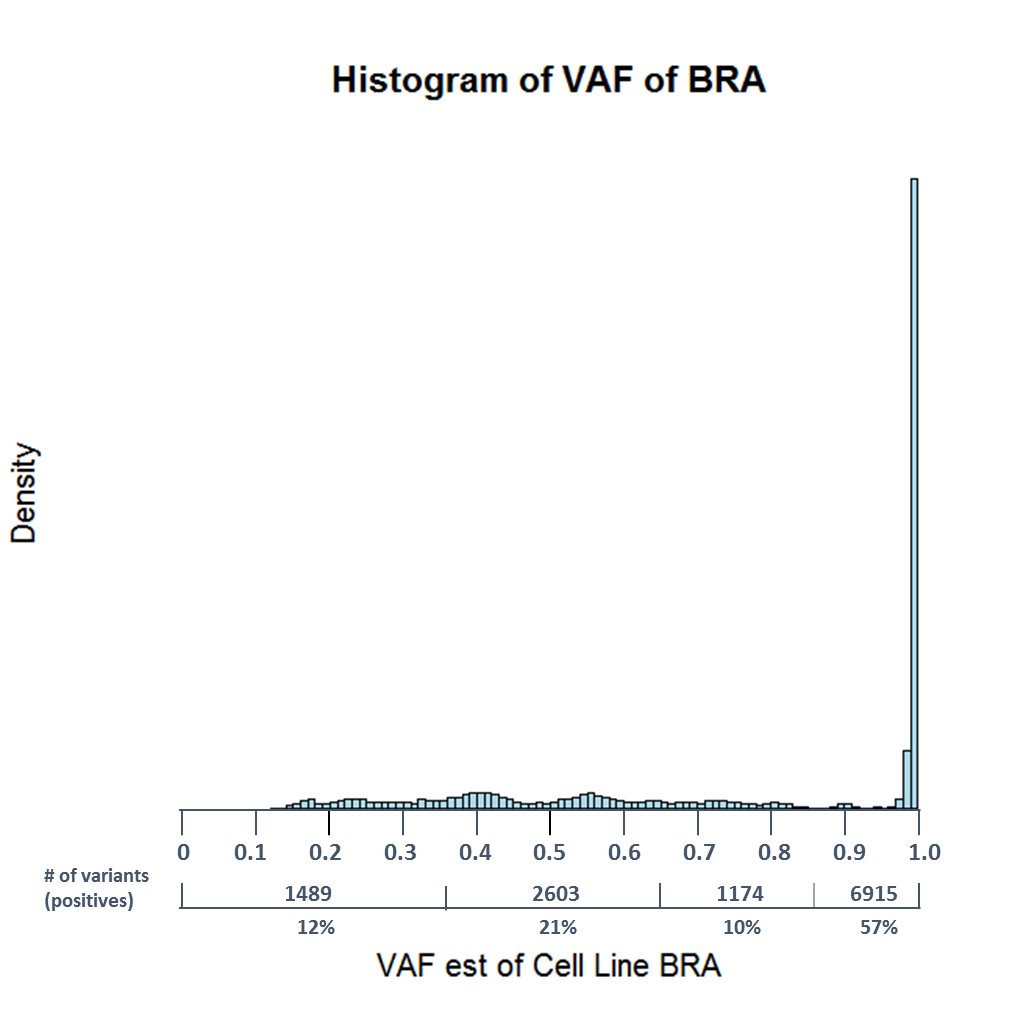

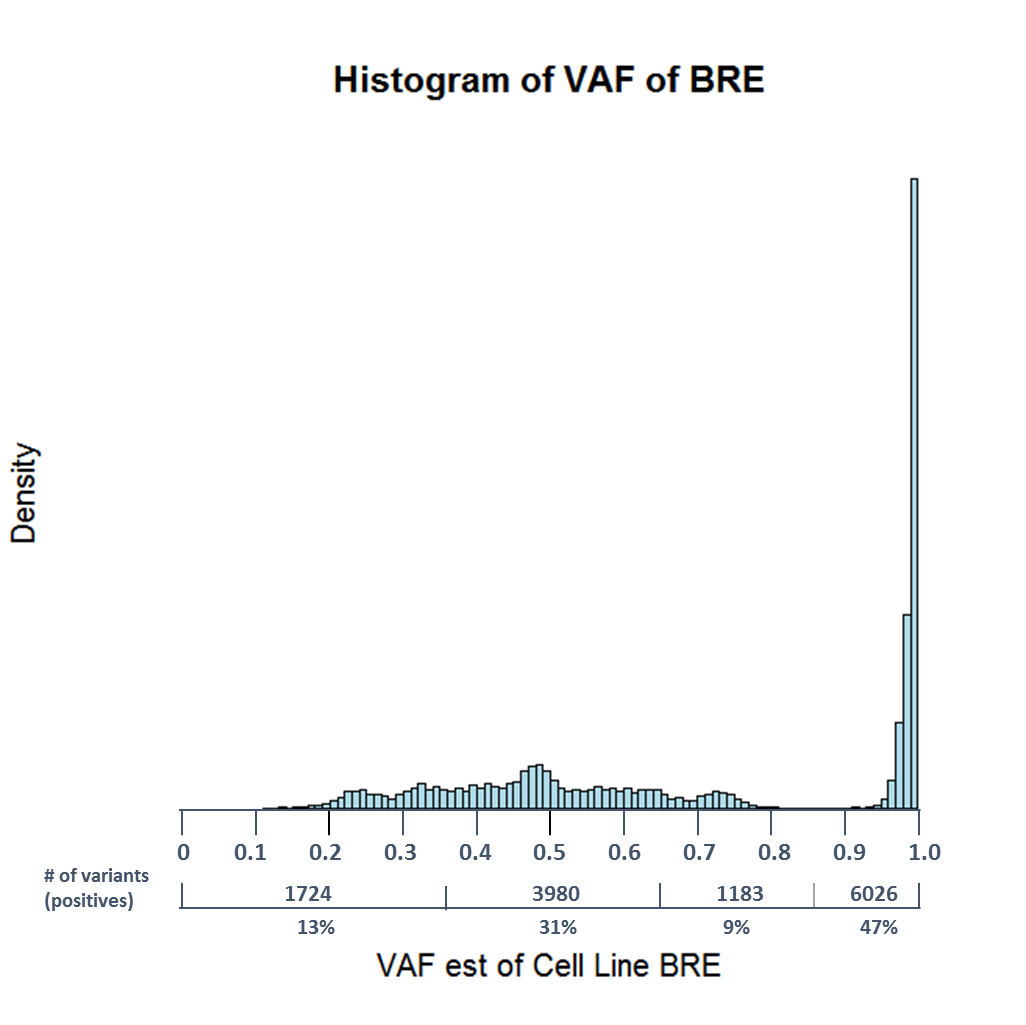

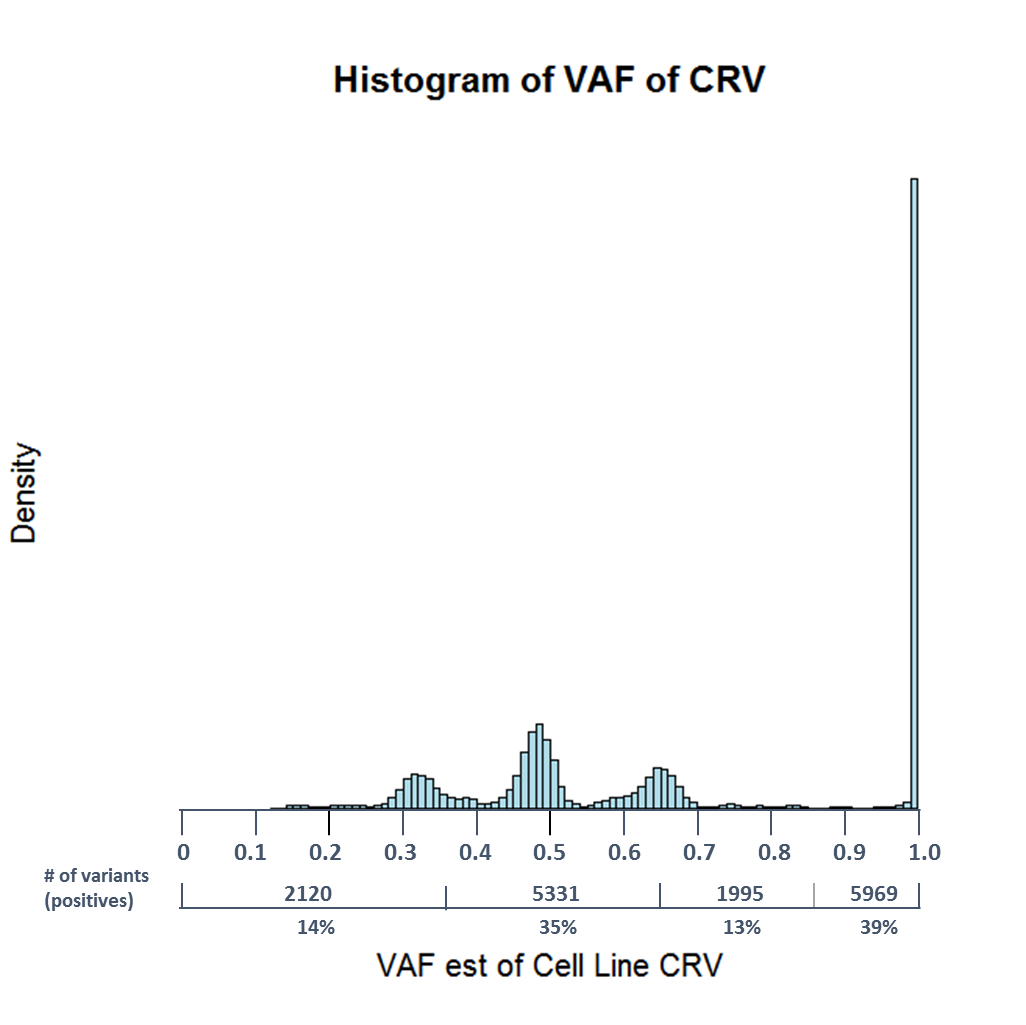
**

**a b**

**c d**


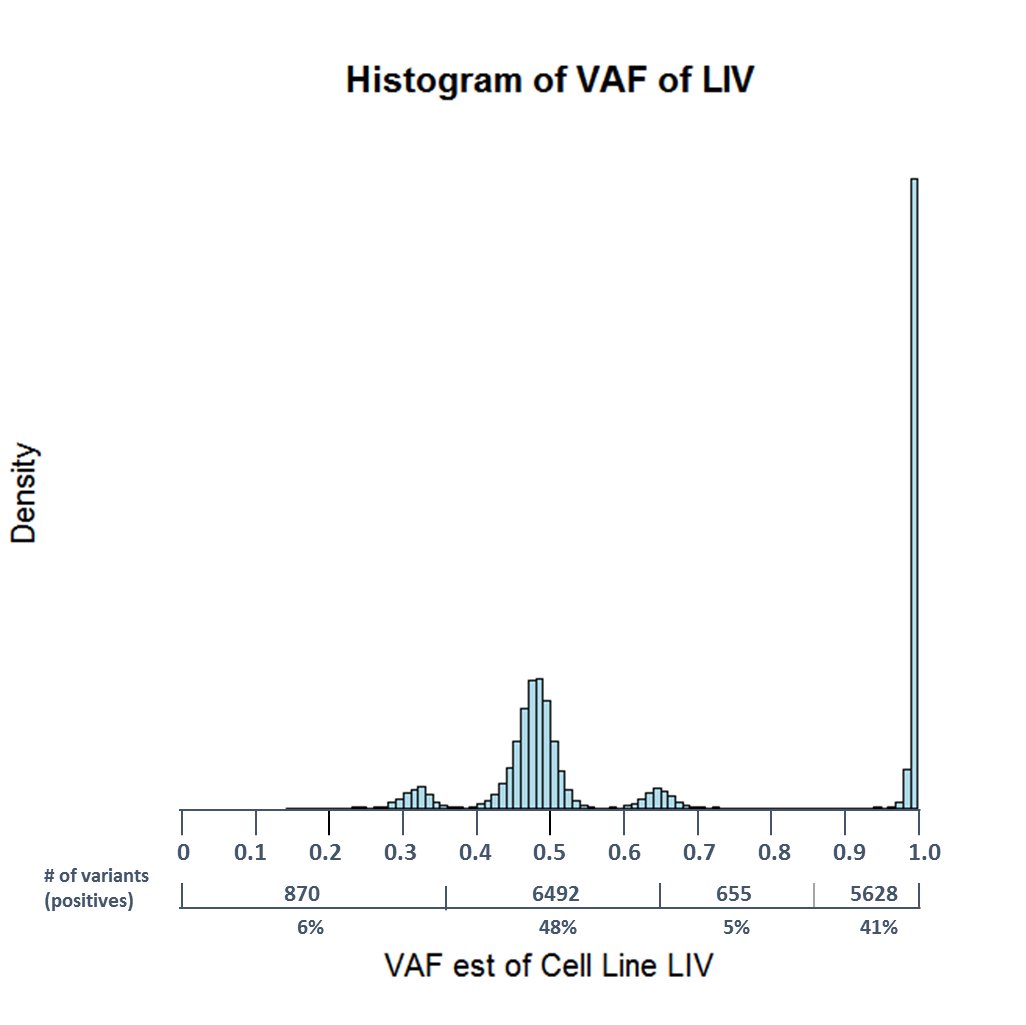

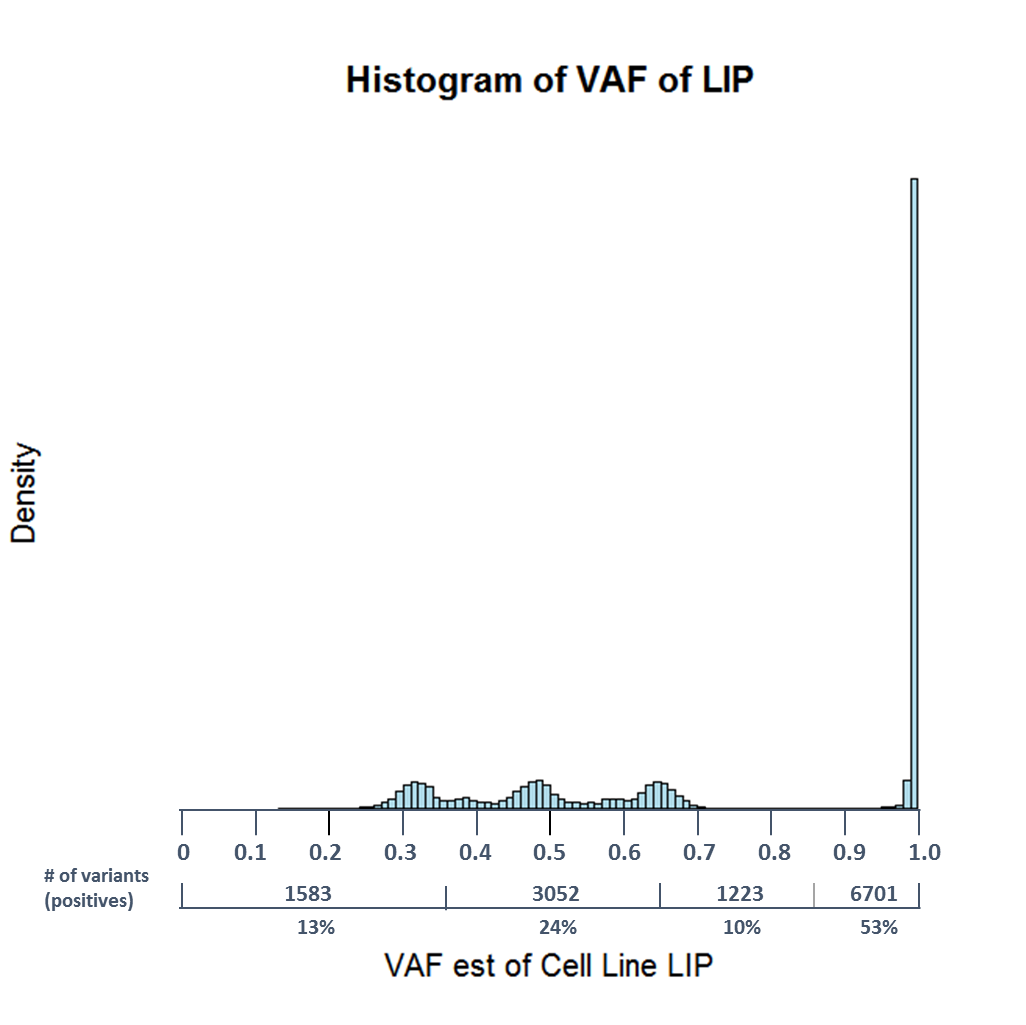


**e f**


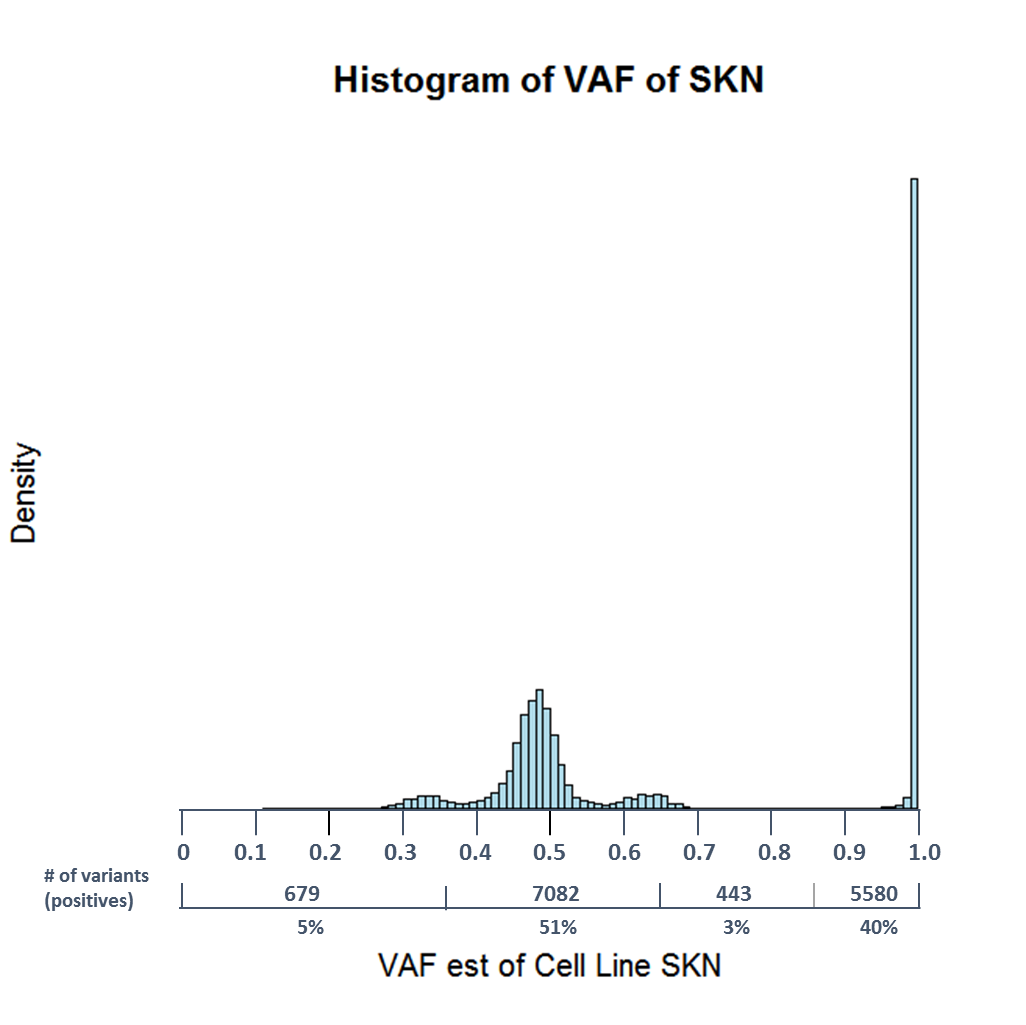

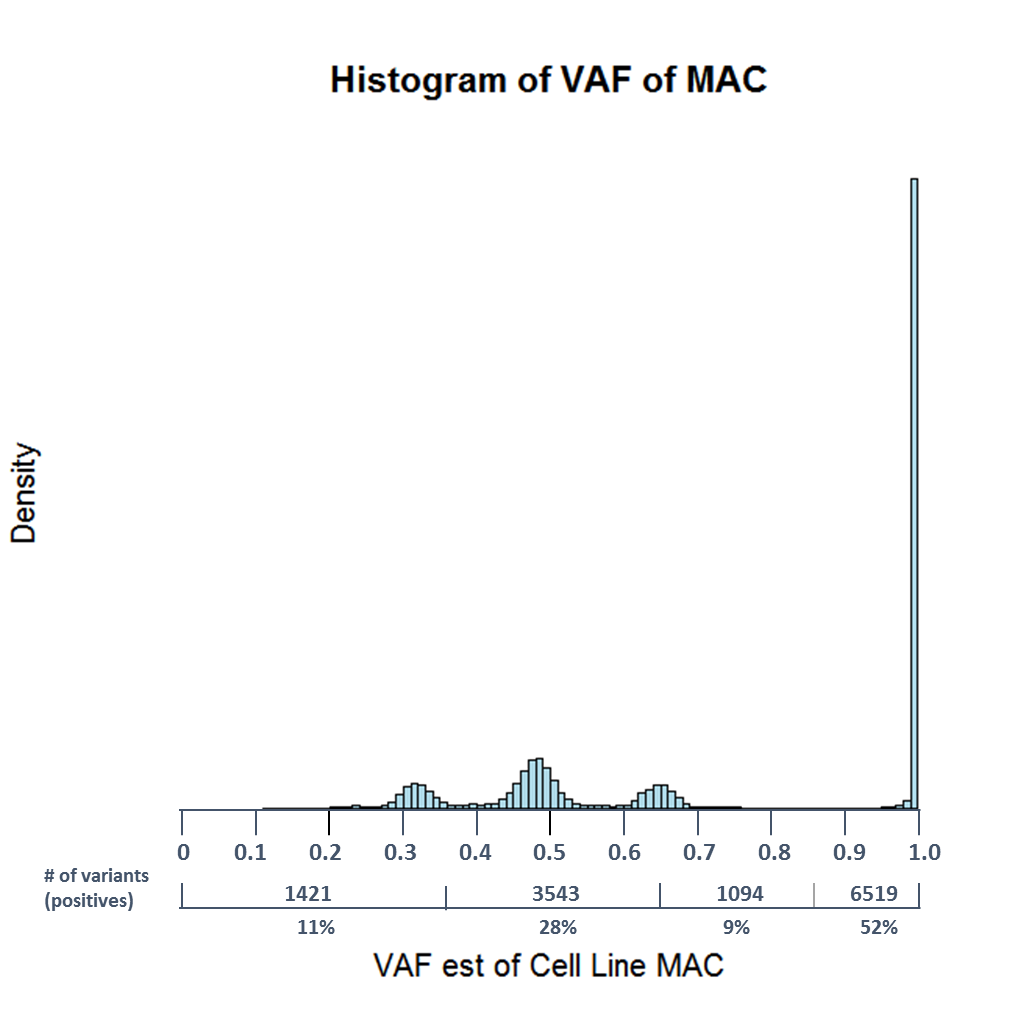


**g h**


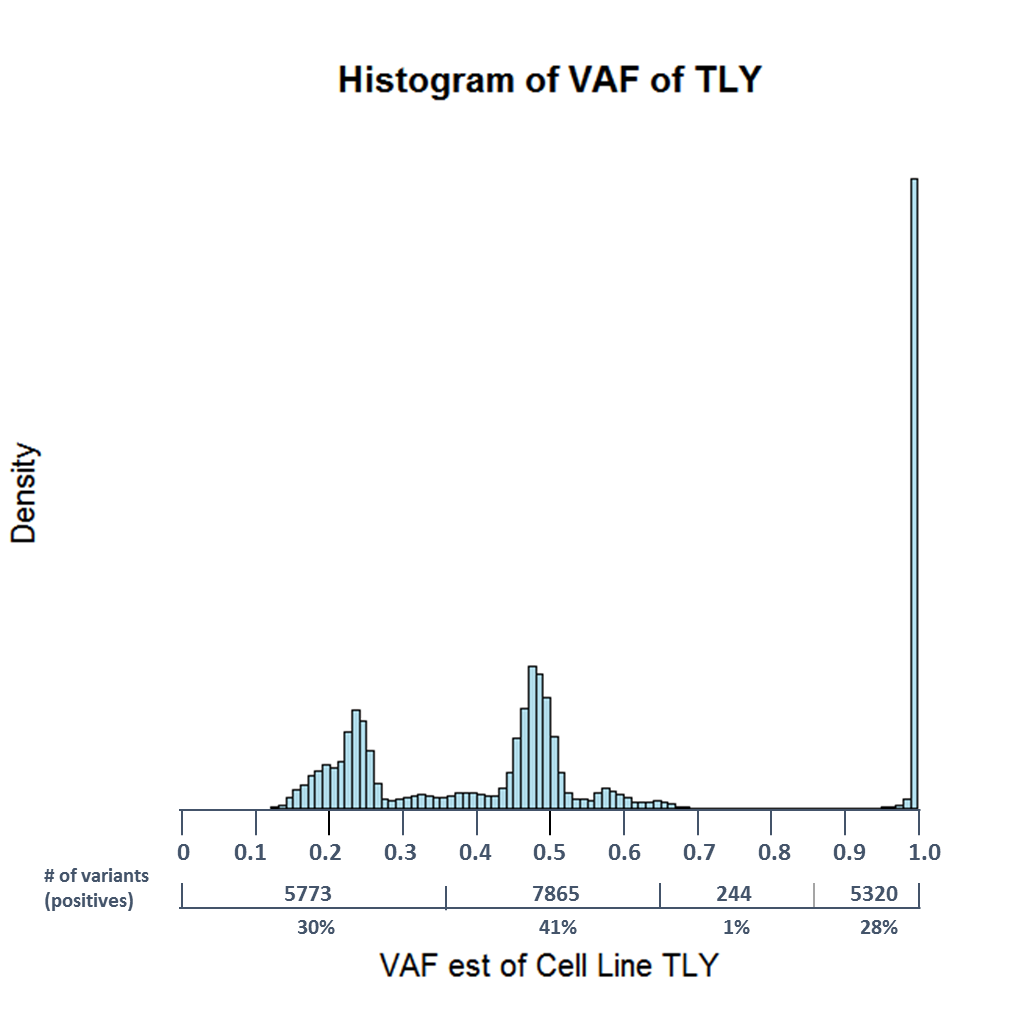

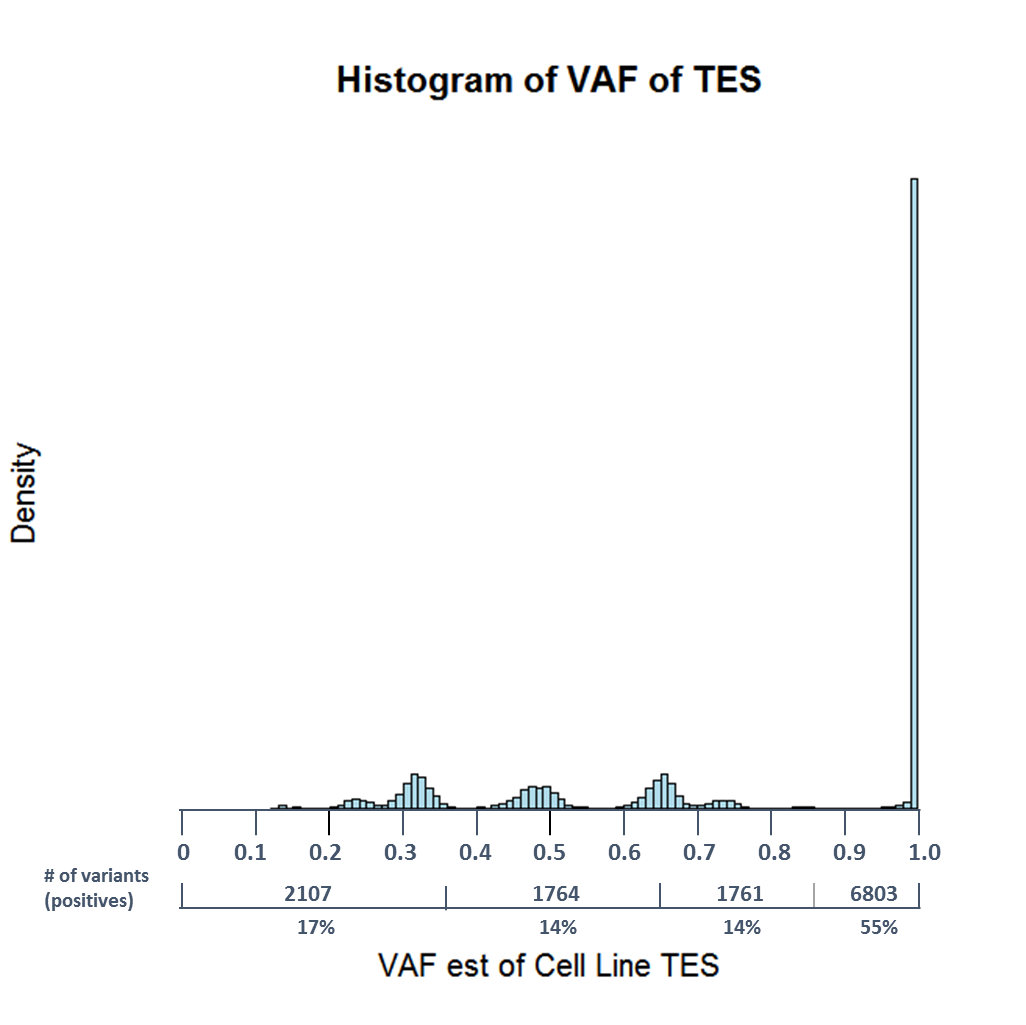


**i j**


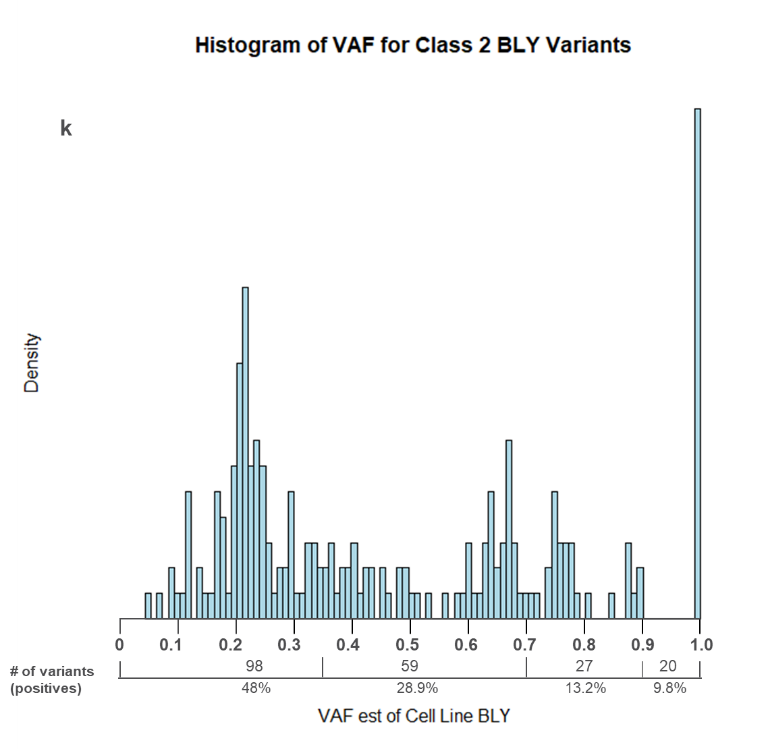

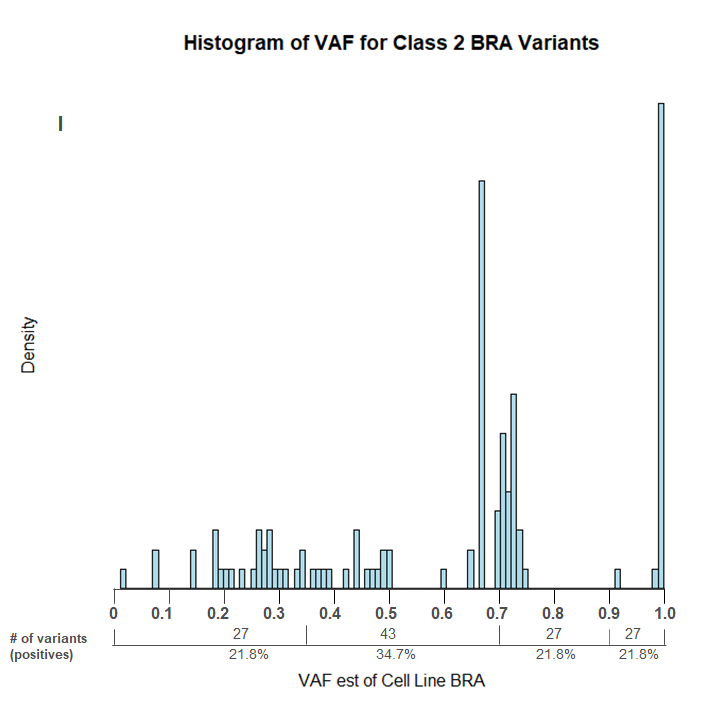


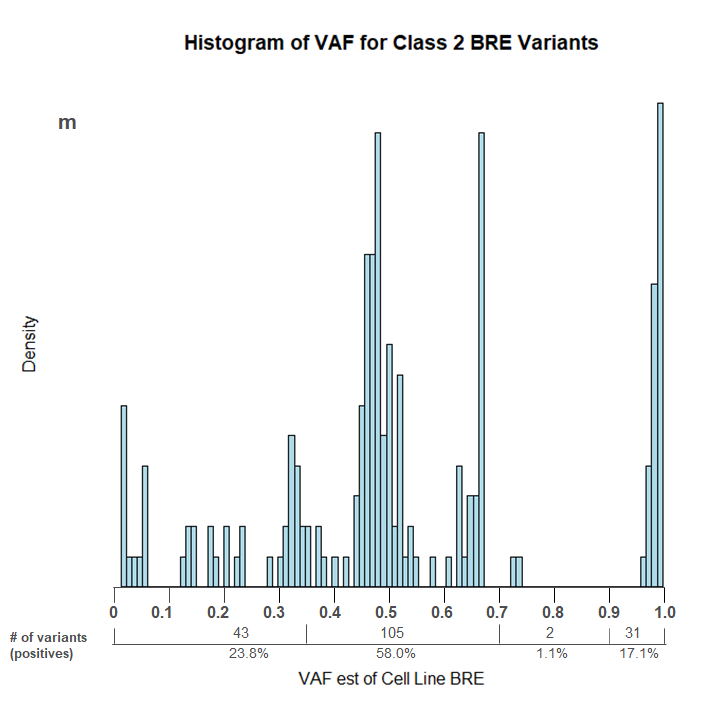

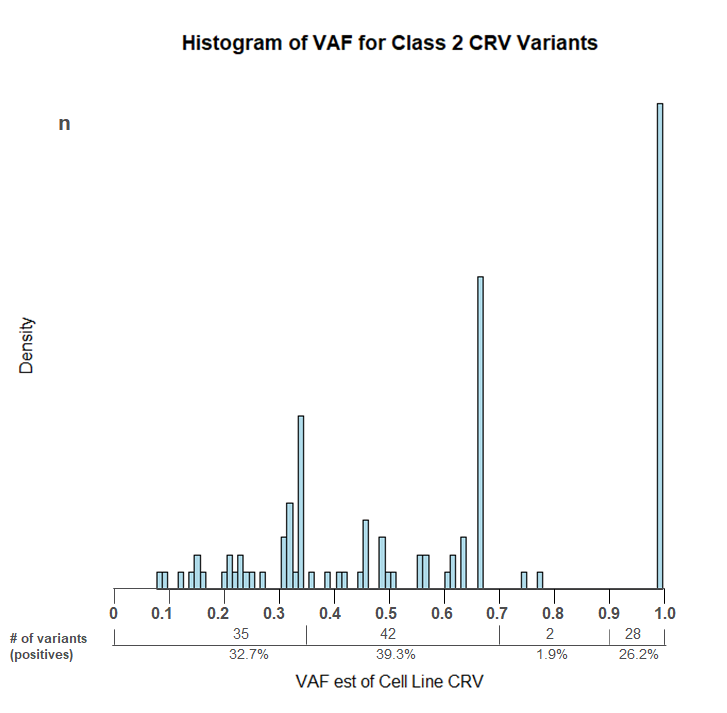


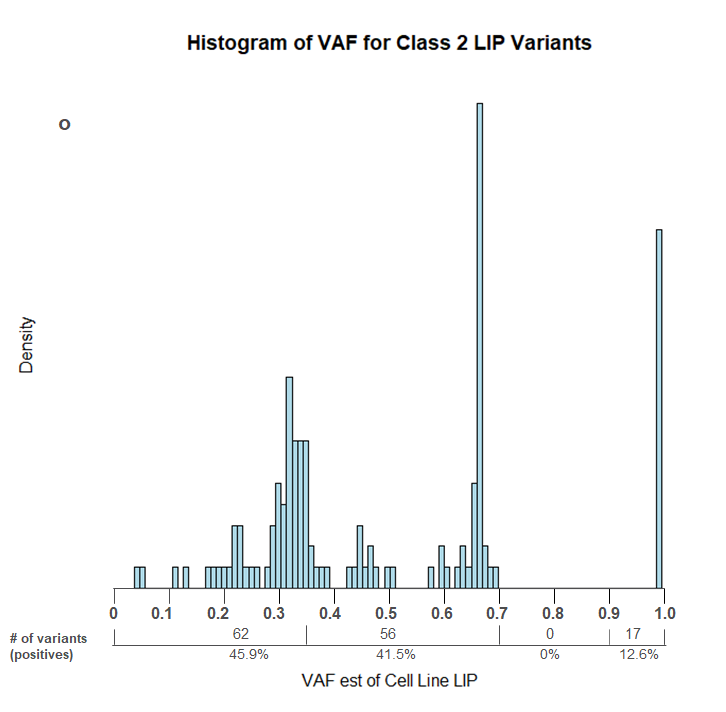

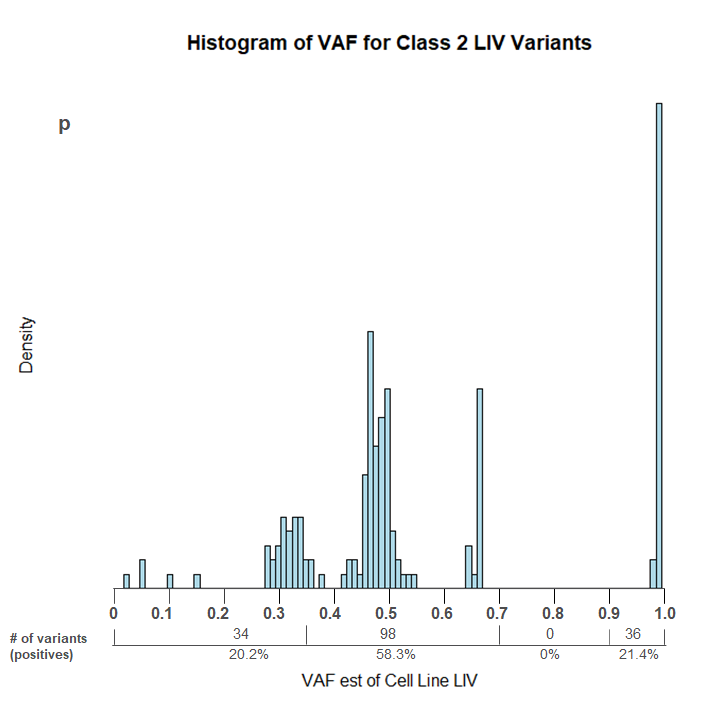


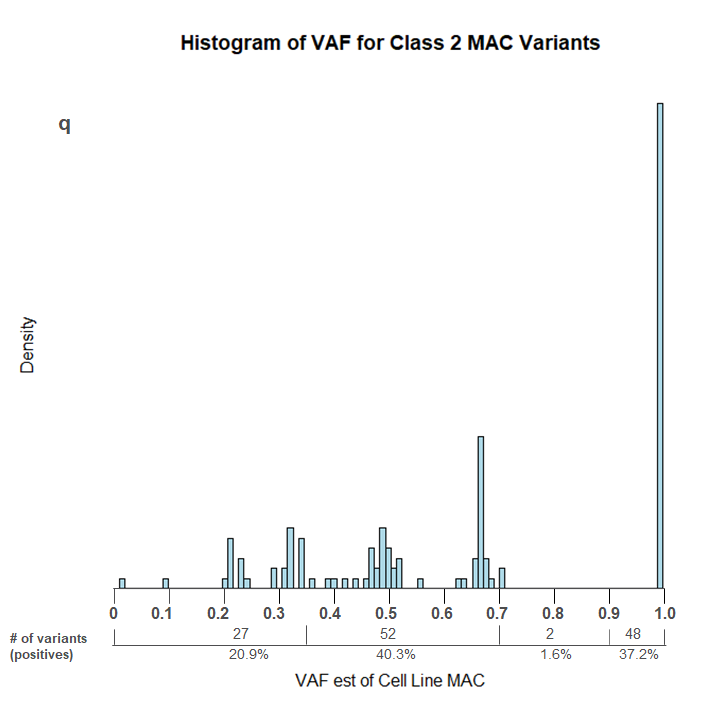

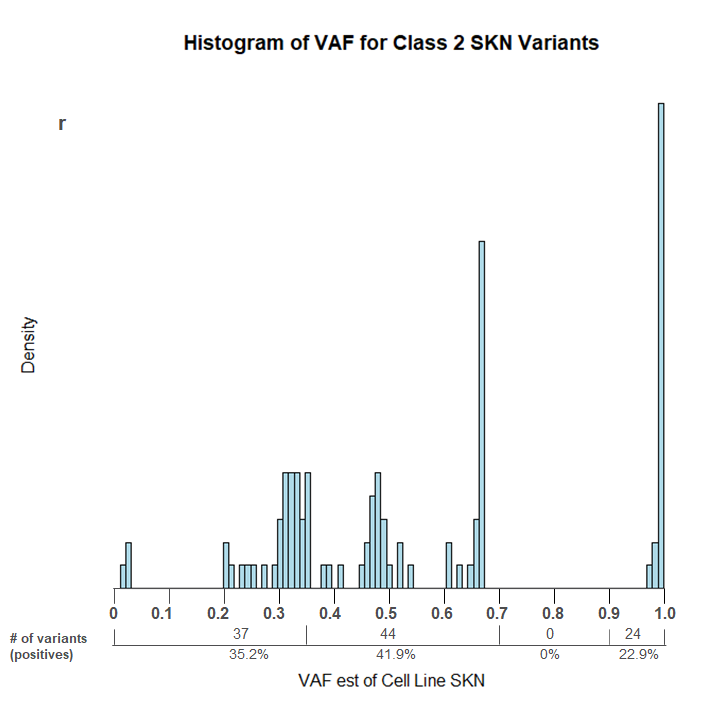


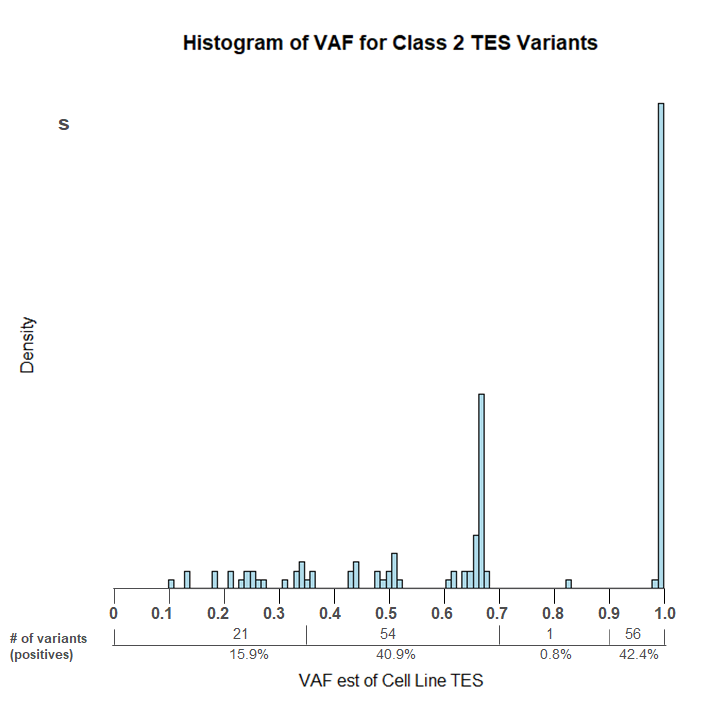

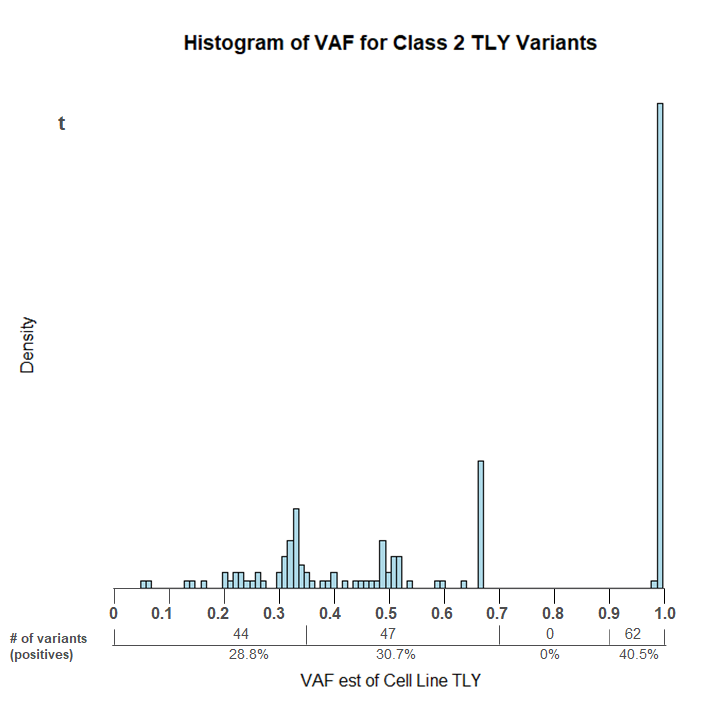


**Fig. S5: a-j) Histograms of VAF using all positives (Class 1 and Class 2) contributed by each of the 10 cell lines. Class 1 positives are >99% of all positives. k-t) Histograms of VAF using only Class 2 positives contributed by each of the 10 cell lines.**


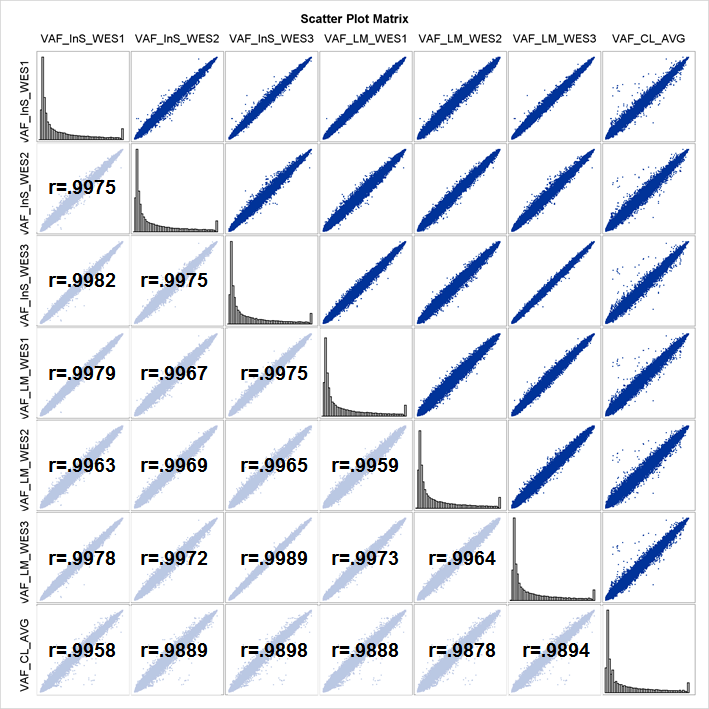


**Fig. S6: Concordance in VAF of Pooled Sample A between bait captures, *in silico* results, merged-BAM library results and the averaging of individual cell line results. Results between same kits are slightly more highly correlated. Cell line average results are concordant except for a small number of variants, many related to CNA influences from individual cell lines.**

**Fig. S7: Concordance of putative positives (variants) from CTR using WES1-3. The area of each figure represents the magnitude of the overlap between the different kits and the different methods: directly interrogating merged-BAM triplicates of Sample A or *in silico* Sample A. We observe 96% of putative positives from the CTR region are detected in all three kits in both the merged-BAM Sample A and the *in silico* Sample A using only the single SomaticSeq pipeline. Only 0.24% are not detected by SomaticSeq in either *in silico* Sample A or merged-BAM Sample A.**


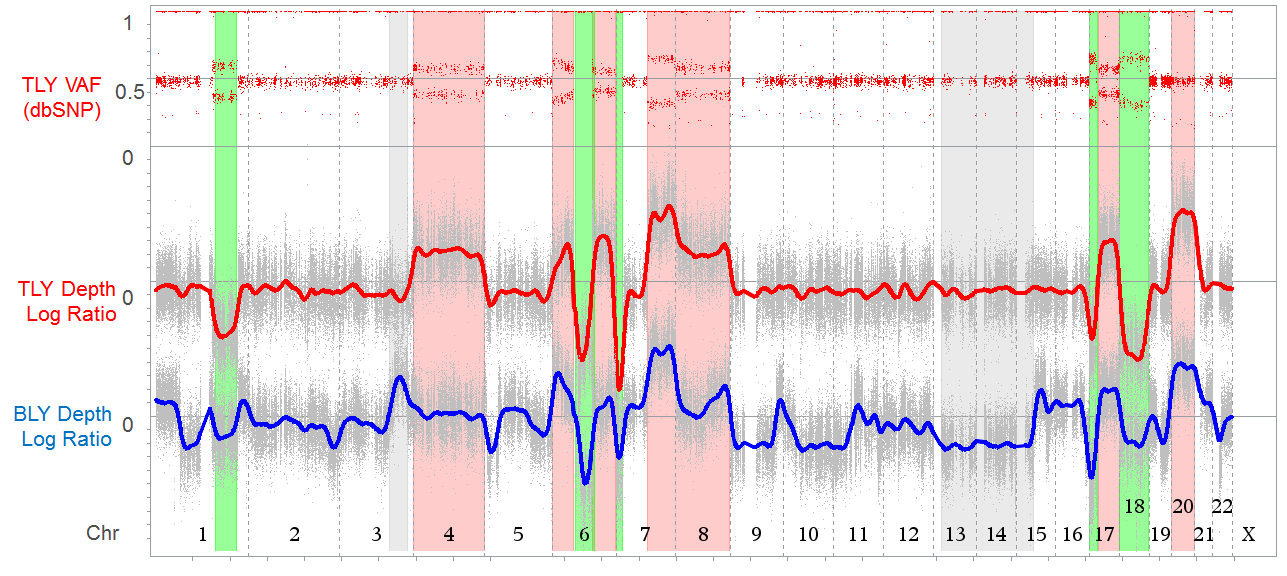


**Fig. S8: CN and other data establishing BLY as a mixture of original BLY and TLY. VAF profile at top consists of only common SNPs as well as the log ratio of TLY and BLY depths (compared to normal reference Sample B). The green regions indicate TLY somatic CN loss and the red regions indicate TLY somatic CN gain. Grey regions are somatic CN gain or loss examples that only exist in BLY and thus must have come from the original B lymphocyte cell line. All TLY gains and losses clearly appear exactly in BLY except for the gain in chr4. Thus, the original B lymphocyte cell line must have a corresponding chr4 loss, which was verified by subsequent CNV analysis of the original B lymphocyte cell line (data not shown).**


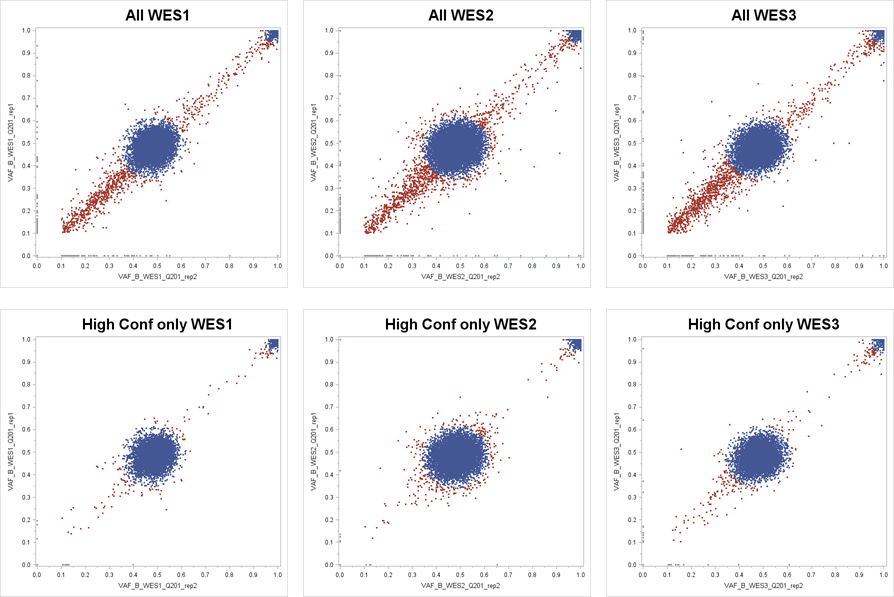


**Fig. S9: Each point is a putative variant either associated with canonical het and homozygous non-reference alleles (blue), putative variants with replicated non-zero VAF but outside canonical regions (red), or putative variants observed in only one replicate (grey). The replicate VAF of the Normal B Reference cell line is shown for the Q201 pipeline for all variants in coding regions (top) and then only those in high-confidence (HC) coding regions (bottom) by capture kit. It is expected that the Normal B Reference will only have canonical het and homozygous non-reference alleles, which is more compatible with the graphs illustrating variants from HC regions only. The red and gray positions, indicating a deviation from canonical states, range from 17% to more than 21% of putative variants over the three WES kits when outside HC regions. However, for HC-only regions, 97.8% to > 99% of the putative variants are in blue representing expected VAF for canonical alleles. Observed variation of VAF near 0.5 and 1.0 is due to stochastic sampling and sequencing error.**


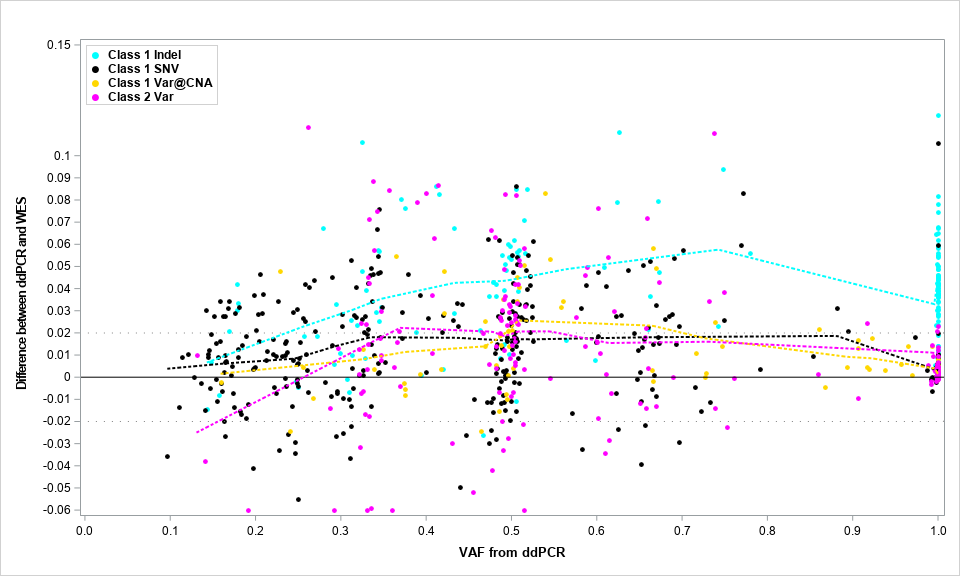


**Fig. S10: As a supplement to Fig. 3a, this figure illustrates the difference observed in VAF between ddPCR and consensus WES results across a range of VAF and different types of variants tested by ddPCR. One can see the bias in the VAF estimate from WES and how it is more pronounced at higher VAF values and for indels vs. SNVs.**

**Fig. S11: Results from a series of simulations using 2 to 12 independent unrelated cell lines from 13 cell line candidates and simulating VAF and number of variants using a mixture of a random subset of them. The yellow line indicates the median of the typical VAF given the various mixtures of cell lines. The orange dashed lines indicate the median of the 25^th^ (Q1) and 75^th^ (Q3) percentiles of the VAF given the various mixtures of the cell lines. The solid blue lines indicate the median total coding variants that one could identify in the CTR while the dashed blue line indicates the typical (median) total number of coding variants that one expects to have an allele frequency less than 10%. Corresponding values observed for Sample A are also shown.**
