# Supplementary material for: A verified genomic reference sample for assessing performance of cancer panels detecting small variants of low allele frequency
Source: Genome Biol. 2021 Apr 16;22:111. doi: 10.1186/s13059-021-02316-z (PMC8051128; doi:10.1186/s13059-021-02316-z)
Supplement: Supplementary file 3 — Additional file 3. Supplementary information. Bias in reported WES allele frequencies. Recommended process for reference sample creation. [file 13059_2021_2316_MOESM3_ESM.docx]

**Supplementary Information**

**Bias in reported WES allele frequencies**

From the ddPCR results we see in Fig. 3a and Fig. 3b, we investigated whether the allele frequency for ddPCR results were systemically higher than that observed by WES. Additional file 2: Fig. S10 illustrates (using data from Fig. 3a) the difference in ddPCR-based VAF and the consensus WES-based VAF on the Y-axis and the average VAF between them on the X-axis. One easily observes that the VAF for ddPCR is much larger especially when the VAF approaches .50 (50%) and continues as a rather constant difference of roughly 2% in absolute VAF value for SNVs in a range of VAF [0.35, 0.9]. Alternatively, the distinction for indels is even larger, when many variants achieving a 5% greater VAF (in absolute terms) from ddPCR vs. WES estimates. There are possibly multiple contributors to this bias with reference mapping bias as a likely primary contributor. We note the WES of individual cell lines typically has a VAF mode of 0.48 (consistent with the bias of 0.02 of the large majority of SNVs as seen in the Additional file2: Fig. S10) while the WGS mean VAF for hets observed in Sample B (a normal cell line) was slightly higher at 0.488.

**Recommended process for reference sample creation**

There are two straightforward requirements in developing a reliable reference sample that is useful for assessing the sensitivity, accuracy and limit of detection of oncology-related genomic variant panels: i) the reference sample(s) should contain a large number of variants with low VAF and ii) the reference sample should be available in large quantities.

The use of well-characterized cell lines that are pooled has several advantages over individual tissues or synthetic materials as a shared multi-use reference sample. Human tissues, especially cancer biopsy samples, are available in relatively small amounts and are often preserved in formalin (which compromises quality of the DNA). As discussed previously, a key limitation of current synthetic reference materials is that they contain only small number of variants at relevant VAF, making overall recall and precision determinations imprecise. In contrast, tissue-cultured cells can be grown at large scale in strictly controlled environments that will minimize any batch-to-batch variability, importantly allowing reproducibility as well as more flexibility in creating a reference with hundreds to thousands of variants at desired VAFs. One can also create a single very large batch that will last for several years for a large user base or research community.

The selection of the number of cell lines to use in a pooled approach is important. Using only a few cell lines may provide relatively few variants at low allele frequencies (e.g., less than 10%) in the mixture. Alternatively, employing more than 20 cell lines may dilute most somatic variants and many germline variants to an undetectable level while creating more overhead and complexity to the process. This implies that a “sweet spot” of between 5 and 15 pooled cell lines would be ideal for the primary reference sample. To illustrate this, we conducted a simulation study using ten of our cell lines (omitting BLY due to its shared content with TLY but including Sample B plus five other unrelated NIST normal cell lines (HG001, HG003, HG004, HG006 and HG007) by randomly selecting up to 100 collections of 2, 3, 4, …, 14 cell lines, mixing them numerically, and then determining the number of CTR-related variants for each mixture and their VAF distribution and summarizing these key parameters over the (up to) 100 simulation runs. Additional file 2: Fig. S11 indicates that when one admixes 10 cell lines, one should expect the reference sample to contain more than 20,000 variants (or more than half of known variants) with an allele frequency less than 10% in the CTR given cell lines of this kind, or more than 1 low frequency coding variant per gene on average.

Choosing cell lines from diverse population groups will increase the total number of variants and variants at low frequency for a given number of cell lines for obvious reasons. One can also see this effect in Additional file 2: Fig. S11 by noticing a jump in the median number of variants of low frequency when the number of cell lines admixed is above nine. This is partly due to nine of the 13 cell lines available being from individuals of European descent while one is of African descent, one is of Asian descent, and one is of Ashkenazi descent. By requiring the admixture of 10 cell lines from diverse populations in the simulation, one guarantees that variants from distinct populations will be mixed leading to more total variants and variants at low frequency (since they tend towards less overlap or commonality, thus they are more diluted in VAF).

Due to intratumor heterogeneity as well as the growing diagnostic utility of liquid biopsies, it is important to also identify variants with VAF less than 1%. Diluting the variants with DNA from a normal cell line, possibly from a distinct population source (to reduce variant commonality between them), is an effective means to transform variants to still lower VAF ranges.

To maximize the number of variants with a wide range of VAF in one reference sample, we selected several diverse cell lines derived from distant individuals. These cell lines represented a variety of cancer tissues and were also carefully selected to compose the Universal Human Reference RNA.^.^ Since these cell lines represented several major cancer types (carcinoma, sarcoma, blastoma, myeloma, leukemia, lymphoma and melanoma) they became ideal candidates for a pooled DNA reference sample containing both germline and somatic variants. The variety of genotypes and cancer types in Sample A provide a much larger number of variants than one cell line in total and at lower VAF. Further, we used a very well-characterized cell line from a normal individual (Sample B) to dilute the VAF in the majority of variants in reference Sample A to create reference samples C(1:1), D(1:4), and E(1:24).

For Sample A, the n=10 cell lines were mixed in equal molar amounts so that the resulting allele fraction for most individual homozygous variants unique to a cell line would be around 10% (or 1/n) and heterozygous variants around 5% (or 1/n/2). We chose to fully vet via NGS each individual cell line in the pooled reference to determine a relevant set of putative positive variants that should be in the pooled reference. Detailed examination of individual cell lines allows for identification of potential issues such as cell line contamination, pooling errors, or pooling imbalances. Screening the individual DNA sources can also aid discernment of similar vs. dissimilar sources of DNA, allowing the pooled reference to contain the highest number of non-overlapping variants with the fewest number of cell lines.

However, our analysis has also shown that one can possibly perform a small screen to identify the population group of the individual cell lines or DNA sources, create a pool from these cell lines, and then deeply sequence only the final pooled reference using a large input amount of DNA. The concordance between variants detected by sequencing each cell line and then pooling the results vs. sequencing the pooled reference alone was extremely high (>99%). This implies that sequencing the final reference sample is the only sequencing step absolutely required. Also, the pooled reference provides the best estimate of the final VAF of the pool for binning performance by VAF as the pool naturally subsumes positional-dependent depth variation across cell lines (due to variation of CNA and general aneuploidy across individual cell lines).

Even though we had tremendous success confirming our variants with orthogonal assays (ddPCR), we recommend selecting a useful collection of both positive and negative candidates (perhaps 50-100 of each) and orthogonally assaying the final reference sample and individual cell lines/DNA sources to ensure there are no obvious discrepancies between the two. Performing an orthogonal validation of this type ensures that admixtures were performed properly and provide confidence in the reference sample and related analysis.

Finally, the quality of the DNA reference sample is very important. Since many NGS technologies required high molecular weight DNA, it is critical to use DNA extraction and purification methods that allow isolation of high-quality DNA. Stringent quality control (QC) of DNA isolated from each individual cell line is necessary before the pooling. The concentration and purity of DNA isolated from individual cell lines should be verified by measuring the A260/280 and A260/230 absorbance ratios on a spectrophotometer. The concentration of the double stranded DNA and the DNA size and integrity should accurately quantified. DNA samples with DNA Integrity Number (DIN) of 8.0-10 are highly intact and are recommended to be in this range.
