# Supplementary material for: A verified genomic reference sample for assessing performance of cancer panels detecting small variants of low allele frequency
Source: Genome Biol. 2021 Apr 16;22:111. doi: 10.1186/s13059-021-02316-z (PMC8051128; doi:10.1186/s13059-021-02316-z)
Supplement: Supplementary file 4 — Additional file 4. Review history. [file 13059_2021_2316_MOESM4_ESM.docx]

Review History

**First round of review**

**Reviewer 1**

**Are you able to assess all statistics in the manuscript, including the appropriateness of statistical tests used?** Yes, and I have assessed the statistics in my report.

**Comments to author:**

In this study, the authors generated a set genomic reference materials to provide a standard in assessing performances of somatic mutation detection, especially with low allele frequency. By mixing 10 cancer cell lines with detected somatic/germline mutations whole allele frequencies are various, the authors could generate a mixture (sample A) with >25,000 positive mutations and >10,000,000 negative sites. The presence of the positive mutations and was validated by ddPCR of a random subset (~1%). The authors further generated a diluted version (sample C,D,E), by adding a normal reference sample (sample B) to sample A in three different ratios (1:1, 1:4 and 1:24). Finally, the author stated that the sample C-E contain confirmed mutations with low VAFs (as low as 0.02%) that can present rare mutations to test for liquid biopsy.

In general, this is an essential and timely study. Unlike germline mutations, such as the Genome In A Bottle, reference standards for somatic mutations are very limited. Only a few commercial products are available, but with a limited number of control positives and negatives. Detection of somatic mutations, especially with low allele (VAF<5%) frequencies is considered difficult. Although there are many different methods, their accurate performances have not been measured in a standard way. And their applications including cancer panel, should be used with care as any false positives/negatives may raise serious problems to patients. I appreciate the authors' efforts to construct one. On the other hand, I have several concerns in a technical aspect including study design that must be resolved before publication.

Major concerns:

1. The main contribution of the study should be clarified. From the title, readers expect the main contribution to be the standard genomic reference itself, which could be available for public as a form of the material or the sequenced data. However, in page 4, the authors wrote "This study provides a guideline that delineates the steps one can follow for developing, validating, and implementing new genomic reference material as well as describing the products of our effort". And significant amount of the remaining manuscript describes the guideline and recommendations for it. While both are somehow important, the guideline is of less priority as the most of the ideas are not new, superficial and can be easily drawn. This study should be more focused on the material, such as (1) how much the provided material is robust, reproducible and sustainable, (2) how the sequenced data can be used when the material is not open to the public, and optionally (3) showcase how the existing pipeline (such as the GATK's best practice) is tested on the material/data. So the final contribution can be the wider use of the developed material in the research community as in the case of GIAB.

2. In addition to 1, the sequence data must be publicly available at least before the next round of review.

3. The authors used 10 different cancer cell lines. However, cancer cell lines are mutable and rapidly evolving, so batches from different labs are highly heterogeneous (please refer to Liu et al, Nat. Biotech. 37:314-322, 2019). Therefore, newly constructed 'sample As' from different lab would not be consistent with the authors' sample A, generating discrepancies in the sort and the amount (allele frequencies) of the mutations. This is again why the accessibility to the material/data is more important than the guideline.

4. In the identification of positive mutations of the 10 cell lines, the authors aimed to achieve high sensitivity to detect down to 10% VAF, so the mixture (sample A) can contain ~1% VAFs. Any mutations at such VAF in the original cell line means either (1) the copy number of the wild copy is increased or (2) the mutation is heterogeneous in the batch. If the (1) is the case, the site is may be unstable for copy number, and may be not appropriate to be a reference standard. If the (2) is the case, more seriously, mutations in any other batches may have different VAFs, or even do not exist. The authors should provide a rationale for the use of those mutations.

5. The presence and the VAF of the mutations are not validated in sample C, D, E. For example, there can be interferences among two or more cell lines in the mixture such as multi-allelic positions, acquisition of loss of control positives and negatives. Additional analysis on sample C, D, and E can deliver more information including final VAFs, artifacts in the mixture process, and the detectability of the loss AF mutations (whether they are truly distinguished from negative sites).

6. The main text should be improved. Introduction is abnormally long and contains many different themes. I think Introduction could be more succinct by focusing on the main problem (background, importance, brief introduction to the approach). There have been many previous studies to construct standard references using various approaches (Suzuki et al, Genes and Environment, 2020, Craigs et al, Sci Rep, 2016;6:24607, Kim et al, Nat Communs, 2019:10,1047). Detailed description of the consortium can be reduced or removed. On the other hand, essential information is missing in Results, so the readers must refer to the Supplements. Results must include all the essential information and should be understandable without supplementary information.

Minor concerns:

1. Please provide the exact numbers or ranges instead of approximates wherever possible in the main text. Below is a few of them

- line 101, "an average depth of 200x"

- line 130, "we added several hundred identified variants"

- line 136, "a large set"

- line 157, "more than 100 low frequency variants"

- line 159, "nearly 300 positive"

- line 161, "more than 99%",

2. In line 162, what is the "a comparable VAF value"?

3. In line 49, the meaning of "variants at several levels" is unclear

4. In page 67, "This is reasonable-" the authors explained why a mixture of germline variants is a proper way to construct somatic reference. I mostly agree. However, some pipelines apply hard filters to remove known germ line positions. This unwanted effect can be discussed.

5. In line 147, please explain undefined terms such as 'merge-BAM'

6. Figure 2 is neither quantitative nor scaled but only schematic and the color code is confusing

7. In Figure 4, class 1 and 2 variants are not marked in the illustration.

8. In line 86 of Supplementary Material, what are the "certain individual cell lines?"

9. Color legends should be added to Supplementary Fig 9 and 11.

10. In supplementary table 1, the name of sequencing technology should be written for better readability.

11. In supplementary table 12, sample name should be identified.

**Reviewer 2**

**Are you able to assess all statistics in the manuscript, including the appropriateness of statistical tests used?** Yes, and I have assessed the statistics in my report.

**Comments to author:**

This manuscript describes the first potential reference sample that is characterized for both sensitivity and specificity for a focused set of genes at a wide variety of VAFs. This is a noble and ambitious goal, and I expect both the methods will be useful for the community, and the samples will be quite useful to the community as well assuming the authors can make their data public, which is one of my biggest concerns. The manuscript represents a large amount of work, and some of the choices made could be described more clearly, and the limitations are important to make clear as well, since no benchmark is perfect. I expect this will be a valuable resource after addressing the points below.

1. In the title, the authors call this "A Validated Genomic Reference Material". This would more accurately be called "A Genomic Reference Sample". The term "Reference Material" has the precise definition "Material, sufficiently homogeneous and stable with respect to one or more specified properties, which has been established to be fit for its intended use in a measurement process" in ISO GUIDE 30:2015. It is not clear that this work demonstrates the material is sufficiently homogeneous and stable, so the term "reference sample" should be used instead of "reference material" throughout the manuscript. Similarly, the word "validated" is typically used with methods rather than reference materials, so should be removed.

2. This work uses "ten cancer cell lines historically used to create RNA for the Agilent Universal Human Reference (UHR) RNA material10 …. a cell line derived from a normal male individual (Agilent OneSeq Human Reference DNA, PN 5190-8848)". Proper informed consent has historically been a challenge with developing genomic reference samples from cancer cell lines. Many cell lines have no consent or limited consent, such that genome sequencing data cannot be made publicly available. The enduring value of this reference sample could be substantially limited if no consent was given for public dissemination of the data.

3. What is the distribution of distances between variant calls after mixing all the cell lines, and do the authors expect artificially nearby variants could cause problems for variant callers?

4. There appears to be a strange issue with the BLY cell line being mixed with TLY, which is not even mentioned in the main text except for a statement in Table 1. Could the authors please clearly state this issue in the main text and its ramifications for the results?

5. The authors state "To protect against a common bias towards false positive calls, the variant calls were filtered based on NIST high-confidence regions2 as well as identified low genomic complexity regions28 that resulted in a consensus target region (CTR) as shown in Fig. 2". The version of NIST high-confidence regions (more properly termed "NIST benchmark regions") is not specified anywhere, so please add this. Since they use HG002-HG005, it appears they are using the regions published in reference 95, which should be added here.

6. The authors use the NIST high-confidence regions to filter errors in their calls, and others have used them in this way also, but it is important to note that this is not their intended use, and there are limitations to using them in this way. Specifically, these are sample-specific and were intended to be used in conjunction with the corresponding benchmark vcf to identify true positives, false positives, and false negatives, when comparing a query vcf (see ref 95 and https://www.nature.com/articles/s41587-019-0054-x). In practice, intersecting the v3.3.2 bed files for HG002-HG005 will remove most seg dups and other difficult regions, and others have used it in this way also, but it would be good to state the limitations, clearly state the version, and make clear that this approach is pretty specific to the v3 benchmark and probably wouldn't work for the v4 benchmarks that use long reads.

7. It's important that the authors share their results publicly for evaluation if they want them to be accepted by the community. I did a quick intersection of the known positive variants with a homopolymers bed file from GIAB/GA4GH (ftp://ftp-trace.ncbi.nlm.nih.gov/ReferenceSamples/giab/release/genome-stratifications/v2.0/GRCh37/LowComplexity/GRCh37_AllHomopolymers_gt6bp_imperfectgt10bp_slop5.bed.gz) and found a few questionable variants that might arise from systematic errors in PCR (e.g., chr1:6257784, chr11:118773097, and chr1:115053498. The authors may want to remove this homopolymer bed from their variants and CTR.

8. How did the authors decide to use these rules? "Within the CTR, a set of known positive variants (termed Class 1 variants/positives) were identified as being called in the majority of the consortium pipeline-library combinations for at least one cell line in each replicate of WES1, WES2, and WES3 (the replicated kits). For more details, see "Rules for determining positive variants" in the Laboratory and Bioinformatics Methods section."

9. THe authors state "To further enrich the set of identified variants, we also examined each cell line using the WGS1 linked-read method in COSMIC genes. We added several hundred variants (termed Class 2 variants/positives) to our set of Class 1 variants that were outside our CTR region but were in high- confidence coding regions of COSMIC genes. The Class 2 variants were included only if they were detected by WGS1 in at least one cell line by two different pipelines and detected by at least two WES kits. Using hg19, we identified 42,570 Class 1 and Class 2 variants in Sample A (28,064 having VAF less than 20%) with additional characteristics of these variants provided in Supplemental Table 4."

This is a relatively small number of extra variants, and this linked read method will have many of the same PCR-related biases, e.g., in homopolymers and tandem repeats. Have the authors looked at these variants to determine they are accurate?

10. The authors should clearly state the limitations of this approach: "Pile-ups of each cell line were also examined to identify a large set (more than 10,000,000) of negative variant loci that were then assimilated into a collection of loci that would be absent any variant in the pooled Sample A (see "Rules for determining known negative variant positions"). Negative loci for pooled Sample A imply that if there is any somatic variant at that loci, it must have an observed AF at less than 0.25% in the pooled Sample A for each replicated kit." For example, this will exclude many places where there are systematic errors in targeted sequencing, so that methods will appear to perform better than they are. Also, they do not address challenges in representation of variants, where complex variants (nearby snps and indels) can often be represented in many different ways, e.g., at different locations within a homopolymer or tandem repeats (see https://rdcu.be/bqpDT )

11. The authors should perform statistical tests or give confidence intervals to support several of the claims made in this section: "Using SomaticSeq, we confirmed greater than 99.5% of the Class 1 and 2 variants identified from the individual cell line analysis were at or near the expected VAF in Sample A using either an in silico version of Sample A or the merged-BAM Sample A (see Supplemental Table 6). In addition, although the WES4 Thermo Fisher runs had only one replicate per cell line and were not as deeply sequenced as WES1-3, over 97.8% of the CTR positives with Sample A VAF > 5% were detected in at least one cell line. Additional information on WES4-related sensitivity and precision of individual cell lines are provided in Supplemental Fig. 2."

12. The authors state: "Finally, we used droplet digital PCR (ddPCR) technology to orthogonally validate a large number of positives and negatives from different classes of variants in individual cell lines, reference Sample A, and normal Sample B. We also orthogonally tested those same variants using admixture reference samples C, D and E where Sample C is a 1:1 dilution of A:B, Sample D is a 1:4 dilution, and Sample E is a 1:24 dilution. The validated variants included more difficult-to-detect variants such as 50 smaller insertions-deletions (indels) and more than 100 low frequency variants (VAF ~ 1-5% in pooled Sample A) as well as 50 variants randomly sampled from the larger Class 2 positive set derived from COSMIC genes. In total, we identified nearly 300 positive and 39 negative loci within our known positive and negative set for independent ddPCR testing (Supplemental Table 7)". Could the authors be more explicit about the variants that could not be tested by ddPCR because assays couldn't be designed? For example, are there variants in difficult to map regions or homopolymers or tandem repeats that failed design?

13. The authors state: "We recommend the use of tissue-cultured cells grown in a large batch in strictly controlled environments that will minimize any batch-to-batch variability and allow use in the community for several years." Did they actually do this for this work?

14. The authors state: "Reasons for these differences include HG001 receiving a higher initial degree of manual review as well as more time related to review (HG001 was the first specimen having this data released publicly). We chose a more conservative approach for ground truth and created a common high confidence region based on overlapping regions of HG002, 003, 004, and 005 to serve as the basis for high confidence regions for this initial release of positives and negatives. In addition, we excluded a small amount of low complexity regions28 overlapping with this more universal high confidence region which served as the basis for both Class1 and Class 2 variant detection." It isn't exactly true that the differences for HG001 result from manual review. It is more related to the greater amount of data for HG001 available at the time (now HG002 has more data in https://doi.org/10.1101/2020.07.24.212712), and because it included chrX if the authors included this.

15. The authors state: "Variants from WGS 10X Chromium libraries of individual cell lines were examined in high confidence coding regions of COSMIC genes but outside of CTR regions that includes common design coding regions to all WES kits utilized in this study. If the same variant was called by two different WGS variant callers (Freebayes and GATK) and was called by SomaticSeq from two different WES kits in the same cell line, then the variant was categorized as a Class 2 positive variant." What aligner did they use, and why didn't they use the LongRanger mapper and variant caller?

16. The authors state: "Any positions with total DP >= 500, ADP > 2 and AF >= 0.002 were removed. " Is there a statistical basis for this, e.g., confidence interval for max allele frequency?

17. The authors state: "The adjacent positions from both end of the INDEL calls were also removed." Why only the adjacent positions? As stated above, different representations of indels can cause many problems for this. GIAB now excludes 50bp from either end of an uncertain variant plus any overlapping tandem repeats or homopolymers (https://doi.org/10.1101/2020.07.24.212712).

18. The authors state: "While some models appeared more reasonable than others, we observed large variations in the estimates for the cell line mix ratios for different models and subsets, resulting in fundamentally unreliable VAF predictions. The instability in estimates was possible due to the large number of rearrangements in the cancer cell lines, creating inconsistent depth at a given locus from cell line to cell line. A simple linear regression of the VAF of each cell line onto the Sample A VAF provided reasonable if oversimplified results that indicated the cell lines were properly mixed (given the approximate 1:2 mixture of TLY into BLY described elsewhere)." The different ploidies of the cell lines is a major limitation that should be highlighted very clearly by the authors, since this could cause problems for some sequencing technologies as well as for the mixture designs.

19. The authors state "One easily observes that the VAF for ddPCR is much larger especially when the VAF approaches .50 (50%) and continues as a rather constant difference of roughly 2% in absolute VAF value for SNVs in a range of VAF [.35, .9]. Alternatively, the distinction for indels is even larger, when many variants achieving a 5% greater VAF (in absolute terms) from ddPCR vs. WES estimates. We conjecture that this bias is due to capture baits as 1)	capture baits are typically designed from reference sequence, 2)	the magnitude of the difference for indels was observed to be related to the bases inserted or deleted (data not shown), implying an interaction between the difference in detected sequence vs. reference sequence and the magnitude of that deviation from expected VAF, and 3)	we do not observe a bias when examining WGS results and specifically the het state of normal cell lines while the WES results yield VAF estimates from those same cell lines indicating the mode for hets is typically around .48 (rather than .50) across kits and cell lines." Did the authors have sufficient depth from WGS to make this claim? I would have expected this is a simple mapping bias for indels, which has frequently been reported.

20. The authors state: "Important data items from the consortium reference material effort that we intend to disclose…". As stated above, I am concerned that these samples aren't properly consented for these data to be made public

21. Could the authors add if this is a potential Agilent product to "Natalia Novoradovskaya, Katherine Wilkins, Anne Lucas, Raymond Miller, Carlos Pabon are all employees of Agilent Technologies, Inc."?

22. In Table 1: Please give more details for this: "CNA were estimated using Agilent GenetiSure Cancer Research CGH+SNP Microarray (2x400K), G5975A and WES."

23. In Table 2, specify whether this is just Class 1 or Class 1&2

24. In Figure 2, change "NIST high-confidence regions" to "NIST v3.3.2 benchmark regions", specifying the version and the current terminology, which is important for understanding the results

25. In Figure 3a, could the authors clarify why n=1242 when they only had 375 ddPCR assays?

26. In Figure 3, it would be useful also to have histograms of the VAF from WES for all class 1 and all class2 variants, both in the cell lines and in Sample A.

27. In Figure 4, why are there very few "Known Negatives" in the first 2 exons?

**Authors Response**

**Point-by-point responses to the reviewers’ comments:**

Reviewer #1

1. The main contribution of the study should be clarified. From the title, readers expect the main contribution to be the standard genomic reference itself, which could be available for public as a form of the material or the sequenced data. However, in page 4, the authors wrote "This study provides a guideline that delineates the steps one can follow for developing, validating, and implementing new genomic reference material as well as describing the products of our effort". And significant amount of the remaining manuscript describes the guideline and recommendations for it. While both are somehow important, the guideline is of less priority as most of the ideas are not new, superficial and can be easily drawn. This study should be more focused on the material, such as (1) how much the provided material is robust, reproducible and sustainable, (2) how the sequenced data can be used when the material is not open to the public, and optionally (3) showcase how the existing pipeline (such as the GATK's best practice) is tested on the material/data. So the final contribution can be the wider use of the developed material in the research community as in the case of GIAB.

*Response: We agree with the reviewer’s comments and have completely moved the process and recommendations for creating a reference sample to the supplementary material. The revised main part of the manuscript is focused on the reference Sample A itself (with ancillary discussion of its companions B, C, D, and E). We have discussed how the DNA from these samples can and are being used to evaluate cancer and liquid biopsy panels. While not a primary point of the study, we have added a table and described in the Results section how particular well-known pipelines such as GATK, Mutect 2, Sentieon TnScope, and SomaticSeq (Supplemental Table 6b) performed relative to sensitivity in the individual cell line results. We discuss that Agilent plans to make the DNA from Sample A available to the public (Sample B is already available). Additional particular responses to items (1)-(3) follow:*

(1) how much the provided material is robust, reproducible and sustainable

*Response: A single batch of DNA material once produced is easily reproducible in its various aliquots and the results of the VAF profile comparisons in Supplemental Figure 8 whether examining in silico A or Merged-BAM A from 3 different kits from 3 different laboratories testify to the reproducibility of those results. There is the remaining question of sustainability. However, we do not assume that the next version of the DNA reference sample will be identical nor is it necessarily required that the next version of the reference sample be identical in makeup. It is required that each version be comprehensively tested and documented so that its contents (positives and negatives) and characteristics (VAF, complex variant situations) are known and documented/published. We have indicated the means to do that on a much smaller scale than the consortium-level analysis within this manuscript product (i.e., the supplemental information) given the lessons learned.*

(2) how the sequenced data can be used when the material is not open to the public,

*Response: We have addressed the utility of the sequenced data in the General Response earlier. We should note even at the risk of being repetitious that the sequence results and conclusions from it were used to evaluate cancer panels and liquid biopsy panels in other submitted companion papers. We would not have had as comprehensive an evaluation without this reference material.*

(3) showcase how the existing pipeline (such as the GATK's best practice) is tested on the material/data.

*Response: While not a “showcase”, we have added sensitivity results from selected pipelines and kits in Supplemental Table 6b by cell line to illustrate the evolution of callers. We show the performance of GATK’s original haplotype caller along with Mutect2 and SomaticSeq on selected library kits. Mutect2, SomaticSeq and Sentieon TNscope all performed very well along with some other pipelines. We have emphasized SomaticSeq primarily because it performs very well and is open-source.*

2. In addition to 1, the sequence data must be publicly available at least before the next round of review.

*Response: We agree. This was always the intent. As described in the General Response, the data is now available in a public repository:* [*https://dataview.ncbi.nlm.nih.gov/object/PRJNA673156?reviewer=kh0sk792c0jgvjssa6h070ma4k*](https://dataview.ncbi.nlm.nih.gov/object/PRJNA673156?reviewer=kh0sk792c0jgvjssa6h070ma4k)*.*

3. The authors used 10 different cancer cell lines. However, cancer cell lines are mutable and rapidly evolving, so batches from different labs are highly heterogeneous (please refer to Liu et al, Nat. Biotech. 37:314-322, 2019). Therefore, newly constructed 'sample As' from different lab would not be consistent with the authors' sample A, generating discrepancies in the sort and the amount (allele frequencies) of the mutations. This is again why the accessibility to the material/data is more important than the guideline.

*Response: This issue was primarily addressed in the General Response. Agilent will not be releasing the individual cell lines. Agilent will handle this reference in a manner highly similar to their Universal Human RNA Reference which is from the same set of cells. They will infrequently make a large batch of cells, isolate and mix the DNA, and provide aliquots. Each iteration of Sample A will be documented as to its “delta” relative to the previous iteration, thereby addressing the issue of consistency going forward.*

4. In the identification of positive mutations of the 10 cell lines, the authors aimed to achieve high sensitivity to detect down to 10% VAF, so the mixture (sample A) can contain ~1% VAFs. Any mutations at such VAF in the original cell line means either (1) the copy number of the wild copy is increased or (2) the mutation is heterogeneous in the batch. If the (1) is the case, the site is may be unstable for copy number, and may be not appropriate to be a reference standard. If the (2) is the case, more seriously, mutations in any other batches may have different VAFs, or even do not exist. The authors should provide a rationale for the use of those mutations.

*Response: We agree that in either of these situations that monitoring somatic changes and/or heterogeneity in individual cancer cell lines over time makes for additional difficulty in precisely determining in time the variant contents and characteristics of a collection of cells from one purported cell line. However, as indicated in the General Response, that is not the design of our study nor is it the design of the intended use going forward. The immediate remedy is simply to prepare a very large batch of gDNA from the cell lines. Agilent estimates that the new version would be a batch of approximately 50 mg that would be sufficient for 10 years. Even though there will undoubtedly be some changes in variants (pos and neg) and VAF in the next version, most should be highly similar as most variants are germ line and not under heavy CN influence across the cell lines. Therefore, we already have a tremendous head start on the characteristics of the next batched version that would require much less testing to confirm, given the lessons learned from this study. In any case, this version is being used now for several companion papers related to this study to evaluate cancer and liquid biopsy panel performance as well as evaluate control material techniques. It is also being trialed as a reference sample in actual laboratories testing cancer panels for CLIA certification. The utility is that one can use a reference sample (pool) like this to properly determine basic characteristics such as LOD and other performance characteristics (recall at different VAF ranges, false positive rates) very quickly without having to test 100+ “real” tumors of possibly unknown contents where each result requires a custom PCR assay to confirm. That is, this type of reference sample may be able to greatly simplify certain aspects of genomic assay validation so long as access to additional aliquots for a reasonable period of time is possible. That is the plan.*

5. The presence and the VAF of the mutations are not validated in sample C, D, E. For example, there can be interferences among two or more cell lines in the mixture such as multi-allelic positions, acquisition of loss of control positives and negatives. Additional analysis on sample C, D, and E can deliver more information including final VAFs, artifacts in the mixture process, and the detectability of the loss AF mutations (whether they are truly distinguished from negative sites).

*Response: A large collection of putative variants (284 positives and 39 negatives, 114 of which were selected at random) were orthogonally validated by ddPCR in admixtures C, D, and E as illustrated in Fig 3c. All VAF values for those variants in the admixtures C, D and E showed expected values within stochastic sampling given the admixture level relative to Sample A and given that the variant was not also present in Sample B (A and B are the two samples used for admixture). We discussed that germline variants from Sample B could interfere with variants in Sample A (which is why we provide the contents of Sample B also). I should note that potential interference in detection of variants is already present in Sample A itself, as it is an equimolar admixture of 10 cell lines. Admixing A with B simply makes the composites C, D, and E to be a product of pooling 11 cell lines. Also, since B is a normal sample, the variants in A impacted by B are readily discernible as the variants of B are easily detected relatively (hets and homozygous non-reference positions with minimal CNA). The detection challenge of variants within C, D and E is one of low frequency. Otherwise, sample A has essentially equivalent interference as C, D and E. In summary, we do not deny the potential for interference. We have documented in the manuscript (in various sections) different types of interference including multi-allelic SNVs and the interference of B with A in the admixtures C, D and E.*

Reviewer #1 (Minor)

1. Please provide the exact numbers or ranges instead of approximates wherever possible in the main text. Below is a few of them

- line 101, "an average depth of 200x"

- line 130, "we added several hundred identified variants"

- line 136, "a large set"

- line 157, "more than 100 low frequency variants"

- line 159, "nearly 300 positive"

- line 161, "more than 99%",

*Response: The manuscript has been edited as requested and exact numbers were used for these instances and others.*

2. In line 162, what is the "a comparable VAF value"?

*Response: This line has been edited for clarity. “…100% of the variants from the different classes were verified as positives with 99.65% having a concordant VAF value (i.e., within stochastic sampling noise) when comparing ddPCR and WES consensus VAF estimates…”*

3. In line 49, the meaning of "variants at several levels" is unclear

*Response: We have adjusted wording to say "VAF magnitudes" rather than "VAF levels*

4. In page 67, "This is reasonable-" the authors explained why a mixture of germline variants is a proper way to construct somatic reference. I mostly agree. However, some pipelines apply hard filters to remove known germ line positions. This unwanted effect can be discussed.

*Response: We have added text in the Discussion related to an acknowledgement that some pipelines apply filters to remove germline positions and the potential consequence to performance metrics.*

5. In line 147, please explain undefined terms such as 'merge-BAM'

*Response: We have added additional text when it is first mentioned to briefly describe the merged-BAM file and we have created Methods Sections to describe merged-BAM A and in silico A in more detail.*

6. Figure 2 is neither quantitative nor scaled but only schematic and the color code is confusing

*Response: We have revised Figure 2 for clarity. It is more like a Venn diagram which are not always scaled to magnitude of the contents of the intersecting sets.*

7. In Figure 4, class 1 and 2 variants are not marked in the illustration.

*Response: The Figure 4 legend indicates the identity of the Class 1 and Class 2 variants. There is only the one Class 2 variant in the figure. We can add notation to the graph to indicate the single Class 2 variant if desired.*

8. In line 86 of Supplementary Material, what are the "certain individual cell lines?"

*Response: This statement was removed and an updated explanation provided (along with a correction that led to greater concordance between Merged-BAM and in silico results). The correction had to do with misinterpreting output from one source. While the analyst had desired to compare results “native” to hg19, the first pass included variant results from WES1 that were initially hg38-based but lifted over to hg19. The corrected version used results based on hg19 alignment only with variant calling.*

9. Color legends should be added to Supplementary Fig 9 and 11.

*Response: Color legends have been added to Supplementary Figures 9 and 11.*

10. In Supplementary table 1, the name of sequencing technology should be written for better readability.

*Response: The sequencing technology is now more obviously shown in Supplementary Table 1.*

Reviewer #2

1.In the title, the authors call this "A Validated Genomic Reference Material". This would more accurately be called "A Genomic Reference Sample". The term "Reference Material" has the precise definition "Material, sufficiently homogeneous and stable with respect to one or more specified properties, which has been established to be fit for its intended use in a measurement process" in ISO GUIDE 30:2015. It is not clear that this work demonstrates the material is sufficiently homogeneous and stable, so the term "reference sample" should be used instead of "reference material" throughout the manuscript. Similarly, the word "validated" is typically used with methods rather than reference materials, so should be removed.

*Response: We have modified the manuscript and title to say to a ”verified reference sample” and updated related text. We have retained the term “validated” in certain contexts such as orthogonal validation of a result via ddPCR as “orthogonally validated” is a common term used to describe this practice.*

2.This work uses "ten cancer cell lines historically used to create RNA for the Agilent Universal Human Reference (UHR) RNA material10 …. a cell line derived from a normal male individual (Agilent OneSeq Human Reference DNA, PN 5190-8848)". Proper informed consent has historically been a challenge with developing genomic reference samples from cancer cell lines. Many cell lines have no consent or limited consent, such that genome sequencing data cannot be made publicly available. The enduring value of this reference sample could be substantially limited if no consent was given for public dissemination of the data.

*Response: This issue was addressed in our General Response.*

3.What is the distribution of distances between variant calls after mixing all the cell lines, and do the authors expect artificially nearby variants could cause problems for variant callers?

*Response: There are instances of variants next to each other both in and not in phase. Since the first submission, we created a companion list of just over 100 MNVs (mainly di-nucleotide variants in phase). We know that users of this reference sample are not simply examining the bulk of variants as a whole but also examining their ability to detect variants under certain conditions: variants in particular genes, SNVs vs. Indels vs. MNVs, detect variants in and outside of the CTR. Variants near each other could indeed create difficulties for variant callers, and we feel that is a reasonable challenge with which variant callers should be assessed. Since we are releasing the characteristics (location, nature of change) of all of the positive variants, users can segment the positives using different characteristics (VAF in low ranges, presence in certain genes, Indels and MNVs, etc) to assist with evaluating panels, lab and in silico methods.*

*Related to the distribution of distances, when examining that distribution, there are two primary modes: 1) mode 1 is at 100 bases and most likely reflects distance between variants within exon. 2) mode 2 is roughly 10,000 bases and most likely reflects distance within-gene or intergenic distances. Only 1.8% of positives are very close (dist ≤ 5b) and the MNVs related to a subset (1/3) of those are now documented in our positives list.*

4.There appears to be a strange issue with the BLY cell line being mixed with TLY, which is not even mentioned in the main text except for a statement in Table 1. Could the authors please clearly state this issue in the main text and its ramifications for the results?

*Response: This item is now more fully discussed in the main text Results section under “Reference Sample A Characteristics”. It was discussed previously in the Supplemental information. The ramifications are also discussed in the main text. For clarity and time related to reviewers, in our view, the ramifications for this inadvertent admixture are minimal as we used the initial individual cell line data to establish presence or absence of variants and then we only used direct measurement of Sample A to determine the final study-based VAF of Sample A for binning purposes (i.e.,which variants are in the VAF bin of 1%-2.5%, 2.5%-5%, etc.). For the fixed initial version of the Sample A DNA and its subsequent uses in the study, the admixture has essentially no impact other than the difficulty and ambiguity in labeling the BLY contribution (e.g, do we call that set the BLY cell line or the BLY-TLY admixture?). For simplicity, we have retained the original moniker of BLY.*

5.The authors state "To protect against a common bias towards false positive calls, the variant calls were filtered based on NIST high-confidence regions2 as well as identified low genomic complexity regions28 that resulted in a consensus target region (CTR) as shown in Fig. 2". The version of NIST high-confidence regions (more properly termed "NIST benchmark regions") is not specified anywhere, so please add this. Since they use HG002-HG005, it appears they are using the regions published in reference 95, which should be added here.

*Response: The version used (v3.3.2) was specified in the submitted document in the Methods Section (NIST high-confidence regions) on line 583. However, the reviewer makes a good point that this could be more obvious. Therefore, we have made additions to the manuscript in Figures, Tables and text to make it more well-known. We will add notation to indicate they are also known as NIST benchmark regions.*

6.The authors use the NIST high-confidence regions to filter errors in their calls, and others have used them in this way also, but it is important to note that this is not their intended use, and there are limitations to using them in this way. Specifically, these are sample-specific and were intended to be used in conjunction with the corresponding benchmark vcf to identify true positives, false positives, and false negatives, when comparing a query vcf (see ref 95 and https://nam04.safelinks.protection.outlook.com/?url=https%3A%2F%2Fwww.nature.com%2Farticles%2Fs41587-019-0054-x&amp;data=04%7C01%7CWendell.Jones%40q2labsolutions.com%7C23d30c62b21740fbdf5108d871e4983e%7C5989ece0f90e40bf9c791a7beccdb861%7C1%7C0%7C637384573912655943%7CUnknown%7CTWFpbGZsb3d8eyJWIjoiMC4wLjAwMDAiLCJQIjoiV2luMzIiLCJBTiI6Ik1haWwiLCJXVCI6Mn0%3D%7C1000&amp;sdata=cb%2Fu9Jj%2Bqk%2FJpyNLsJrdBsUllWWCung8XS6HeobYu%2Bk%3D&amp;reserved=0). In practice, intersecting the v3.3.2 bed files for HG002-HG005 will remove most seg dups and other difficult regions, and others have used it in this way also, but it would be good to state the limitations, clearly state the version, and make clear that this approach is pretty specific to the v3 benchmark and probably wouldn't work for the v4 benchmarks that use long reads.

*Response: Text was added in discussion that alluded to the limitations of the v3 benchmark as well as the need to update the benchmark-related data with new v4 benchmarks as they and new sequencing data of reference Sample A with long reads becomes available.*

7.It's important that the authors share their results publicly for evaluation if they want them to be accepted by the community. I did a quick intersection of the known positive variants with a homopolymers bed file from GIAB/GA4GH (https://nam04.safelinks.protection.outlook.com/?url=ftp%3A%2F%2Fftp-trace.ncbi.nlm.nih.gov%2FReferenceSamples%2Fgiab%2Frelease%2Fgenome-stratifications%2Fv2.0%2FGRCh37%2FLowComplexity%2FGRCh37_AllHomopolymers_gt6bp_imperfectgt10bp_slop5.bed.gz&amp;data=04%7C01%7CWendell.Jones%40q2labsolutions.com%7C23d30c62b21740fbdf5108d871e4983e%7C5989ece0f90e40bf9c791a7beccdb861%7C1%7C0%7C637384573912655943%7CUnknown%7CTWFpbGZsb3d8eyJWIjoiMC4wLjAwMDAiLCJQIjoiV2luMzIiLCJBTiI6Ik1haWwiLCJXVCI6Mn0%3D%7C1000&amp;sdata=j%2BVMTDabC1ywTE4SPMwTRb8ogwzjBuyBlaZV8tB9L%2BA%3D&amp;reserved=0) and found a few questionable variants that might arise from systematic errors in PCR (e.g., chr1:6257784, chr11:118773097, and chr1:115053498. The authors may want to remove this homopolymer bed from their variants and CTR.

*Response: We thank the reviewer for this information. We will annotate our publicly-released positive set with this finding so that the 35 positives that overlap the homopolymer-related regions will be easily identified. Users will be able to better determine given their own context and validation methods whether they wish to update their positive list and CTR applicable to their panel or assay.*

8.How did the authors decide to use these rules? "Within the CTR, a set of known positive variants (termed Class 1 variants/positives) were identified as being called in the majority of the consortium pipeline-library combinations for at least one cell line in each replicate of WES1, WES2, and WES3 (the replicated kits). For more details, see "Rules for determining positive variants" in the Laboratory and Bioinformatics Methods section."

*Response: The rules for consensus positives and negatives were discussed extensively within the consortium of professionals in academia, biotech vendors, government researchers, and genomic service providers in an international community. The final rules were achieved from consensus of informed individuals. We debated whether certain pipelines or methods should be more heavily weighted than others. In that regard, we could not reach a consensus, even with information from precisionFDA challenge results. In the end, the consortium viewed weighing results from qualified use of several pipelines as being similar to a larger ensemble-based method, which has proven advantageous in several complex probabilistic forums including variant calling (namely, SomaticSeq is an ensembl caller composed of weighing results from five different methods). In the end, we feel the final results speak for themselves related to agreement between different kits and sequencing methods and with the orthogonal ddPCR validation. More importantly, we determined that the biggest impact related to the rules for calling variants were not whether the majority of pipelines call a particular variant but in determining what genomic regions were more suited to variant calling and calling consensus with shorter read technology. In this regard, the regions selected had a bigger impact than pipelines and aligners. This was evident in the dramatic difference in agreement of different pipelines/methods inside vs. outside of high confidence or benchmark regions. We expected there to be more kit-specific bias than was actually observed. However, after removing one obvious major aspect of kit-specific bias (ie, the ROI of each kit), it was the nature of the genomic region that made the most dramatic difference in terms of reproducibility. This may seem more obvious now but it was not when we started.*

9.The authors state "To further enrich the set of identified variants, we also examined each cell line using the WGS1 linked-read method in COSMIC genes. We added several hundred variants (termed Class 2 variants/positives) to our set of Class 1 variants that were outside our CTR region but were in high- confidence coding regions of COSMIC genes. The Class 2 variants were included only if they were detected by WGS1 in at least one cell line by two different pipelines and detected by at least two WES kits. Using hg19, we identified 42,570 Class 1 and Class 2 variants in Sample A (28,064 having VAF less than 20%) with additional characteristics of these variants provided in Supplemental Table 4." This is a relatively small number of extra variants, and this linked read method will have many of the same PCR-related biases, e.g., in homopolymers and tandem repeats. Have the authors looked at these variants to determine they are accurate?

*Response: It is unclear the complete implication of "looked at" for these Class 2 variants. For the 359 Class 2 variants identified outside the CTR, we designed ddPCR assays for a random set of 50 (14% of all Class 2 variants) and all validated. This is in comparison to Class 1 where we designed and implemented assays for 114 variants chosen at random and another 100 variants that were challenging in various ways (low frequency, indels, or both). In total, we designed ddPCR assays for 14% of all identified Class 2 variants and 234 (0.5%) of all Class 1 variants. Thus, Class 2 variants were examined by ddPCR at 28x the frequency of Class 1 and 100% were orthogonally validated as being present (both classes). If the question is whether all Class 2 variants were reviewed or examined in a genomic viewer (via a BAM file), I can say there was not a systematic plan to review each Class 2 variant in this way. However, several hundred difficult variants were examined in BAM files, including Class 2 variants, some indels, and other variants that were likely MNVs. More importantly, when one examines the Class2 variants from the following two perspectives: 1) All variants including Class 2 behaved in Sample A similar to expectations from their detection in individual cell lines--that is, except for a relative handful under heavy CNA influence, Class 2 variants in Sample A had VAF roughly 1/10 times observed frequency in the individual cell lines or were roughly equal to the sum of each cell line’s VAF/10 if they were present in more than one cell lines 2) Those same variants in admixtures C, D, and E had the expected dilution frequency in ddPCR. In these senses, they were reviewed (looked-at) and deemed accurate. If this publication requires that all 359 Class 2 variants be reviewed via genomic viewing of BAM files, we will do so. However, we do not see this as necessary at this point.*

10.The authors should clearly state the limitations of this approach: "Pile-ups of each cell line were also examined to identify a large set (more than 10,000,000) of negative variant loci that were then assimilated into a collection of loci that would be absent any variant in the pooled Sample A (see "Rules for determining known negative variant positions"). Negative loci for pooled Sample A imply that if there is any somatic variant at that loci, it must have an observed AF at less than 0.25% in the pooled Sample A for each replicated kit." For example, this will exclude many places where there are systematic errors in targeted sequencing, so that methods will appear to perform better than they are. Also, they do not address challenges in representation of variants, where complex variants (nearby snps and indels) can often be represented in many different ways, e.g., at different locations within a homopolymer or tandem repeats (see https://nam04.safelinks.protection.outlook.com/?url=https%3A%2F%2Frdcu.be%2FbqpDT&amp;data=04%7C01%7CWendell.Jones%40q2labsolutions.com%7C23d30c62b21740fbdf5108d871e4983e%7C5989ece0f90e40bf9c791a7beccdb861%7C1%7C0%7C637384573912655943%7CUnknown%7CTWFpbGZsb3d8eyJWIjoiMC4wLjAwMDAiLCJQIjoiV2luMzIiLCJBTiI6Ik1haWwiLCJXVCI6Mn0%3D%7C1000&amp;sdata=0R7diwCjPpMcS1kbzvQX3bqB%2BezEDH96zpGjYA%2FthpA%3D&amp;reserved=0 )

*Response: The reviewer is correct and we agree this is a limitation of the negative positions. Within the region of 22 Mb, we identified only 10 million negative loci. As a result, a user of Sample A with a panel could underestimate the FP rate especially if a systematic bias is present. Importantly, the SEQC2 consortium acknowledges this in the revised pan-cancer panel companion paper submitted simultaneously to GB. As a result, we suggested three independent approaches for estimating the false positives. One method for FP rate estimation (termed C_only: variants called in Sample C but not in Sample A or B) could miss the systematic errors and thus also underestimate the FP rate. Another method method (B_low: variants called in Sample B of VAF below 10%) will include systematic errors. However, B_low may overestimate the FP rate. Thus, in the pan-cancer paper, we compared all three methods that are dependent on negative loci within the CTR for each cancer panel and the results were largely consistent across panels.*

*In the main text ,we have devoted a paragraph in the Discussion to the limitations of the negative positions with respect to systematic errors in targeted sequencing and a method (like B_low, mentioned previously) that may be used to complement a simple false positive rate based on the known but potentially biased negative positions that would not “hide” systematic sequencing errors.*

11.The authors should perform statistical tests or give confidence intervals to support several of the claims made in this section: "Using SomaticSeq, we confirmed greater than 99.5% of the Class 1 and 2 variants identified from the individual cell line analysis were at or near the expected VAF in Sample A using either an in silico version of Sample A or the merged-BAM Sample A (see Supplemental Table 6). In addition, although the WES4 Thermo Fisher runs had only one replicate per cell line and were not as deeply sequenced as WES1-3, over 97.8% of the CTR positives with Sample A VAF > 5% were detected in at least one cell line. Additional information on WES4-related sensitivity and precision of individual cell lines are provided in Supplemental Fig. 2."

*Response: The information that we confirmed 99.5% of the identified variants were at or near the expected VAF (and other similar claims in that section) is an observation (i.e., it is descriptive). In statistical tests, one generally is making inferences about the true values of unknown population parameters such as the mean and variance of a population based on a statistical sample. In this case, our entire population is the ~42k variants identified. We are not making inferences about variants yet to be found. Since we are reporting results on all 42,570 variants in the defined population, there is no unknown variability in the likelihood value, thus confidence intervals are not appropriate nor is a statistical test appropriate. We can discuss whether there are remaining positives in Sample A (obviously there are, especially outside of the CTR region), but our statistical sample of variants related to that population would be biased. So again, statistical tests and confidence intervals would not be appropriate as we do not wish to infer anything at this point beyond the 42,570 variants identified in the study.*

12.The authors state: "Finally, we used droplet digital PCR (ddPCR) technology to orthogonally validate a large number of positives and negatives from different classes of variants in individual cell lines, reference Sample A, and normal Sample B. We also orthogonally tested those same variants using admixture reference samples C, D and E where Sample C is a 1:1 dilution of A:B, Sample D is a 1:4 dilution, and Sample E is a 1:24 dilution. The validated variants included more difficult-to-detect variants such as 50 smaller insertions-deletions (indels) and more than 100 low frequency variants (VAF ~ 1-5% in pooled Sample A) as well as 50 variants randomly sampled from the larger Class 2 positive set derived from COSMIC genes. In total, we identified nearly 300 positive and 39 negative loci within our known positive and negative set for independent ddPCR testing (Supplemental Table 7)". Could the authors be more explicit about the variants that could not be tested by ddPCR because assays couldn't be designed? For example, are there variants in difficult to map regions or homopolymers or tandem repeats that failed design?

*Response: When an assay could not be designed with BioRad’s help for a particular variant, we chose another variant from the same category. To avoid confusion, we revised the last sentence to be “The 284 positive and 39 negative loci within our known positive and negative set were randomly selected for independent ddPCR testing (Supplemental Table 7).” The selection of targets for ddPCR validation was random among each categories of candidate targets. Please see the Methods for detailed description of each category. As ddPCR assays are suited for testing biallelic targets, we have included this requirement in our initial collection of candidate targets: “iii) targets with close-by variant(s) were avoided in order to minimize interference with the primer or probe of the ddPCR assay.” We then submitted the candidate targets one by one to Bio-Rad Web Portal for assay design. For any target that failed this online design process, we simply ignored it and replaced it with another candidate from the same category. In the end, we retained the targets with valid design to approximately meet the preset goal of ddPCR assays for each category. For several targets that failed design, we took a close examination and even consulted Bio-Rad for assistance. However, we were not able to pinpoint the explicit reason for the design failures.*

13.The authors state: "We recommend the use of tissue-cultured cells grown in a large batch in strictly controlled environments that will minimize any batch-to-batch variability and allow use in the community for several years." Did they actually do this for this work?

*Response: Yes! For the SEQC2 study, one large batch of cells were grown at standard tissue-culture conditions (RPMI media, 10% FBS, 1x Pen/Strep) to 10e8 cells each. Total gDNA yield from 10 cell lines was ~ 3mg (300ug x 10 cell lines) in a laboratory that has almost two decades experience working with these cells. Historically and in comparison, Agilent has made 3 batches of the Universal Reference RNA (UHRR) during ~ 16 years with high batch-to-batch reproducibility. Each batch was prepared using ~ 10e9 cells (each cell line).*

14.The authors state: "Reasons for these differences include HG001 receiving a higher initial degree of manual review as well as more time related to review (HG001 was the first specimen having this data released publicly). We chose a more conservative approach for ground truth and created a common high confidence region based on overlapping regions of HG002, 003, 004, and 005 to serve as the basis for high confidence regions for this initial release of positives and negatives. In addition, we excluded a small amount of low complexity regions28 overlapping with this more universal high confidence region which served as the basis for both Class1 and Class 2 variant detection." It isn't exactly true that the differences for HG001 result from manual review. It is more related to the greater amount of data for HG001 available at the time (now HG002 has more data in https://nam04.safelinks.protection.outlook.com/?url=https%3A%2F%2Fdoi.org%2F10.1101%2F2020.07.24.212712&amp;data=04%7C01%7CWendell.Jones%40q2labsolutions.com%7C23d30c62b21740fbdf5108d871e4983e%7C5989ece0f90e40bf9c791a7beccdb861%7C1%7C0%7C637384573912655943%7CUnknown%7CTWFpbGZsb3d8eyJWIjoiMC4wLjAwMDAiLCJQIjoiV2luMzIiLCJBTiI6Ik1haWwiLCJXVCI6Mn0%3D%7C1000&amp;sdata=4a7NSYXPshBnDPaY5%2FFBipW7EwdIhBTN7ehmoY5Bats%3D&amp;reserved=0), and because it included chrX if the authors included this.

*Response: The text has been altered to read "However, the high confidence region for HG001 was noticeably different than the other four in terms of size: HG001 benchmark regions were noticeably larger by 3%-4% (even after excluding chrX regions exclusive to HG001). Reasons for these differences are discussed in the NIST high confidence regions (v3.3.2 benchmarking regions) Methods section" in the main body. We also altered the NIST high confidence region (v3.3.2 benchmarking region) section to read "The HG001 genome (aka NA12878) had additional data from more sequencing platforms and additional review to identify extended regions from the initial consensus for what was viewed as “high-confidence” calls for that genome. Due to this special case of additional data and review of the high-confidence regions, the SEQC2 consortium instead examined the high-confidence regions for HG002, HG003, HG004, and HG005...". We welcome alternative wording if provided.*

15.The authors state: "Variants from WGS 10X Chromium libraries of individual cell lines were examined in high confidence coding regions of COSMIC genes but outside of CTR regions that includes common design coding regions to all WES kits utilized in this study. If the same variant was called by two different WGS variant callers (Freebayes and GATK) and was called by SomaticSeq from two different WES kits in the same cell line, then the variant was categorized as a Class 2 positive variant." What aligner did they use, and why didn't they use the Long Ranger mapper and variant caller?

*Response: In the original manuscript, we stated in the Methods for Lib prep and sequencing of WGS1 that the aligner used was Long Ranger wgs version 2.2.2. The Long Ranger software itself provides options to use Freebayes or GATK to call variants. In this case, we used both and required agreement for additional stringency before taking the variant candidate forward for putative variants outside the CTR.*

16.The authors state: "Any positions with total DP >= 500, ADP > 2 and AF >= 0.002 were removed. " Is there a statistical basis for this, e.g., confidence interval for max allele frequency?

*Response: No, this was strictly a heuristic. However, we did not use this heuristic in a silo. In two companion papers, one of which was submitted to GB related to pan-cancer panels, we examined using the negative loci alone to estimate the false positive rate vs. two other orthogonal techniques, one of which would not be subject to hiding systematic sequencing errors. Although the FP rate estimated by the negatives in Sample A tended to be slightly smaller than the value provided by the other two methods, it was very consistent in ranking panel performance of panels with the other two methods. Given that result, which was being analyzed in parallel, we decided that the rule had enough utility to be implemented as is. More importantly, the other two methods for estimating the false positive rate were indirect (ie, they were not direct measurements of negative regions of Sample A) and thus did not provide an alternative set of negative positions for Sample A.*

17.The authors state: "The adjacent positions from both end of the INDEL calls were also removed." Why only the adjacent positions? As stated above, different representations of indels can cause many problems for this. GIAB now excludes 50bp from either end of an uncertain variant plus any overlapping tandem repeats or homopolymers (<https://nam04.safelinks.protection.outlook.com/?url=https%3A%2F%2Fdoi.org%2F10.1101%2F2020.07.24.212712&amp;data=04%7C01%7CWendell.Jones%40q2labsolutions.com%7C23d30c62b21740fbdf5108d871e4983e%7C5989ece0f90e40bf9c791a7beccdb861%7C1%7C0%7C637384573912665933%7CUnknown%7CTWFpbGZsb3d8eyJWIjoiMC4wLjAwMDAiLCJQIjoiV2luMzIiLCJBTiI6Ik1haWwiLCJXVCI6Mn0%3D%7C1000&amp;sdata=BC2VaEkezfDnOX5Yn%2B7WS9TZMm4ryBFNfflWpVOePzI%3D&amp;reserved=0>).

*Response: We thank the reviewer for the reference to the biorxiv in-progress work on "Benchmarking challenging small variants with linked and long reads" which we have included in our references. We note that the document does describe a process for removing entries from a VCF that removes 50 bp on either side of multi-allelic entries from PacBio HiFi and 50 bp on each side of filtered indels from 10x Genomics output. However, this seems to be describing part of a process for creating regions that are "callable" similar to the intent of our Consensus Target Region. In this case, the reviewer is commenting on a region that we have already identified to be relatively safe for variant calling (i.e., inside the CTR) and we are simply identifying, within that region, particular loci where we have evidence that there is no local variant, rather than whether a variant is callable. It is not clear that the same ruleset should be applied in this case as we have not said that the indel is problematic or complex, which is possibly implied by the referenced document which describes removing 50bp on either side of each “filtered” indel. Supposedly, a filtered indel is considered problematic.*

18.The authors state: "While some models appeared more reasonable than others, we observed large variations in the estimates for the cell line mix ratios for different models and subsets, resulting in fundamentally unreliable VAF predictions. The instability in estimates was possible due to the large number of rearrangements in the cancer cell lines, creating inconsistent depth at a given locus from cell line to cell line. A simple linear regression of the VAF of each cell line onto the Sample A VAF provided reasonable if oversimplified results that indicated the cell lines were properly mixed (given the approximate 1:2 mixture of TLY into BLY described elsewhere)." The different ploidies of the cell lines is a major limitation that should be highlighted very clearly by the authors, since this could cause problems for some sequencing technologies as well as for the mixture designs.

*Response: We have added discussion of ploidies of the ten cancer cell lines to the discussion. We do not agree that the different ploidies create a major limitation but do instead create a complication for certain analyses and the tracking of ploidies in different future iterations or versions of the reference sample. We have observed that ploidy variation in the individual cell lines create no worse variation in overall read depths in Sample A compared to what is already occurring due to the nature of the bait-based WES assays (based on comparing the distribution of read depths in variants common between the normal Sample B which only has bait bias impacting local depth and Sample A which has both bait bias and ploidy variation). However, we should also state that we are not suggesting that this reference Sample A be used a reference DNA sample to detect CN changes or large structural variants such as inversions. That assessment is better suited with using matched cancer cell line-normal pairs. We maintain that Sample A is very well suited for assessing SNV and small indel detection performance. If a new iteration of Sample A results in a variant increasing VAF due to increased ploidy in one cell line or going away (ploidy-level loss in the cell line), this can be easily detected in the further mutated cell line and new iteration of Sample A and then documented.*

19.The authors state "One easily observes that the VAF for ddPCR is much larger especially when the VAF approaches .50 (50%) and continues as a rather constant difference of roughly 2% in absolute VAF value for SNVs in a range of VAF [.35, .9]. Alternatively, the distinction for indels is even larger, when many variants achieving a 5% greater VAF (in absolute terms) from ddPCR vs. WES estimates. We conjecture that this bias is due to capture baits as 1)capture baits are typically designed from reference sequence, 2)the magnitude of the difference for indels was observed to be related to the bases inserted or deleted (data not shown), implying an interaction between the difference in detected sequence vs. reference sequence and the magnitude of that deviation from expected VAF, and 3)we do not observe a bias when examining WGS results and specifically the het state of normal cell lines while the WES results yield VAF estimates from those same cell lines indicating the mode for hets is typically around .48 (rather than .50) across kits and cell lines." Did the authors have sufficient depth from WGS to make this claim? I would have expected this is a simple mapping bias for indels, which has frequently been reported.

*Response: We agree that we do not necessarily have sufficient depth in the WGS to make this claim. We are rewording the last statements in that section to read "There are possibly multiple contributors to this bias with reference mapping bias as a likely primary contributor. We note the WES of individual cell lines typically has a VAF mode of 0.48 (consistent with the bias of 0.02 of the large majority of SNVs as seen in the Sup. Figure 10) while the WGS mean VAF for hets observed in Sample B (normal cell line) was slightly higher at 0.488."*

20.The authors state: "Important data items from the consortium reference material effort that we intend to disclose…". As stated above, I am concerned that these samples aren't properly consented for these data to be made public.

*Response: This is addressed in the General Response discussion at the beginning of the response.*

21.Could the authors add if this is a potential Agilent product to "Natalia Novoradovskaya, Katherine Wilkins, Anne Lucas, Raymond Miller, Carlos Pabon are all employees of Agilent Technologies, Inc."?

*Response: Agreed and this statement has been added.*

22.In Table 1: Please give more details for this: "CNA were estimated using Agilent GenetiSure Cancer Research CGH+SNP Microarray (2x400K), G5975A and WES."

*Response: We thank the reviewer for noting this and a CN methods section has been added in the Methods section of the manuscript.*

23.In Table 2, specify whether this is just Class 1 or Class 1&2

*Response: This information has been added to the legend.*

24.In Figure 2, change "NIST high-confidence regions" to "NIST v3.3.2 benchmark regions", specifying the version and the current terminology, which is important for understanding the results

*Response: This has been done. Since the literature refers to these as both benchmark and high-confidence regions, I have kept both associations.*

25.In Figure 3a, could the authors clarify why n=1242 when they only had 375 ddPCR assays?

*Response: Each point in Figure 3a is a cell line-assay combination. There are 375 ddPCR assays. However, 19+9+24=52 were investigational (or speculative) and not graphed leaving 284 positive + 39 negative (323 total) ddPCR assays to interrogate the 10 individual cell lines for Figure 3a (the other graphs 3b, 3c, and 3d are about one or more of the composites A,C, D, and E as well as potentially Sample B). While we did include results and graph each of the negatives for each cell line (39*10=390 points) in the graph, the remaining 1242-390 = 852 assays were distributed among the 284 positives for only the cell lines (out of 10--average was 2.96) that detected the variant. Otherwise, we would have overpopulated the origin (0,0) with nearly 2000 negative results related to variants that are positive in other cell lines and thus, inflated the r-squared value (visually, it would have had no impact to graph these 2000 negative results). So, in short, aside from the 390 confirmed negatives (39*10), only positives of Sample A that are also positive in their cell line of origin (where the variant was also positive) were graphed.*

26.In Figure 3, it would be useful also to have histograms of the VAF from WES for all class 1 and all class2 variants, both in the cell lines and in Sample A.

*Response: In the originally submitted manuscript and resubmission, Sup Figure 6a/b and 7a-j are the histograms for all identified variants both in Sample A and B and for each of the 10 cell lines. These graphs include both Class 1 and Class 2 variants. If we restrict to simply Class1 variants, these graphs will appear nearly identical as the Class 2 variants comprise < 1% of all identified positives, thus <1% of the content of the histograms. Class 2 variants only become prominent when examining the COSMIC genes which are roughly 3% of all genes. Nevertheless, in the resubmission and response, we have included histograms 7k-7t in the Supplemental Figures which are VAF histograms for Class 2 variants in the individual cell lines and histogram 6c which are the Class 2 variant VAFs for Sample A. It is unclear how this would relate to Figure 3 directly as Figure 3’s contents are only those variants orthogonally validated by ddPCR.*

27.In Figure 4, why are there very few "Known Negatives" in the first 2 exons?

*Response: We believe that the reason for regions having a negative “desert” in general is WES2. WES2 reported low coverage much more frequently than WES1 and WES3 in various regions. As stated in methods, any cell line for any of WES1-WES3 for either replicate library that had a DP < 50 was removed as a candidate for negative positions. This lack of coverage was partially stochastic in nature, possibly due to variable bait affinity, so it is by chance in combination with (potentially) bait bias that the first few exons in Figure 4 have relatively few "Known Negatives". The negatives are subject to several competing conditions being appropriate. We look forward to extending the negatives for this set of cells in future work.*

**Second round of review**

**Reviewer 1**

The authors addressed most of my previous concerns and the manuscript has been greatly improved. I appreciate the authors’ efforts. I now have only one concern about the subclonal mutations in the original cell line relevant to my previous question #4. While the answer from authors provided a practical solution to maintain the consistency among batches, there is a remaining concern about the reliability of the mutation call. In my experience, some of these calls were false positives originated from alignment error. I strongly recommend checking the variant calling and filtering process on these calls as these control positives should be truly accurate. There are several strategies for it including (1) comparison between alignment scores of ref- and alt- reads, (2) inspecting inclusion of secondary alignment.

**Reviewer 2**

The authors have done a good job improving the manuscript based on the reviewers’ comments and answering the comments in detail. I have only a few remaining follow-up suggestions, with the most important one being the first about the individuals’ consent:

1. A significant part of this resource is the public data, so it is great that this will be released in the future. Because of the sensitivity of genomic data, I think it is important that more information is given about the consent of these individuals for public dissemination of genomic data, particularly given the history of cell lines like HeLa that lacked informed consent.

2. In their response to point 12, the authors state: "When an assay could not be designed with BioRad’s help for a particular variant, we chose another variant from the same category. To avoid confusion, we revised the last sentence to be “The 284 positive and 39 negative loci within our known positive and negative set were randomly selected for independent ddPCR testing (Supplemental Table 7).” The selection of targets for ddPCR validation was random among each categories of candidate targets. Please see the Methods for detailed description of each category. As ddPCR assays are suited for testing biallelic targets, we have included this requirement in our initial collection of candidate targets: “iii) targets with close-by variant(s) were avoided in order to minimize interference with the primer or probe of the ddPCR assay.” We then submitted the candidate targets one by one to Bio-Rad Web Portal for assay design. For any target that failed this online design process, we simply ignored it and replaced it with another candidate from the same category. In the end, we retained the targets with valid design to approximately meet the preset goal of ddPCR assays for each category. For several targets that failed design, we took a close examination and even consulted Bio-Rad for assistance. However, we were not able to pinpoint the explicit reason for the design failures.”

- My understanding of the authors’ categories is that they would not require that indels in homopolymers would be validated, which is the class I’d identified as containing likely problematic variants in the positive set. If no or few indels in homopolymers were validated, that’s ok, but this should be stated clearly.

3. In their response to point 17, the authors state: "We thank the reviewer for the reference to the biorxiv in-progress work on "Benchmarking challenging small variants with linked and long reads" which we have included in our references. We note that the document does describe a process for removing entries from a VCF that removes 50 bp on either side of multi-allelic entries from PacBio HiFi and 50 bp on each side of filtered indels from 10x Genomics output. However, this seems to be describing part of a process for creating regions that are "callable" similar to the intent of our Consensus Target Region. In this case, the reviewer is commenting on a region that we have already identified to be relatively safe for variant calling (i.e., inside the CTR) and we are simply identifying, within that region, particular loci where we have evidence that there is no local variant, rather than whether a variant is callable. It is not clear that the same ruleset should be applied in this case as we have not said that the indel is problematic or complex, which is possibly implied by the referenced document which describes removing 50bp on either side of each “filtered” indel. Supposedly, a filtered indel is considered problematic.”

- I agree that this is different from excluding uncertain calls, but the reasoning is similar, so I should have been more clear. In this work, the authors try to define regions without a variant, and my concern is that indels can often be represented at different positions, particularly in homopolymers and tandem repeats. In these cases, an indel can be placed at any position within the homopolymer or tandem repeat, so that they should not be considered to be non-variant. Simple cases can be ameliorated by left-alignment, but more complex cases cannot, so that is the reasoning behind the ~50bp exclusion. Technically, GIAB’s benchmarks are more sophisticated than this, and actually exclude the entire repeat. Note that I’m suggesting this because the authors don’t appear to use the approach GIAB has used, where GIAB defines benchmark variants and benchmark regions, and FPs are any extra variants from the query inside the benchmark regions but not matching the benchmark variants, when using sophisticated comparison tools.

**Authors Response**

**Point-by-point responses to the reviewers’ comments:**

Reviewer #1: The authors addressed most of my previous concerns and the manuscript has been greatly improved. I appreciate the authors’ efforts. I now have only one concern about the subclonal mutations in the original cell line relevant to my previous question #4. While the answer from authors provided a practical solution to maintain the consistency among batches, there is a remaining concern about the reliability of the mutation call. In my experience, some of these calls were false positives originated from alignment error. I strongly recommend checking the variant calling and filtering process on these calls as these control positives should be truly accurate. There are several strategies for it including (1) comparison between alignment scores of ref- and alt- reads, (2) inspecting inclusion of secondary alignment.

*Response: We agree that the reliability of the variant call is very important. We have worked very diligently to ensure that our positives are truly positive. Here are the major steps we have taken in that effort:*

1. *Our positives are required to be in high-confidence NIST benchmark regions of the genome, which are also not in low complexity regions.*
2. *Our positives have to be identified either by all three major WES kits, or in those regions where all three do not have overlapping targets, have to be identified by two of the WES kits and by two callers from our linked-read WGS using LongRanger alignment.*
3. *Using ddPCR, we orthogonally tested 284 putative positive variants selected by randomization with stratification to examine mostly challenging indels and SNVs of different categories including low-frequency SNVs, and all (100%) were validated.*
4. *Reviewer 2 indicated some homopolymer regions near 35 of our 42,570 positive variants (<0.1%). We examined each of these variants in detail using pile-ups examining ref/alt reads, strandedness, mapping parameters, and other factors and found only 1 of the 35 (or < 0.005% of the total positive set) to be in a difficult-to-resolve state. Nevertheless, we flagged all 35 variants in our documentation as being near these homopolymer regions even though we are very confident in 34 of the 35 and feel the remaining variant is likely a positive but in a difficult to call region.*
5. *We cross-referenced these 35 variants and other difficult variants with the WGS long-read data by cell line and the only variants not detected by WGS were a handful below the WGS LOD (95%ile LOD for WGS was roughly VAF=30% due to WGS average depth of around 65x-85x by cell line).*
6. *We examined several hundred other variants at low frequency and/or in close proximity to other variants to better characterize them in terms of haplogroups, MNVs, and potential false positives. None were deemed suspicious as variants although for a subset we have updated the annotations to indicate their possible classification as MNVs.*
7. *Indels are a more difficult class of variants to properly detect than SNVs for a variety of reasons. We have been conservative in our indel identification by requiring the majority of distinct pipeline runs identify the same indel so as to minimize false positives. As a result, only 1.3% of our positives are indels whereas the overall population is possibly 2-5x times that depending on tumor type.*

*For these reasons, we believe this collection of 42,570 variants have very high fidelity and have more accurate VAF estimates than even commercially available reference samples that have many fewer variants (some have only tens of variants), given my personal review of testing and validation results of these samples using multiple independent technologies.*

Reviewer #2: The authors have done a good job improving the manuscript based on the reviewers’ comments and answering the comments in detail. I have only a few remaining follow-up suggestions, with the most important one being the first about the individuals’ consent:

1. A significant part of this resource is the public data, so it is great that this will be released in the future. Because of the sensitivity of genomic data, I think it is important that more information is given about the consent of these individuals for public dissemination of genomic data, particularly given the history of cell lines like HeLa that lacked informed consent.

*Response: We understand the request. We agree that consent is an important issue related to public dissemination of data. In this case, we received these materials from a commercial provider of sample material designated as RUO purposes only. Therefore, we feel any statements about consent are best left to the commercial supplier. The reviewer’s request reflects a much larger issue than what this manuscript can address. The consortium has taken reasonable precautions in protecting privacy through non-disclosure agreements and not releasing germline-related data regarding any individual. By only releasing data for the pooled material or only releasing somatic variants, we protect individual identifiable information.*

2. In their response to point 12, the authors state: "When an assay could not be designed with BioRad’s help for a particular variant, we chose another variant from the same category. To avoid confusion, we revised the last sentence to be “The 284 positive and 39 negative loci within our known positive and negative set were randomly selected for independent ddPCR testing (Supplemental Table 7).” The selection of targets for ddPCR validation was random among each categories of candidate targets. Please see the Methods for detailed description of each category. As ddPCR assays are suited for testing biallelic targets, we have included this requirement in our initial collection of candidate targets: “iii) targets with close-by variant(s) were avoided in order to minimize interference with the primer or probe of the ddPCR assay.” We then submitted the candidate targets one by one to Bio-Rad Web Portal for assay design. For any target that failed this online design process, we simply ignored it and replaced it with another candidate from the same category. In the end, we retained the targets with valid design to approximately meet the preset goal of ddPCR assays for each category. For several targets that failed design, we took a close examination and even consulted Bio-Rad for assistance. However, we were not able to pinpoint the explicit reason for the design failures.”

- My understanding of the authors’ categories is that they would not require that indels in homopolymers would be validated, which is the class I’d identified as containing likely problematic variants in the positive set. If no or few indels in homopolymers were validated, that’s ok, but this should be stated clearly.

*Response: We have modified our text related to independent verification by ddPCR to say “While we identified a small number of variants near homopolymer regions (less than 0.1% of the total),* *we did not verify those variants independently using ddPCR but did ensure that all variants near homopolymer regions and having VAF above the limit of detection of linked-read WGS were detected by the linked-read WGS.“*

3. In their response to point 17, the authors state: "We thank the reviewer for the reference to the biorxiv in-progress work on "Benchmarking challenging small variants with linked and long reads" which we have included in our references. We note that the document does describe a process for removing entries from a VCF that removes 50 bp on either side of multi-allelic entries from PacBio HiFi and 50 bp on each side of filtered indels from 10x Genomics output. However, this seems to be describing part of a process for creating regions that are "callable" similar to the intent of our Consensus Target Region. In this case, the reviewer is commenting on a region that we have already identified to be relatively safe for variant calling (i.e., inside the CTR) and we are simply identifying, within that region, particular loci where we have evidence that there is no local variant, rather than whether a variant is callable. It is not clear that the same ruleset should be applied in

this case as we have not said that the indel is problematic or complex, which is possibly implied by the referenced document which describes removing 50bp on either side of each “filtered” indel. Supposedly, a filtered indel is considered problematic.”

- I agree that this is different from excluding uncertain calls, but the reasoning is similar, so I should have been more clear. In this work, the authors try to define regions without a variant, and my concern is that indels can often be represented at different positions, particularly in homopolymers and tandem repeats. In these cases, an indel can be placed at any position within the homopolymer or tandem repeat, so that they should not be considered to be non-variant. Simple cases can be ameliorated by left-alignment, but more complex cases cannot, so that is the reasoning behind the ~50bp exclusion. Technically, GIAB’s benchmarks are more sophisticated than this, and actually exclude the entire repeat. Note that I’m suggesting this because the authors don’t appear to use the approach GIAB has used, where GIAB defines benchmark variants and benchmark regions, and FPs are any extra variants from the query inside the benchmark regions but not matching the

benchmark variants, when using sophisticated comparison tools.

*Response: We appreciate the reviewer's comment aiming to ensure the accuracy of negative positions around indels in the homopolymers or tandem repeats. It is worthy pointing out some differences between GIAB's approach and ours in determining regions without a variant. Such "negative" regions can then be used to flag false positives. The reviewer stated, "FPs are any extra variants from the query inside the (GIAB) benchmark regions but not matching the benchmark variants, when using sophisticated comparison tools." This implies that any position within the benchmark regions except those of the benchmark variants is considered a negative position without a variant. The negative regions were inferred from the benchmark variants. In our approach, we only identified a portion (~50%) of our benchmark region (i.e., the Consensus Targeted Regions which are high confidence exonic coding regions) as known negatives in Sample A. The CTR was already a subset of NIST high-confidence regions (v3.3.2 benchmark regions). Low complexity regions were also excluded. Most homopolymers and tandem repeats were expected to be excluded through these two requirements. We then examined the sequencing data across three WES kits and two replicates (in silico Sample A and pooled Sample A) to determine the negative positions. As demonstrated in our companion cross-lab oncopanel manuscript accepted by Genome Biology, of eight pan-cancer oncopanels, none of putative false positives identified through our known negatives was reproducible in over 50% of library replicates. Greater than 99% of them were reported in less than 4 libraries out of 12 replicates. Our approach was based on the evidence of sequencing data and also validated by the parallel oncopanel study.*
